# Nickel-catalyzed electrochemical carboxylation of unactivated aryl and alkyl halides with CO_2_

**DOI:** 10.1038/s41467-021-27437-8

**Published:** 2021-12-06

**Authors:** Guo-Quan Sun, Wei Zhang, Li-Li Liao, Li Li, Zi-Hao Nie, Jin-Gui Wu, Zhen Zhang, Da-Gang Yu

**Affiliations:** 1 Key Laboratory of Green Chemistry & Technology of Ministry of Education, College of Chemistry, Sichuan University, Chengdu, 610064 China; 2 College of Pharmacy and Biological Engineering, Chengdu University, Chengdu, 610041 China; 3 Beijing National Laboratory for Molecular Sciences, Beijing, 100190 P. R. China

**Keywords:** Homogeneous catalysis, Synthetic chemistry methodology

## Abstract

Electrochemical catalytic reductive cross couplings are powerful and sustainable methods to construct C−C bonds by using electron as the clean reductant. However, activated substrates are used in most cases. Herein, we report a general and practical electro-reductive Ni-catalytic system, realizing the electrocatalytic carboxylation of unactivated aryl chlorides and alkyl bromides with CO_2_. A variety of unactivated aryl bromides, iodides and sulfonates can also undergo such a reaction smoothly. Notably, we also realize the catalytic electrochemical carboxylation of aryl (pseudo)halides with CO_2_ avoiding the use of sacrificial electrodes. Moreover, this sustainable and economic strategy with electron as the clean reductant features mild conditions, inexpensive catalyst, safe and cheap electrodes, good functional group tolerance and broad substrate scope. Mechanistic investigations indicate that the reaction might proceed via oxidative addition of aryl halides to Ni(0) complex, the reduction of aryl-Ni(II) adduct to the Ni(I) species and following carboxylation with CO_2_.

## Abstract (web summary)

Electrochemistry is a promising approach to make existing chemical protocols milder, but many simple transformations of feedstocks are still out of reach. Here, the authors transform unactivated aryl and alkyl (pseudo)halides into carboxylic acids, via nickel catalysis and electricity, using atmospheric CO_2_ as the carbon source.

## Introduction

Reductive cross couplings represent powerful methods to generate C−C bonds by circumventing extra synthetic steps and handling moisture-sensitive organometallic reagents^1^. Much progress has been achieved in last decades via transition metal catalysis^2,3^ and photocatalysis^4^. Recently, significant advance of electrochemistry^5–20^ has attracted much attention with electron as clean reductant, providing a sustainable solution for chemical synthesis^5,6^. Compared to direct electrochemistry, electrocatalysis is more attractive and useful due to the lower potential and better functional group tolerance^11–14^. Notably, electrochemical transition metal-catalysis^15–20^, especially Ni-catalysis^20^, is efficient and powerful in reductive cross couplings. Electro-reductive Ni-catalyzed coupling of aryl halides with organo (pseudo)halides^21^ and carbonyl-containing compounds^22^ has emerged as a convenient strategy. However, most of such cases are limited to activated aryl halides, which bear electron-withdrawing groups (EWGs) or electron-poor heteroarenes. The electrocatalytic reductive cross couplings of unactivated aryl halides, especially unactivated aryl chlorides, are challenging tasks to resolve^23^.

**Fig. 1:**
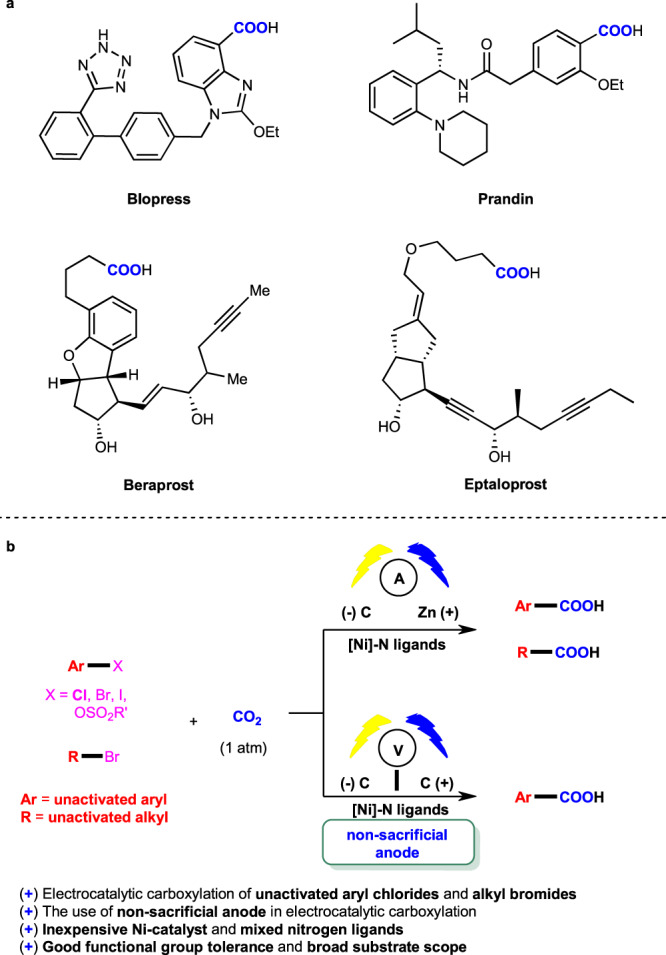
Background and synopsis of our work. **a** Selected examples of bioactive compounds containing carboxylic acid moiety. **b** A general electrocatalytic carboxylation of unactivated organo (pseudo)halides with CO_2_.

Carboxylation of organohalides with CO_2_ is a direct and effective way to construct valuable carboxylic acids (Fig. 1a) due to the abundant, nontoxic and recyclable properties of CO_2_^24–30^. However, the thermodynamic stability and kinetic inertness make it difficult to achieve high efficiency under mild reaction conditions. It is even more challenging when the inexpensive coupling partners are less reactive. Although recent progress has shown some robust catalytic manners in transition-metal catalysis^31,32^ and photocatalysis^33–38^, the use of stoichiometric amount of extra metallic reductants, expensive Ir-photosensitizer, or reactive organohalides still limites their wide application. Meanwhile, electrocatalytic carboxylation of activated aryl (pseudo) halides^22,39–44^, benzylic and allylic ones^45^ have been investigated. However, selective electrocatalytic carboxylation of unactivated aryl chlorides and unactivated alkyl halides with CO_2_ have rarely been realized^46–50^, which are more challenging due to the low reactivity of both unactivated organohalides and CO_2_ as well as many competing side reactions, such as protonation, *β*-H elimination, migration, and homodimerization of the generated organometallic reagents and electrochemical decarboxylation of the products^51^. Besides the limitations in substrate scope, the reductive electrochemical carboxylations mainly rely on the use of sacrificial electrodes^52–56^.

Here, we show a general and practical electrochemical Ni-catalytic system, realizing the electrocatalytic carboxylation of challenging aryl chlorides and unactivated alkyl bromides with CO_2_ (Fig. 1b). A variety of unactivated aryl bromides, iodides, and sulfonates also undergo such a reaction smoothly. Moreover, the catalytic electrochemical carboxylation of aryl (pseudo)halides with CO_2_ avoiding the use of sacrificial electrodes is also developed.

## Results

### Optimization of Ni-catalyzed electrochemical carboxylation

We initiated our studies by exploring electro-reductive Ni-catalytic carboxylation of 4-chlorobiphenyl **1a** with CO_2_ (see Supplementary Table 1). Considering that aryl chlorides are less reactive than aryl bromides or iodides, we proposed that a highly reactive Ni-catalytic system should be developed to improve the challenging oxidative addition under mild reaction conditions. We decided to explore proper ligand to keep the Ni-catalyst active and increase the catalytic efficiency. After systematic condition screening, we were delighted to realize the carboxylation of **1a** to give **2a** in 70% yield in the undivided cell by using Ni-catalyst and mixed nitrogen ligands, which contained ditertbutylbipyridine (L, dtbbpy) and 4-dimethylaminopyridine (DMAP) (Supplementary Table 1, entry 1). Both of these two ligands were important as no or low efficiency was observed in the absence of either one (Supplementary Table 1, entries 2 and 3). Besides, Lewis acidic MgBr_2_ also promoted the reaction (Supplementary Table 1, entry 4), which might coordinate with CO_2_ and increase its electrophilicity^57,58^. Control experiments verified the essential role of nickel catalyst, CO_2_, and electricity (Supplementary Table 1, entries 5–7). The use of potassium *tert*-butoxide (KO^*t*^Bu) had improvement for the carboxylation (Supplementary Table 1, entry 8). Supporting electrolyte also had a significant effect on the reaction (Supplementary Table 1, entry 9). Varying from other parameters, such as solvent and electric current, did not give better results (Supplementary Table 1, entries 10–12).

### Substrate scope

**Fig. 2:**
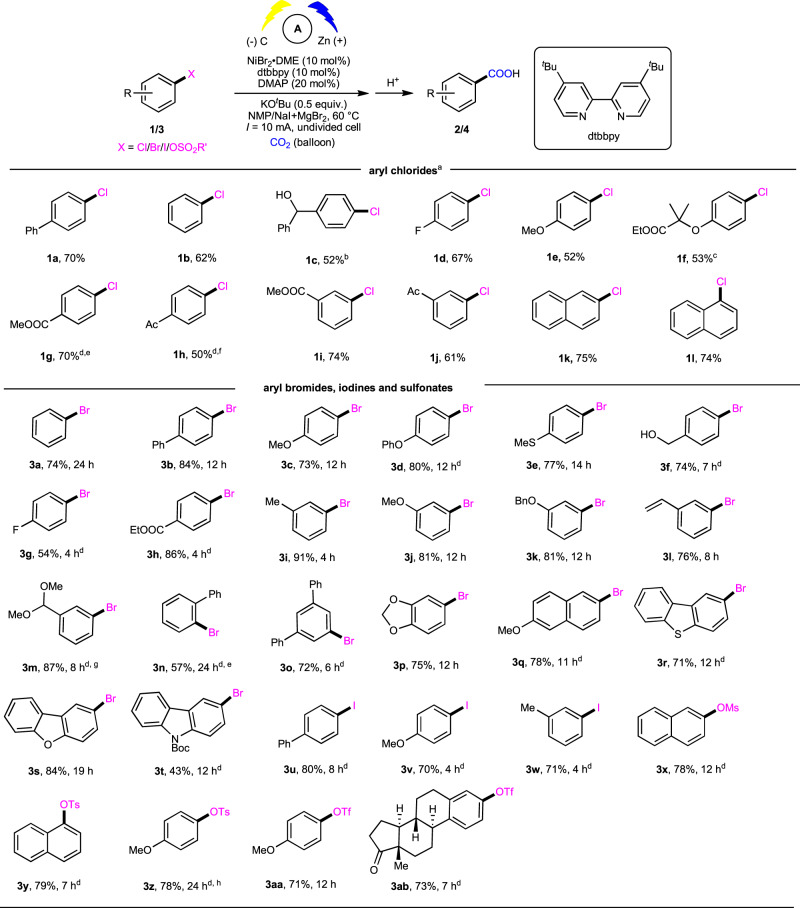
Scope of aryl chlorides, bromides, iodines, and sulfonates. ^a^Reaction conditions, see more details in “Supplementary information”. ^b^5 Å molecular sieve was added. ^c^NiBr_2_•DME (5 mol%), dtbbpy (5 mol%), DMAP (10 mol%). ^d^Ni(acac)_2_ (5 mol%), dtbbpy (5 mol%), KO^*t*^Bu (0.5 equiv), I = 8 mA. ^e^50 °C, 8 h. ^f^Room temperature, 12 h. ^g^3-formylbenzoic acid was obtained as product. ^h^I = 4 mA.

With the optimal reaction conditions in hand, we first explored the scope of aryl chlorides (Fig. 2). A variety of unactivated aryl chlorides underwent the carboxylation smoothly to give the desired acids in moderate to good yields (**1a**-**1f**). Notably, the substrate **1c** bearing free alcohol, which might undergo oxidation via β-H elimination^59^ or coupling via C−O bond activation^60^, was not reported in previous carboxylations with CO_2_^31,33^. Deactivated aryl chlorides (**1e** and **1f**) bearing with electron-donating groups (EDGs) at the *para* position, which show no or lower reactivity in previous carboxylations^31–38^, were also amenable to our reaction. Notably, clofibrate (**1f**), a commercially available drug, underwent the reaction to afford **2f** in 53% yield, which might be applied in drug discovery to improve the hydrophilicity and metabolism of drug molecules. As electron-deficient aryl chlorides were more reactive coupling partners, they could take part in the reaction with lower catalyst loading and temperature in the absence of either DMAP or MgBr_2_. Notably, this electrochemical approach exhibited good tolerance to diverse functional groups, including hydroxyl (**1c**), fluoro (**1d**), methoxyl (**1e**), ester (**1f**, **1g**, and **1i**), and ketone (**1h** and **1j**). Besides substituted phenyl chlorides, 2-naphthyl and more sterically hindered 1-naphthyl chlorides were also suitable substrates in our reaction. The substrates with more steric hindrance, such as 2-methylphenyl chloride, were unreactive in our case, which might arise from more difficult oxidative addition of C−Cl bonds.

We further examined carboxylation of other unactivated aryl (pseudo)halides (Fig. 2). Besides simple phenyl bromide **3a**, a broad range of *para*- and *meta*-substituted aryl bromides (**3b**–**3m**) reacted well with CO_2_ to afford the desired carboxylic acids in good yields. Interestingly, the sterically hindered aryl bromide **3n** bearing *ortho* phenyl group could also undergo the carboxylation in moderate efficiency. Moreover, the carboxylation of di-substituted phenyl (**3o**, **3p**) and fused (hetero)aryl bromides (**3r**–**3t**) was also smooth. Deactivated aryl bromides (**3c**, **3e**, **3j**, **3k**, **3p**–**3t**) bearing EDGs were also reactive in this process to give desired carboxylic acids in good yields. A set of functional groups, such as ether (**3c**, **3d**, **3j**, **3k**, **3p**), thioether (**3e**), hydroxyl (**3f**), fluoro (**3g**), ester (**3h**), alkene (**3l**), acetal (**3m**) and heterocycles (**3r**–**3t**), were well tolerated. Besides aryl bromides, aryl iodides (**3u**–**3w**) also underwent the carboxylation smoothly. Moreover, aryl sulfonates (**3x**–**3ab**), such as a mesylate, tosylates, and triflates, which are easily available from phenols, also showed high reactivity and selectivity in this reaction.

**Fig. 3:**
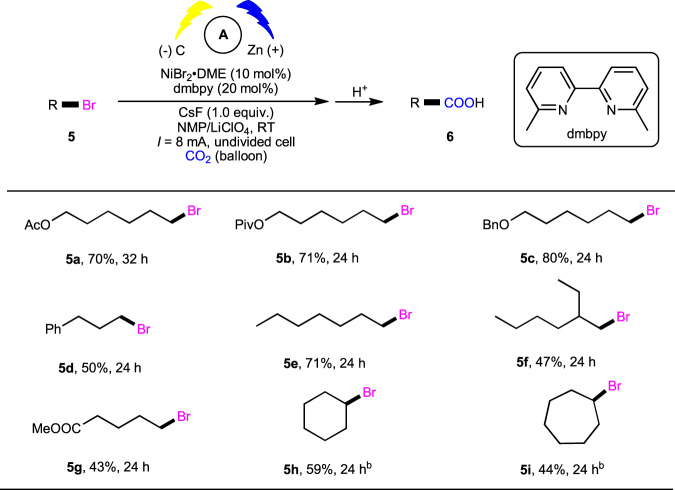
Scope of alkyl bromides. ^a^Reaction conditions, see more details in “Supplementary information”. ^b^I = 4 mA.

Furthermore, we investigated more challenging carboxylation of unactivated alkyl bromides to give important alkyl carboxylic acids. To our delight, a range of primary alkyl bromides (**5a**–**5g**) could afford the corresponding carboxylic acids in moderate to good yields with good functional group tolerance under slightly modified conditions (Fig. 3). Moreover, unactivated cyclic alkyl bromides (**5h** and **5i**) could also take part in this reaction to generate carboxylic acids with synthetically useful yields, although these substrates were considered as challenging coupling partners in carboxylation events^45,46^.

### Exploration of the electrochemical carboxylation without sacrificial anode

**Fig. 4:**
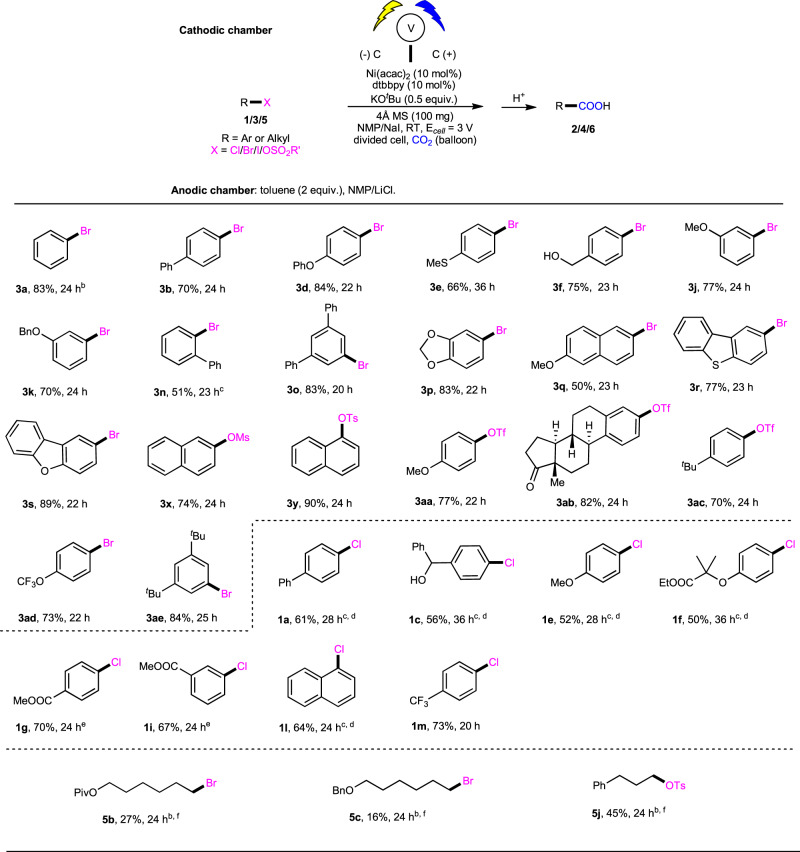
Scope of organo(pseudo) halides in non-sacrificial electrode manner. ^a^Reaction conditions, see more details in Table S2 in “Supplementary information”. ^b^0.5 mmol scale. ^c^60 °C. ^d^NiI_2_ (10 mol%), dtbbpy (10 mol%), DMAP (20 mol%), MgBr_2_ (1.5 equiv). Anodic chamber: Et_3_N (0.6 mmol), 3 Å MS (100 mg), NMP/NaI (0.2 M). ^e^DMAP (20 mol%) was added. ^f^NiBr_2_•DME (10 mol%), dmbby (20 mol%), CsF (1.0 quiv.), NMP/LiClO_4_ (0.2 M), RT. Anodic chamber: Et_3_N (0.6 mmol), NMP/LiClO_4_ (0.2 M). MS = molecule sieve.

Although electrocatalytic carboxylation of organo (pseudo) halides shows advantage to those with highly reactive metallic powders by using user-friendly and stable anodes, it would be more appealing and practical to achieve such carboxylation without using sacrificial electrodes^52–56^. Therefore, we further challenged us with development of an active electrocatalytic system with paired cells. After systematic investigation (see Supplementary Table 2), we were delighted to realize the catalytic electrochemical carboxylation of aryl bromide **3b** with CO_2_ avoiding the use of sacrificial electrodes in 70% isolated yield. Control experiments verified that the pair anodic oxidation was essential, and the electro-oxidative chlorination of toluene^61^ matched well with the desired Ni-catalyzed reductive carboxylation. Other common anodic oxidations, including the electro-oxidation of triethylamine and triethanolamine (Supplementary Table 2, entries 8–9) gave lower yields. Using this system, we found that a variety of unactivated aryl(hetero) bromides, tosylates, mesylates, and triflates worked well to give the desired carboxylic acids in satisfying yields (Fig. 4, **3a**–**3ae** (selective examples)). Unactivated aryl chlorides (**1a**–**1m**, selective examples) also took part in this reaction well under modified conditions with the paired oxidation of triethylamine. More challenging alkyl bromides/sulfonates (**5b**, **5c**, **5j**) were also investigated, however, low reactivity was observed.

### Mechanistic studies

**Fig. 5:**
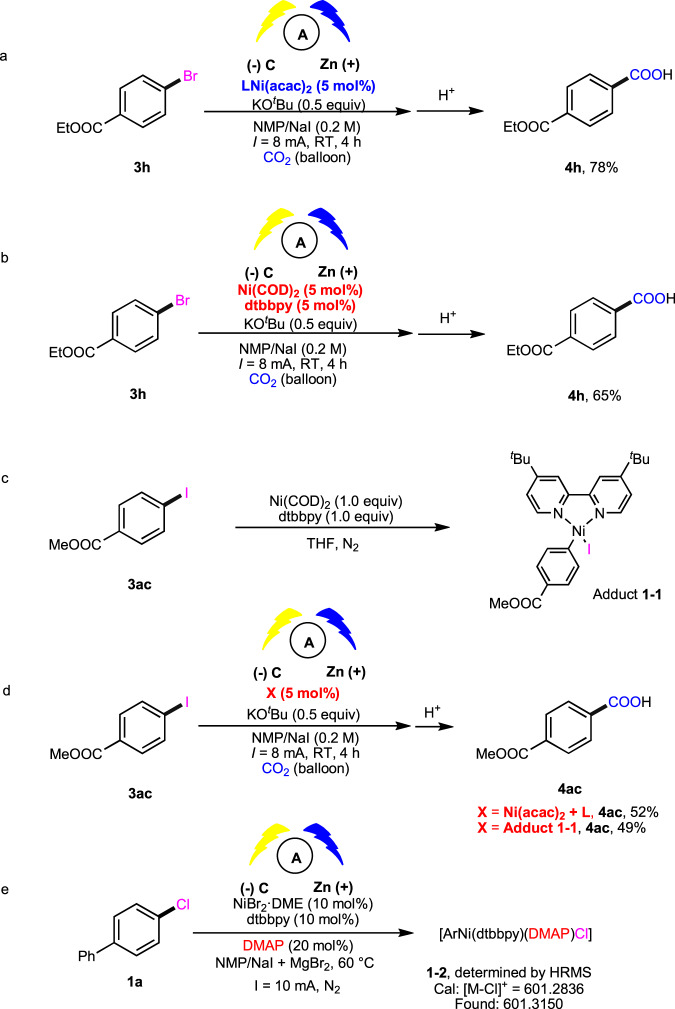
Control experiments. **a** Investigation of the effect of nickel complex LNi(acac)_2_. **b** Investigation of the effect of Ni(0) catalyst. **c** Preparation of oxidative addition adduct **1-1**. **d** Investigation of the effect of adduct **1-1**. **e** HRMS detection of oxidation addition adduct. L = dtbbpy. DMAP = *N, N*-dimethyl-4-aminopyridine. HRMS = High resolution mas spectrometry.

To gain more insight into the reaction mechanism, we first applied the synthesized complex LNi(acac)_2_ as pre-catalyst in the carboxylation of **3** **h** (Fig. 5a), which provided **4** **h** in a similar yield to that of standard reaction and higher than that obtained with Ni(COD)_2_ and dtbbpy (Fig. 5b). Moreover, we carried out the stoichiometric reaction of **3ac** with Ni-catalyst to generate an oxidative addition adduct **1-1** (Fig. 5c), which showed similar catalytic activity (Fig. 5d), indicating that **1-1** might be the key intermediate in the catalytic cycle. In addition, the complex **1-2** [ArNi(dtbbpy)(DMAP)Cl] was detected in the catalytic reaction (Fig. 5e).

**Fig. 6:**
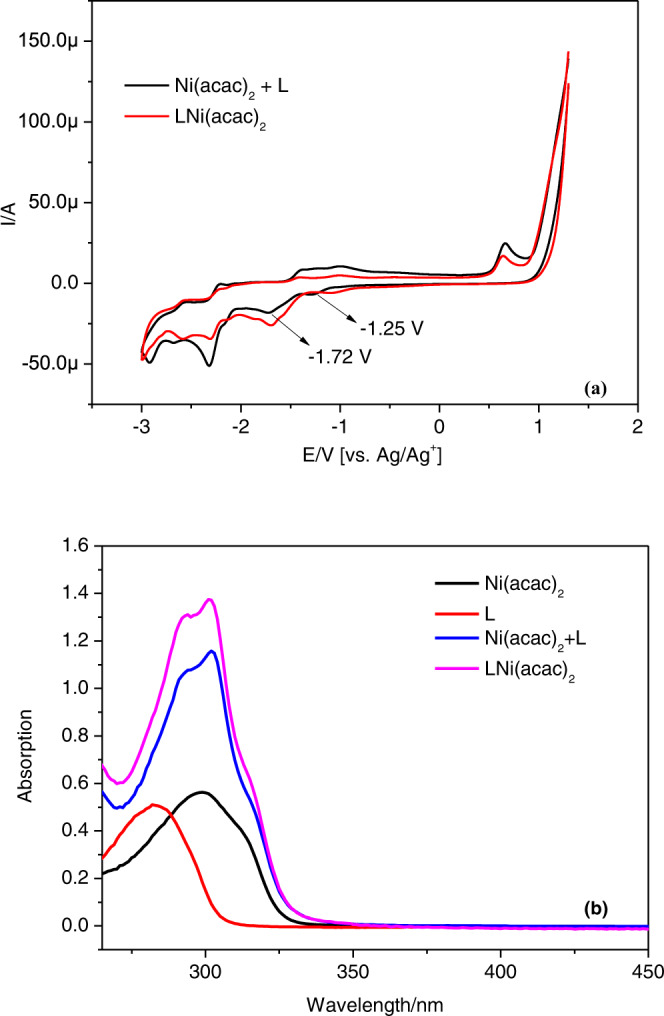
Electrochemical and spectroscopic investigations. **a** CV tests of [Ni(acac)_2_ + L] and LNi(acac)_2_. Testing conditions: working electrode, glassy carbon electrode; counter electrode, Pt wire; reference electrode, Ag/AgNO_3_ electrode (10 mM AgNO_3_ in CH_3_CN). Scan rate: 100 mV/s. Solvent: NMP/^*n*^Bu_4_NPF_6_ (0.1 M). Samples were tested under N_2_ atmosphere. Ni(acac)_2_, 3 mM; L, dtbbpy, 3 mM, LNi(acac)_2_, 3 mM. **b** UV-vis spectra of Ni(acac)_2_, 3 × 10^−5 ^M; L, 3 × 10^−5 ^M; [Ni(acac)_2_ + L], 3 × 10^−5 ^M + 3 × 10^−5 ^M and LNi(acac)_2_, 3 × 10^−5 ^M. NMP is chosen as the testing solvent. L = dtbbpy, 4,4′-di-tert-butyl-2,2′-bipyridine; NMP = 1-methyl-2-pyrrolidinone.

Furthermore, we also tested electrochemical analysis and UV-vis spectroscopy (Fig. 6). We found that both the cyclic voltammogram (CV) and absorption spectra of LNi(acac)_2_ were almost consistent with the mixture of Ni(acac)_2_ and dtbbpy in NMP solution (Fig. 6a, b), suggesting that pre-catalyst LNi(acac)_2_ was formed in situ by mixing Ni(acac)_2_ and dtbbpy by a ratio of 1:1 in NMP solution. All these results indicated that the coordination occurred between nickel catalyst and ligand. Besides, such nickel catalytic systems had two irreversible reductive peaks in the CV tests (Fig. 6a), which indicated that the nickel catalysts could be reduced into Ni(I) and Ni(0) species on the cathodic surface.

**Fig. 7:**
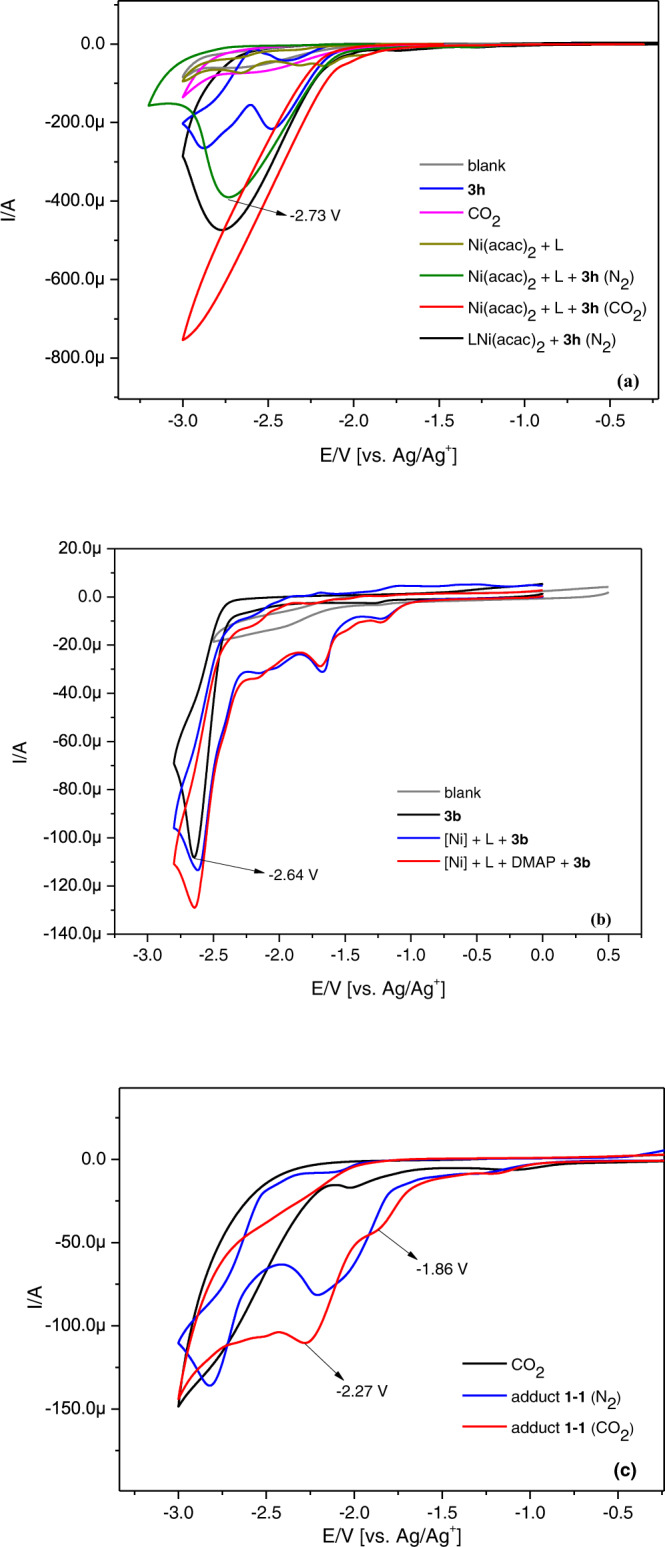
Performance of nickel catalysts in CV tests. Testing conditions: working electrode, glassy carbon electrode; counter electrode, Pt wire; reference electrode, Ag/AgNO_3_ electrode (10 mM AgNO_3_ in CH_3_CN). Scan rate: 100 mV/s. Solvent: NMP/^*n*^Bu_4_NPF_6_ (0.1 M). Samples were tested under N_2_ atmosphere except some cases under CO_2_ atmosphere. **a** CV tests of [Ni(acac)_2_ + dtbbpy] and aryl bromide **3** **h**. Ni(acac)_2_, 3 mM; L, dtbbpy, 3 mM; LNi(acac)_2_, 3 mM; **3** **h**, ethyl 4-bromobenzoate, 60 mM; CO_2_ (bubbled in the solution for 10 min). **b** CV tests for the role of DMAP. [Ni], NiBr_2_(DME), 3 mM; L, dtbbpy, 3 mM; DMAP, 3 mM, **3b**, 4-bromophenyl, 5 mM. **c** CV tests of adduct **1-1**. adduct **1-1**, 5 mM; CO_2_ (bubbled in the solution for 10 min). L, dtbbpy, 4,4′-di-tert-butyl-2,2′-bipyridine; DMAP, 4-dimethylaminopyridine.

We continued to conduct CV tests for Ni(acac)_2_ and dtbbpy in the presence of **3** **h** (Fig. 7a) and found a steep reductive peak at −2.73 V (E_p_), which indicated a typical catalytic current. Similarly strong catalytic current could also be observed when LNi(acac)_2_ was used in place of Ni(acac)_2_ and dtbbpy. Since the pre-formed nickel complex could be reduced into its Ni(0) form according to cyclic voltammetric results, we inferred that the responsive catalytic current was attributed to oxidative addition between **3** **h** and Ni(0) complex. After the introduction of CO_2_, the reductive peak current was further increased, indicating the carboxylation reaction occurred. Besides, the deactivated aryl bromide **3b**, which is reduced at −2.64 V (E_p_), showed the rise of its peak current in the Ni-catalytic system along with DMAP (Fig. 7b), suggesting that DMAP improved the Ni-catalytic efficiency in activating inert aryl bromide to reaction with nickel catalyst. CV test of adduct **1–1** was also investigated in NMP solution to show two irreversible reductive peaks, which might correspond to the reduction of adduct **1–1** at the cathodic surface (Fig. 7c)^62^. Furthermore, two new reductive peaks at −1.86 V (E_p_) and −2.27 V (E_p_) were emerged when adduct **1–1** was present in the CO_2_ atmosphere, which might indicate that oxidative addition complex **1–1** was reduced into its Ni(I) species, which then underwent carboxylation with CO_2_ and was further reduced into Ni(0) species^63^. All of the results indicated that adduct **1–1** was generated during the electrolytic process and then reduced to Ni(I) species which underwent carboxylation with CO_2_.

**Fig. 8:**
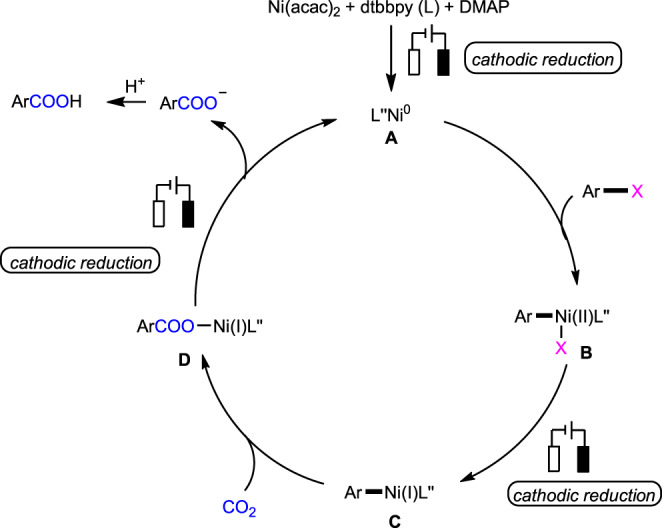
Proposed mechanistic cycle. Proposed reaction pathway is started up via the electro-generated Ni(0) species.

Based on these results and the previous work^15–20,31–38,64^, a possible catalytic cycle was proposed (Fig. 8). First, the L″Ni(0) species **A** is generated from the complex Ni(acac)_2_ in the presence of bipyridine ligand and DMAP on the cathode. Then the oxidative addition between **A** and the aryl halide occurred to yield the adduct **B**, which would be further reduced to Ni(I) species **C** by cathode. Then, **C** reacted with CO_2_ to give the nickel carboxylate intermediate **D**. Following ligand exchange and reduction of nickel carboxylate complex regenerated the active Ni(0)-catalyst **A** and afforded carboxylate, which would undergo protonation to give the desired product.

## Discussion

In this work, we have developed a general and practical electro-reductive Ni-catalytic system, realizing the electrocatalytic carboxylation of challenging, unactivated aryl chlorides and alkyl bromides with CO_2_. Unactivated aryl bromides, iodides, and sulfonates can also undergo such an electrocatalytic carboxylation smoothly. The use of DMAP as additional ligand and MgBr_2_ as Lewis acid improved the efficiency for the electrocarboxylation of unactivated aryl halides. Notably, we also realize the catalytic electrochemical carboxylation of aryl (pseudo)halides with CO_2_ avoiding the use of sacrificial electrodes. This protocol is operationally simple and robust with mild conditions, undivided cell, safe and cheap electrodes, good functional group tolerance, and broad substrate scope. Mechanistic investigations indicate that the reaction might proceed via oxidative addition of aryl halides to L_n_Ni(0), further reduction to the Ni(I) species, and following carboxylation with CO_2_. More application of such catalytic system in reductive couplings is underway in our laboratory.

## Methods

### Synthesis of 1a–1f, 1i–1l, 3a–3c, 3e, 3i–3l, 3p, 3s, and 3aa

In a 50 mL three-neck flask equipped with a carbon felt cathode and a Zn plate, aryl halides (0.3 mmol), NiBr_2_•DME (0.03 mmol, 10 mol%), 4,4′-di-tert-butyl-2,2′-bipyridine (0.03 mmol, 10 mol%), DMAP (0.06 mmol, 20 mol%), KO^*t*^Bu (0.15 mmol, 0.5 equiv), anhydrous MgBr_2_ (0.45 mmol, 1.5 equiv), NaI (1.2 mmol) were loaded in the glove box. Then the mixture was taken out of the box, degassed under vacuum, and back-filled with CO_2_ gas for five times (each time lasted for 1 min). After that, anhydrous NMP (6 mL) was injected into the flask via a syringe and dissolved the mixture under the strong stirring until the mixture becoming transparent. Then two electrodes were submerged into the solution and conducted under 60 °C. Constant current (10 mA) was passed until the starting material was consumed. After electrolysis, the mixture was acidized by 2 N HCl solution (20 mL) and extracted by EtOAc for four times (30 mL × 4). The combined organic layers were washed with water (20 mL × 2) and brine (20 mL) and concentrated in vacuo. Then the residue was purified by silica gel column chromatography by using the petroleum ether/EtOAc to give out the carboxylic acid.

### Synthesis of 3d, 3f–3h, 3m–3o, 3q–3r, 3t–3z and 3ab

In a 50 mL three-neck flask equipped with a carbon felt cathode and a Zn plate, aryl halides (0.3 mmol), Ni(acac)_2_ (0.015 mmol, 5 mol%), 4,4′-di-tert-butyl-2,2′-bipyridine (0.015 mmol, 5 mol%), KO^*t*^Bu (0.15 mmol, 0.5 equiv), NaI (1.2 mmol) were loaded in the glove box. Then the mixture was taken out of the box, degassed under vacuum and back-filled with CO_2_ gas for five times (each time lasted for 1 min). After that, anhydrous NMP (6 mL) was injected into the flask via a syringe and dissolved the mixture under the strong stirring until the mixture becoming transparent. Then two electrodes were submerged into the solution and conducted under room temperature or 50 °C. Constant current (8 mA) was passed until the starting material was consumed. After electrolysis, the mixture was acidized by 2 N HCl solution (20 mL) and extracted by EtOAc for four times (30 mL × 4). The combined organic layers were washed with water (20 mL × 2) and brine (20 mL) and concentrated in vacuo. Then the residue was purified by silica gel column chromatography by using the petroleum ether/EtOAc to give out the carboxylic acid.

### Synthesis of 5a–5i

In a 50 mL three-neck flask equipped with a carbon felt cathode and a Zn plate, alkyl bromides (0.5 mmol), NiBr_2_•DME (0.05 mmol, 10 mol%), 6,6′-di-methyl-2,2′-bipyridine (0.1 mmol, 20 mol%), CsF (0.5 mmol, 1.0 equiv), LiClO_4_ (1.2 mmol) were loaded in the glove box. Then the mixture was taken out of the box, degassed under vacuum, and back-filled with CO_2_ gas for five times (each time lasted for 1 min). After that, anhydrous NMP (6 mL) was injected into the flask via a syringe and dissolved the mixture under the strong stirring until the mixture becoming transparent. Then two electrodes were submerged into the solution and conducted under room temperature. Constant current (4 or 8 mA) was passed until the starting material was consumed. After electrolysis, the mixture was acidized by 2 N HCl solution (20 mL) and extracted by EtOAc for four times (30 mL × 4). The combined organic layers were washed with water (20 mL × 2) and brine (20 mL) and concentrated in vacuo. Then the residue was purified by silica gel column chromatography by using the petroleum ether/EtOAc to give out the carboxylic acid.

### Synthesis of 3a–3b, 3d–3f, 3j–3k, 3n–3s, 3x–3y, and 3aa–3ae (non-sacrificial anode manner)

In an H-type divided cell equipped with a carbon felt cathode and a carbon felt anode in each chamber, aryl bromides (0.3 mmol), Ni(acac)_2_ (0.03 mmol, 10 mol%), 4,4′-di-tert-butyl-2,2′-bipyridine (0.03 mmol, 10 mol%), KO^*t*^Bu (0.15 mmol, 0.5 equiv), 4 Å molecular sieve (100 mg), NaI (1.2 mmol) were loaded in the cathodic chamber. In the anodic chamber, LiCl (1.8 mmol) was added. Then the reaction vial was taken out of the box, degassed under vacuum, and back-filled with CO_2_ gas for five times (each time lasted for 1 min). After that, anhydrous NMP (6 mL) was injected into each chamber via a syringe and dissolved the mixture under the strong stirring until the solution becoming transparent. Then dry toluene (0.6 mmol) was injected into anodic chamber via syringe. After that, two electrodes were submerged into the solution. Constant cell voltage (3 V) was set to the reaction until the starting material was consumed at room temperature. After electrolysis, the mixture of both chambers was acidized by 2 N HCl solution (20 mL) and extracted by EtOAc for four times (30 mL × 4). The combined organic layers were washed with water (20 mL × 2) and saturated NH_4_Cl solution (20 mL) and concentrated in vacuo. Then the residue was purified by silica gel column chromatography by using petroleum ether/EtOAc to give out the carboxylic acids. Further details may be found in the Supplemental Information.

### Synthesis of 1a, 1c, 1e–1g, 1i, and 1l–1m (non-sacrificial anode manner)

In a H-type divided cell equipped with a carbon felt cathode and a carbon felt anode in each chamber, aryl bromides (0.3 mmol), NiI_2_ (0.03 mmol, 10 mol%), 4,4′-di-tert-butyl-2,2′-bipyridine (0.03 mmol, 10 mol%), DMAP (0.06 mmol, 20 mol%), KO^*t*^Bu (0.15 mmol, 0.5 equiv), NaI (1.2 mmol) were loaded in the cathodic chamber. In the anodic chamber, NaI (1.2 mmol) was added. Then the reaction vial was taken out of the box, degassed under vacuum, and back-filled with CO_2_ gas for five times (each time lasted for 1 min). After that, anhydrous NMP (6 mL) was injected into each chamber via a syringe and dissolved the mixture under the strong stirring until the solution becoming transparent. Then dry Et_3_N (0.6 mmol) was injected into anodic chamber via syringe. After that, two electrodes were submerged into the solution. Constant cell voltage (3 V) was set to the reaction until the starting material was consumed at room temperature. After electrolysis, the mixture of both chambers was acidized by 2 N HCl solution (20 mL) and extracted by EtOAc for four times (30 mL × 4). The combined organic layers were washed with water (20 mL × 2) and saturated NH_4_Cl solution (20 mL) and concentrated in vacuo. Then the residue was purified by silica gel column chromatography by using petroleum ether/EtOAc to give out the carboxylic acids. Further details may be found in the Supplemental Information.

### Synthesis of 5b, 5c, and 5j (non-sacrificial anode manner)

In a H-type divided cell equipped with a carbon felt cathode and a carbon felt anode in each chamber, alkyl halide (0.5 mmol), NiBr_2_•DME (0.05 mmol, 10 mol%), 6,6′-di-methyl-2,2′-bipyridine (0.1 mmol, 20 mol%), CsF (0.5 mmol, 1.0 equiv), LiClO_4_ (1.2 mmol) were loaded in the cathodic chamber. In the anodic chamber, LiClO_4_ (1.2 mmol) was added. Then the reaction vial was taken out of the box, degassed under vacuum, and back-filled with CO_2_ gas for five times (each time lasted for 1 min). After that, anhydrous NMP (6 mL) was injected into each chamber via a syringe and dissolved the mixture under the strong stirring until the solution becoming transparent. Then dry Et_3_N (0.6 mmol) was injected into anodic chamber via syringe. After that, two electrodes were submerged into the solution. Constant cell voltage (3 V) was set to the reaction until the starting material was consumed at room temperature. After electrolysis, the mixture of both chambers was acidized by 2 N HCl solution (20 mL) and extracted by EtOAc for four times (30 mL × 4). The combined organic layers were washed with water (20 mL × 2) and saturated NH_4_Cl solution (20 mL) and concentrated in vacuo. Then the residue was purified by silica gel column chromatography by using petroleum ether/EtOAc to give out the carboxylic acids. Further details may be found in the Supplemental Information.

## Supplementary information

Supplementary Information

## Data Availability

The authors declare that the data supporting the findings of this study are available within the article and its Supplemental Information files. Extra data are available from the author upon request.
