# Supplementary material for: Nickel-catalyzed electrochemical carboxylation of unactivated aryl and alkyl halides with CO2
Source: Nat Commun. 2021 Dec 6;12:7086. doi: 10.1038/s41467-021-27437-8 (PMC8648755; doi:10.1038/s41467-021-27437-8)
Supplement: Supplementary file 1 — Supplementary Information [file 41467_2021_27437_MOESM1_ESM.pdf]

Supplementary Information
for
Nickel-Catalyzed Electrochemical Carboxylation of Unactivated Aryl and Alkyl Halides with CO_2

Guo-Quan Sun,[1] Wei Zhang,[1] Li-Li Liao,[1] Li Li,[1] Zi-Hao Nie,[1] Jin-Gui Wu,[1] Zhen Zhang,[2] and Da-Gang Yu[1,3]*

[1]Key Laboratory of Green Chemistry & Technology of Ministry of Education, College of Chemistry, Sichuan University, Chengdu 610064

[2]College of Pharmacy and Biological Engineering, Chengdu University, Chengdu 610041

[3]Beijing National Laboratory for Molecular Sciences, Beijing 100190, P. R. China

***Corresponding author. Email:** dgyu@scu.edu.cn

Table of contents

Supplementary Methods
1. **General information 2**
2. **Experimental procedures 3**
3. **Details of the reaction set-ups 7**
4. **Reaction development and optimization 10**
5. **Cyclic voltammetry (CV) and UV-vis spectroscopy 12**
6. **Mechanistic studies 15**
7. **Consumed charge for substrates in the carboxylation 20**
8. **Characterization data for carboxylic acids 22**
9. **The spectrums of ^{1}H NMR, ^{19}F NMR and ^{13}C NMR 40**

Supplementary Reference 83

Supplementary Methods

1. General information

All starting materials and reagents are commercially available and were used directly without further purification. All of the reaction vials, stirring bars, carbon felt electrodes were heated to 120 °C under an oven for 2 hours and kept dry in a dryer before used. All the weighting procedures were performed in the glove box. Anhydrous solvents were purchased from *Acros Organics* and used as received. Molecular sieve (powder) should be heated at 500 °C for 3 hours under Muffle furnace and kept in the glove box. Commercially available chemicals were obtained from *Acros Organics*, *Aldrich Chemical Co*., *Alfa Aesar*, *Adamas Beta*, *ABCR and Energy* used as received unless otherwise stated, aryl sulfonates used in the research were provided from our lab[1]. Carbon dioxide (purity: 99.9%, industrial grade) was purchased from Chengdu Xuyuan Chemical Co., Ltd and used directly without further purification.

^{1}H, ^{13}C NMR and ^{19}F NMR spectra were recorded on a Brüker Advance 400 spectrometer (^{1}H: 400 MHz, ^{13}C: 101 MHz, ^{19}F: 376 MHz). Chemical shifts (δ) for ^{1}H, ^{13}C NMR and ^{19}F NMR spectra are given in ppm relative to TMS. The residual solvent signals were used as references for ^{1}H and ^{13}C NMR spectra and the chemical shifts converted to the TMS scale ($CDCl_3$: δ_H = 7.26 ppm, δ_C = 77.16 ppm; $(CD_3)_2CO$: δ_H = 2.05 ppm, δ_C = 29.84, 206.26 ppm; $(CD_3)_2SO$: δ_H = 2.50 ppm, δ_C = 39.52 ppm).

GC-MS was obtained using electron ionization (Agilent Technologies 7890B/GC-System and 5977A/MSD). TLC was performed using commercially prepared 100-400 mesh silica gel plates (GF254), and visualization was affected at 254 nm. High revolution mass spectra (HRMS) were recorded on a SHIMADZU LCMS-IT-TOF. ESI-MS were obtained on a Thermo LTQ mass spectrometer. Cyclic voltammetry tests were performed by using CHI 600E potentiostat (CH Instruments, Inc. USA) equipped with the conventional three electrode system with sweep rate of 100 mV•s^{-1}. The working electrode was a glassy carbon disk electrode (d = 0.3 cm). The auxiliary and reference electrode consisted of a Pt wire and an Ag/$AgNO_3$ (10 mM $AgNO_3$ in CH_3CN), respectively. Glassy carbon should be polished with a polishing cloth before each measurement. Maynuo DC Source meter (M8831, 30V/1A) was applied in the electrolysis. UV-vis spectrophotometer (UV-1800)

was applied to the measurement of absorption spectra. FT-IR spectrometer IRTracer-100 was used for characterization of Ni complex. Element analysis was performed by Organic Element Analyzer Flash EA 1112. UPLC experiments were performed on a Waters UPLC by using SHIMADZU-GL Wonda Cract ODS-2 column (5.0 µm, 4.6 × 150 mm).

The electrochemical carboxylation was carried out using a 50 mL three-neck flask (purchased from Beijing *Synthware Glass*) equipped with a carbon felt cathode (1 cm × 1 cm × 2 cm, purchased from Beijing Jinglong Graphite Plant, purity > 99%, dried at 120 ºC before use) and a Zinc plate anode (1 cm × 2 cm, purity > 98%); H-type divided cell was designed by ourselves.

2. Experimental procedures

2.1 General procedure for the electrochemical carboxylation of aryl chlorides and bromides

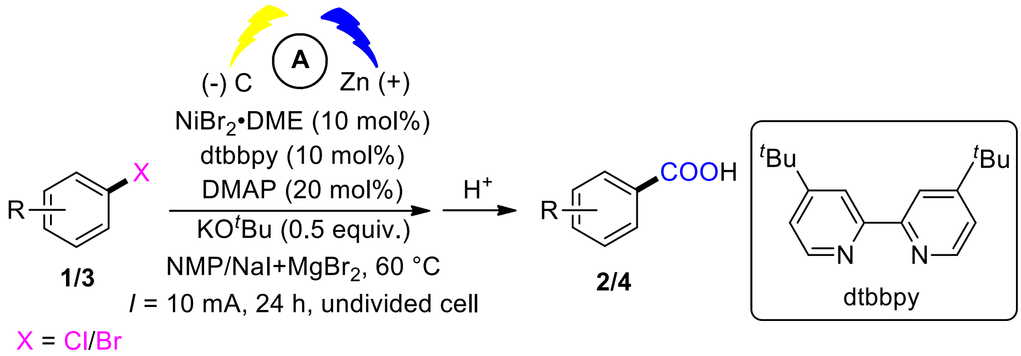

In a 50 mL three-neck flask equipped with a carbon felt cathode (1 cm × 1 cm × 2 cm) and a Zn plate (1 cm × 2 cm), aryl chloride/bromide **1/3** (0.3 mmol), $NiBr_2$•DME (10 mol%, 0.03 mmol, purchased from *Strem*), 4,4'-di-*tert*-butyl-2,2'-bipyridine (dtbbpy, 10 mol%, 0.03 mmol), *N,N*-dimethyl-4-aminopyridine (DMAP, 20 mol%, 0.06 mmol), KO^{t}Bu (0.15 mmol), anhydrous $MgBr_2$ (0.45 mmol), NaI (1.2 mmol) were loaded in the glove box. Then the mixture was taken out of the box, degassed under vacuum and back-filled with CO_2 gas for 5 times (each time lasted for 1 min). After that, 6 mL dry 1-methyl-2-pyrrolidinone (NMP, purchased from *Acros*, super dry grade) was injected into the flask via a syringe and dissolved the mixture under the strong stirring (1000 rp/min) until the mixture dissolves totally. Then two electrodes were submerged into the solution (the effective surface of cathode: approximately 1 cm × 1 cm × 1 cm; anode: approximately 1 cm × 1 cm). Constant current (10 mA) was passed until the starting material was consumed (monitored by Thin Layer Chromatography) at room temperature or 60 °C. After electrolysis, the mixture was acidized by 2 N

HCl solution (20 mL) and extracted by EtOAc for 4 times (30 mL × 4). [**Supplementary Note**: since carbon felt can absorb a significant amount of reaction solution, it was necessary to wash the electrode with EtOAc so as to avoid the residues staying in the electrode.] The combined organic layers were washed with water (20 mL × 2) and brine (20 mL) and concentrated *in vacuo*. Then the residue was purified by silica gel column chromatography by using the petroleum ether/EtOAc to give out the carboxylic acid **2**/**4**.

2.2 General procedure for the electrochemical carboxylation of aryl bromides, iodides and sulfonates

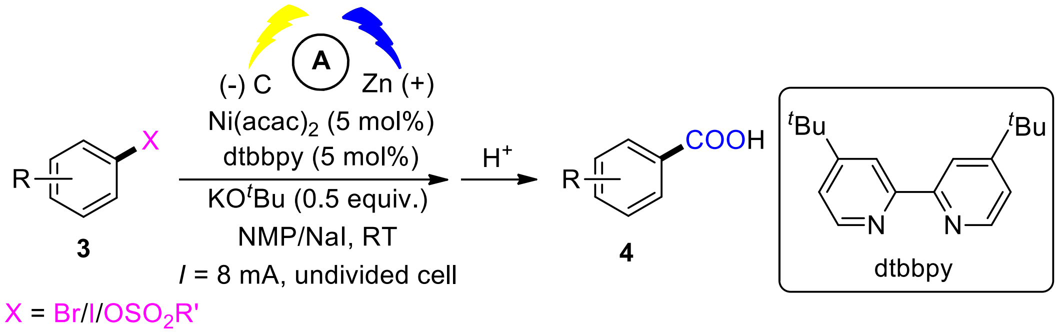

In a 50 mL three-neck flask equipped with a carbon felt cathode (1 cm × 1 cm × 2 cm) and a Zn plate (1 cm × 2 cm), aryl halides **3** (0.3 mmol), $Ni(acac)_2$ (5 mol%, 0.015 mmol, purchased from *Adamas*), 4,4'-di-*tert*-butyl-2,2'-bipyridine (dtbbpy, 5 mol%, 0.015 mmol), KO^{t}Bu (0.15 mmol), NaI (1.2 mmol) were loaded in the glove box. Then the mixture was taken out of the box, degassed under vacuum and back-filled with CO_2 gas for 5 times (each time lasted for 1 min). After that, 6 mL dry 1-methyl-2-pyrrolidinone (NMP, purchased from *Acros*, super dry grade) was injected into the flask via a syringe and dissolved the mixture under the strong stirring (1000 rp/min) until the mixture becoming transparent. Then two electrodes were submerged into the solution (the effective surface of cathode: approximately 1 cm × 1 cm × 1 cm; anode: approximately 1 cm × 1 cm). Constant current (8 mA) was passed until the starting material was consumed (monitored by Thin Layer Chromatography) at room temperature. After electrolysis, the mixture was acidized by 2 N HCl solution (20 mL) and extracted by EtOAc for 4 times (30 mL × 4). [**Supplementary Note**: since carbon felt can absorb a significant amount of reaction solution, thus it was necessary to wash the electrode with EtOAc so as to avoid the residues staying in the electrode.] The combined organic layers were washed with water (20 mL × 2) and brine (20 mL) and concentrated *in vacuo*. Then the residue was purified by silica gel

column chromatography by using the petroleum ether/EtOAc to give out the carboxylic acid **4**.

2.3 General procedure for the electrochemical carboxylation of aliphatic bromides

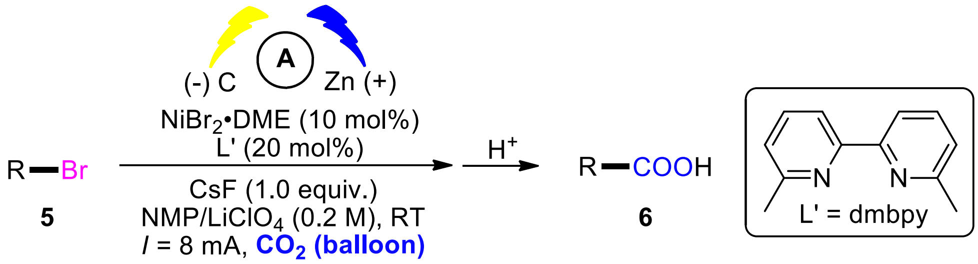

In a 50 mL three-neck flask equipped with a carbon felt cathode (1 cm × 1 cm × 2 cm) and a Zn plate (1 cm × 2 cm), alkyl bromide **5** (0.5 mmol), $NiBr_2$•DME (10 mol%, 0.05 mmol, purchased from *Aldrich*), 6,6'-di-methyl-2,2'-bipyridine (20 mol%, 0.1 mmol), anhydrous CsF (0.5 mmol), $LiClO_4$ (1.2 mmol) were loaded in the glove box. Then the mixture was taken out of the box, degassed under vacuum and back-filled with CO_2 gas for 5 times (each time lasted for 1 min). After that, 6 mL dry 1-Methyl-2-pyrrolidinone (NMP, purchased from *Acros*, super dry grade) was injected into the flask via a syringe and dissolved the mixture under the strong stirring (1000 rp/min) until the solution becoming transparent. Then two electrodes were submerged into the solution (the effective surface of cathode: approximately 1 cm × 1 cm × 1 cm; anode: approximately 1 cm × 1 cm). Constant current (8 mA) was passed until the starting material was consumed (monitored by Thin Layer Chromatography) at room temperature. After electrolysis, the mixture was acidized by 2 N HCl solution (20 mL) and extracted by EtOAc for 4 times (30 mL × 4). [**Supplementary Note**: since carbon felt can absorb a significant amount of reaction solution, thus it was necessary to wash the electrode with EtOAc so as to avoid the residues staying in the electrode.] The combined organic layers were washed with water (20 mL × 2) and brine (20 mL) and concentrated *in vacuo*. Then the residue was purified by silica gel column chromatography by using petroleum ether/EtOAc to give out the aliphatic carboxylic acid **6**.

2.4 General procedure for the electrochemical carboxylation of organo (pseudo)halides in non-sacrificial anode manner

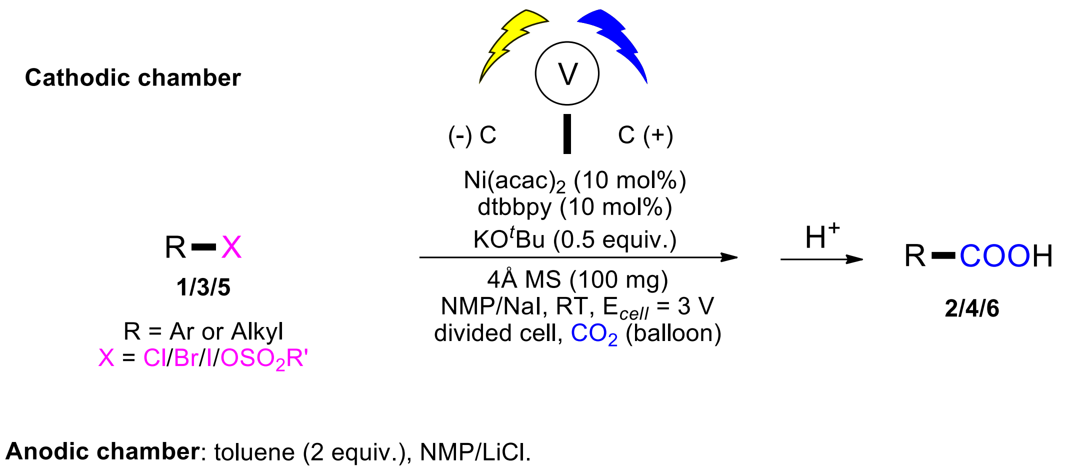

In a H-type divided cell equipped with a carbon felt cathode (1 cm × 1 cm × 2 cm, in cathodic chamber) and a carbon felt anode (1 cm × 1 cm × 2 cm, in anodic chamber), organo (pseudo)halides **1/3/5** (0.3 mmol in general, 0.5 mmol of alkyl bromides was used in the experiment), $Ni(acac)_2$ (10 mol%, 0.03 mmol, purchased from *Adamas Beta*), 4,4'-di-*tert*-butyl-2,2'-bipyridine (dtbbpy, 10 mol%, 0.03 mmol), KO^tBu (0.15 mmol), 4Å molecular sieve (100 mg), NaI (1.2 mmol) were loaded in the cathodic chamber [For the cases of aryl chlorides, NiI_2 (10 mol%, 0.03 mmol), dtbbpy (10 mol%, 0.03 mmol), DMAP (20 mol%, 0.06 mmol), $MgBr_2$ (0.45 mmol), NaI (1.2 mmol), 4Å molecular sieve (100 mg) were added in cathodic chamber; For the cases of alkyl bromides, $NiBr_2$•DME (10 mol%, 0.05 mmol), dmbby (20 mol%, 0.1 mmol), CsF (0.5 mmol), $LiClO_4$ (1.2 mmol) were added in cathodic chamber]. In the anodic chamber, LiCl (1.8 mmol) was added [For the cases of aryl chlorides, NaI (1.2 mmol) and 3Å molecular sieve (100 mg) were added in anodic chamber; For the cases of alkyl bromides, $LiClO_4$ (1.2 mmol) was added in anodic chamber]. Then the reaction vial was taken out of the box, degassed under vacuum and back-filled with CO_2 gas for 5 times (each time lasted for 1 min). After that, 6 mL dry 1-Methyl-2-pyrrolidinone (NMP, purchased from *Acros*, super dry grade) was injected into each chamber via a syringe and dissolved the mixture under the strong stirring (1000 rp/min) until the solution becoming transparent. Then dry toluene (0.6 mmol) was injected into anodic chamber via syringe [dry Et_3N (0.6 mmol) was used for the cases of aryl chlorides and alkyl bromides, Et_3N was dried with flake KOH overnight]. After that, two electrodes were submerged into the solution (the effective surface of both electrodes: approximately 1 cm × 1 cm × 1 cm). Constant cell voltage (3 V) was set to the reaction until the starting material was consumed (monitored by Thin Layer Chromatography) at room temperature. After electrolysis, the mixture of both chambers was acidized by 2 N HCl solution (20 mL) and extracted by EtOAc for 4 times (30 mL × 4).

[**Supplementary Note**: since carbon felt can absorb a significant amount of reaction solution, thus it was necessary to wash the electrode with EtOAc so as to avoid the residues staying in the electrode.] The combined organic layers were washed with water (20 mL × 2) and saturated NH_4Cl solution (20 mL) and concentrated *in vacuo*. Then the residue was purified by silica gel column chromatography by using petroleum ether/EtOAc to give out the carboxylic acids **2/4/6**.

3. Details of the reaction set-ups

3.1 Undivided cell setting

Carbon felt: 15 cm × 15 cm × 1 cm (purity > 99%); **Zinc plate:** 20 cm × 20 cm × 0.3 cm (purity > 98%)

Copper wire: d = 1 mm.

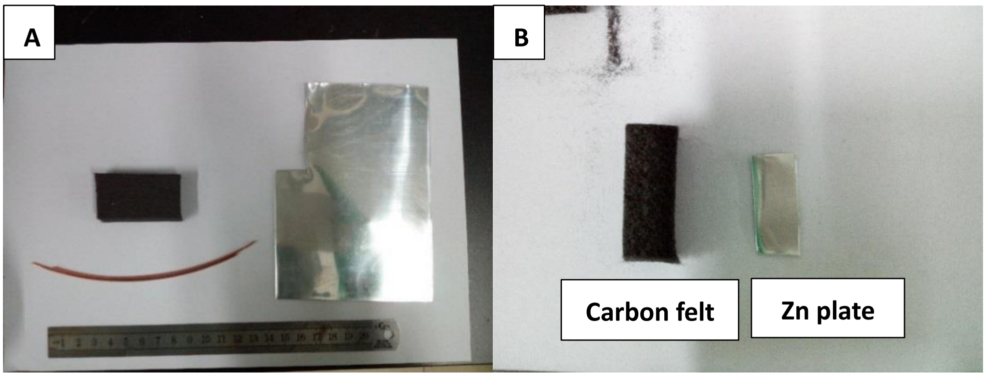

- Carbon felt was cut into a dice which was about 1 cm × 1 cm × 2 cm and Zn plate was cut into a 1 cm × 2 cm small part (See picture A and B).

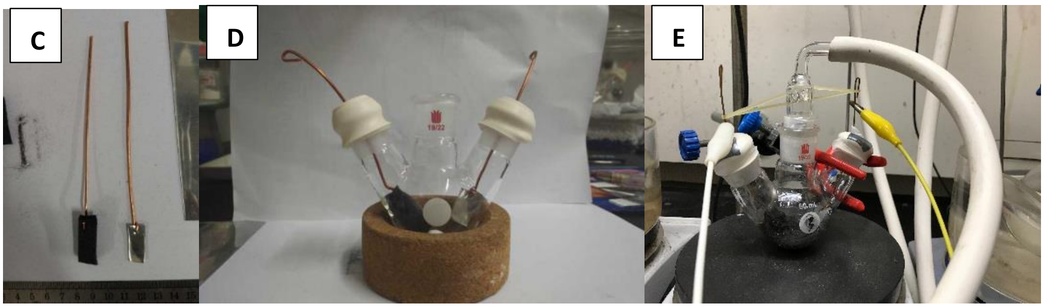

- The carbon felt and Zn plate were pierced through by a copper hook so as to stabilize the electrode material and make sure the electrode conductible (8~10 cm of copper wire was recommended) (C). Then the prepared electrodes were inserted through a pierced septum, which had a good gas tightness and avoided the CO_2 leakage, assembled on the left and right arm of the flask (50 mL

three-necked flask, D). It is noteworthy to mention that septum should be wrapped by the *Parafilm* to make sure the gas tightness.

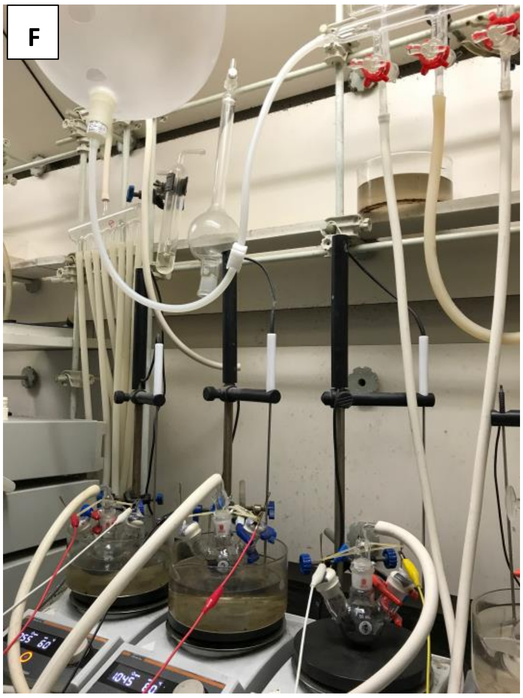

- The three-neck flask was connected with the rubber tube which was from the CO_2 Schlenk tube system on the joint outlet (See picture E & F), then displacing the CO_2 gas for 5 times (1 min per time). CO_2 airbag connected with the flask during electrolysis (see picture F).

3.2 Divided cell setting

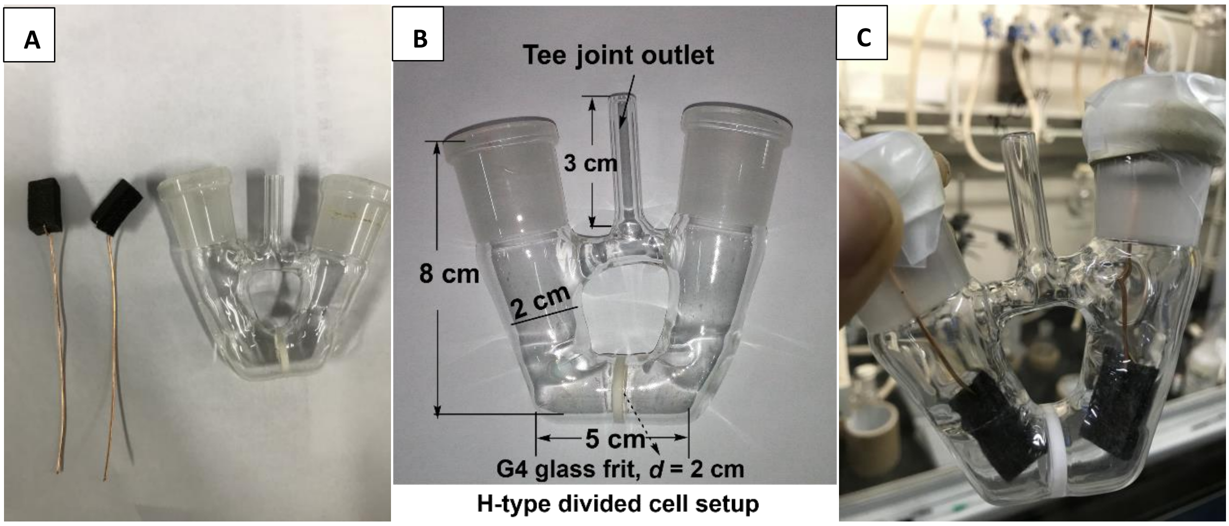

- The H-type divided cell was designed in our lab. The frit we use is G4 standard. Both carbon felt electrodes were inserted through a pierced septum and placed in each chamber (See picture A-C). It is noteworthy to mention that septum should be wrapped by the *Parafilm* to make sure the gas tightness.

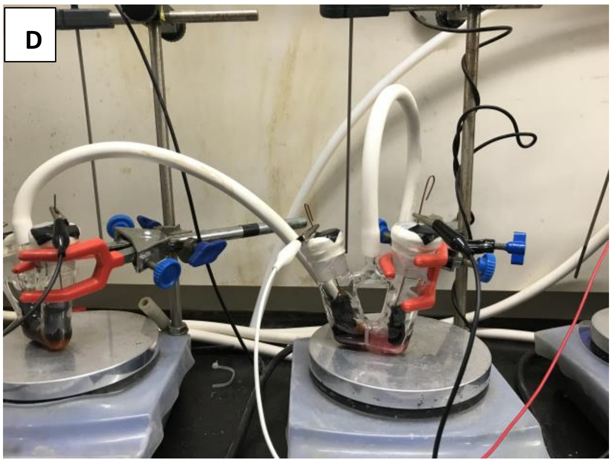

- The divided cell connected with the rubber tude which was from the CO_2 Schlenk tude system on the teen joint outlet (See picture D), then displacing the CO_2 gas for 5 times (1 min per time). CO_2 airbag connected with the flask during electrolysis.

4. Reaction development and optimization

Supplementary Table 1. Reaction development for electrochemical carboxylation of aryl chloride **1a**[a]

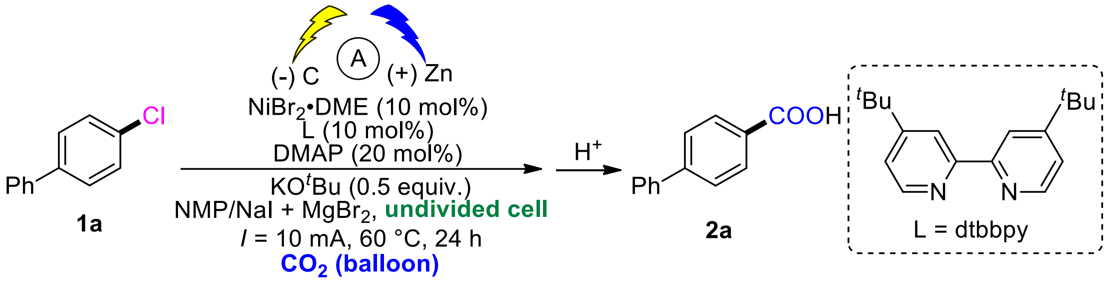

Entry	Variants from above conditions	Yields of **2a** (%)[b]
1	none	70 (70)
2	w/o DMAP	29
3	w/o dtbbpy	0
4	w/o $MgBr_2$	58
5	w/o $NiBr_2$•DME	0
6	w/o CO_2	0
7	w/o electricity	0
8	w/o KO*t*Bu	39
9	*n*Bu_4NPF_6 instead of NaI	38
10	DMF instead of NMP	24
11	5 mA instead of 10 mA	58
12	20 mA instead of 10 mA	12
13	Mg as anode	19

[a]Standard conditions: **1a** (0.3 mmol), $NiBr_2$•DME (10 mol%), dtbbpy (10 mol%), DMAP (20 mol%), KOtBu (0.5 equiv.), $MgBr_2$ (1.5 equiv.), NMP/NaI (0.2 M), CO_2 (balloon), 60 °C, constant current 10 mA, undivided cell, 24 h, carbon felt cathode, zinc anode. [b]Yields were determined by UPLC using 2-Naphthoic acid as the internal standard, along with isolated yield given in the parenthesis. w/o = without. DMF = dimethyl formamide, NMP = 1-methyl-2-pyrrolidone. DME = dimethoxylethane.

Supplementary Table 2. Reaction development of non-sacrificial anode manner[a]

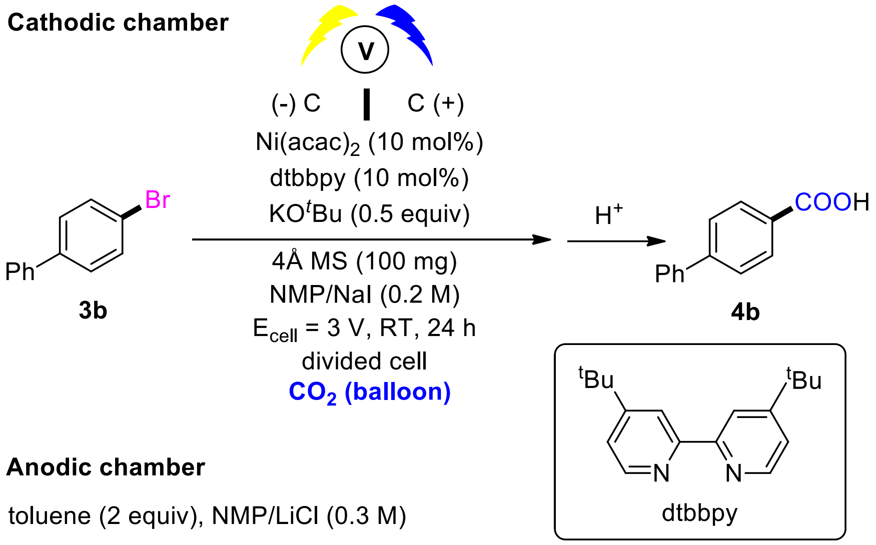

Entry	Variants from above conditions	Yield of **4b** (%)[b]
1	None	74 (70)
2	w/o $Ni(acac)_2$	0
3	w/o dtbbpy	0
4	w/o KO*t*Bu	23
5	w/o electricity	0
6	w/o CO_2	0
7	w/o 4Å MS	61
8	TEA oxidation as pair reaction	72
9	TEOA oxidation as pair reaction	29
10	E_{cell} = 2 V	22
11	E_{cell} = 5 V	15

[a]Cathodic chamber: aryl bromide **3b** (0.3 mmol), $Ni(acac)_2$ (10 mol%), dtbbpy (10 mol%), KO^{t}Bu (0.5 equiv), 4Å molecular sieve (100 mg), NMP/NaI (0.2 M). Anodic chamber: toluene (0.6 mmol), NMP/LiCl (0.3 M). CO_2 (balloon). Carbon felt was used as electrode material for both chambers, divided cell, constant cell voltage E_{cell} = 3 V, RT, 24 h. [b]Yields were determined by UPLC using 2-Naphthoic acid as the internal standard, along with isolated yield given in the parenthesis. w/o = without. $Ni(acac)_2$ = nickel (II) acetylacetonate. NMP = 1-methyl-2-pyrrolidone. MS = molecular sieve. TEA = triethylamine. TEOA = triethanolamine.

5. Cyclic voltammetry (CV) and UV-vis spectroscopy

5.1 CVs and UV-vis spectroscopy for nickel-catalytic system

All of the cyclic voltammetry experiments were conducted via the three-electrode system. The glassy carbon electrode was selected as working electrode; counter electrode was chosen as Pt wire and the reference electrode was $Ag/AgNO_3$ (10 mM $AgNO_3$ in CH_3CN). Scan rate was set at 100 mV/s. NMP/$^n Bu_4NPF_6$ (0.1 M) was chosen as the testing solution. Ferrocene as internal standard. E (Fc/Fc^+ vs $Ag/AgNO_3$) = 0.1 V. The scan direction is started from positive to negative. All of the samples should be bubbled with N_2 for 10 min before testing except the cases with CO_2. The UV-vis spectroscopic experiments were performed under N_2 atmosphere and NMP was chosen as the testing solvent.

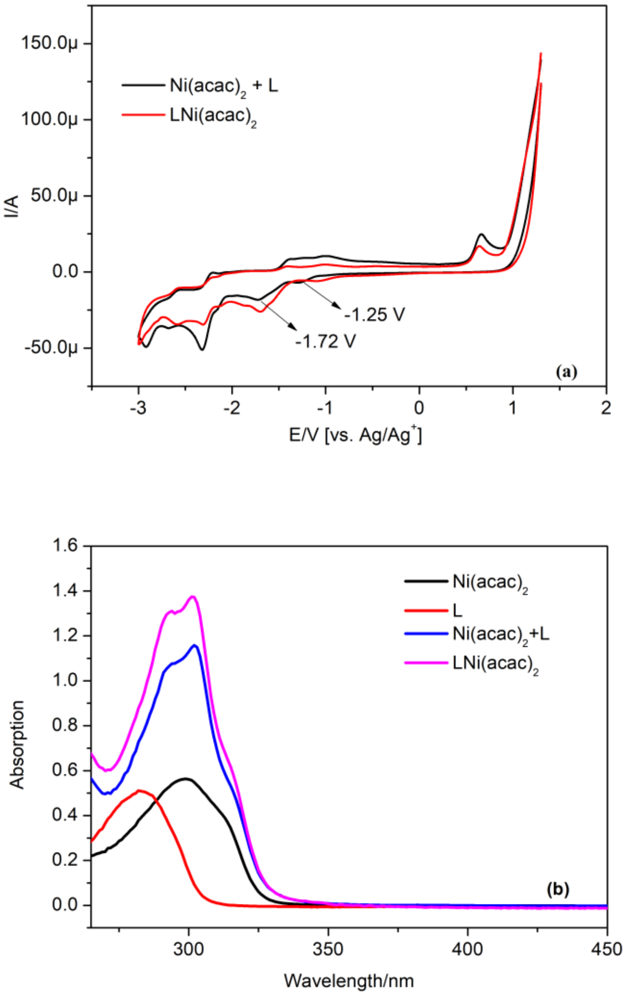

Supplementary Figure 1. (a) black line: $Ni(acac)_2$ (3 mM) + L (3 mM); red line: $LNi(acac)_2$ (3 mM); (b)

Spectroscopy of nickel-catalyzed system (dry NMP solution). Black line: $Ni(acac)_2$ (3×10^{-5} M); red line: L (3×10^{-5} M); blue line: $Ni(acac)_2$ (3×10^{-5} M) + L (3×10^{-5} M); pink line: $LNi(acac)_2$ (3×10^{-5} M). L = dtbbpy, 4,4'-di-*tert*-butyl-2,2'-bipyridine.

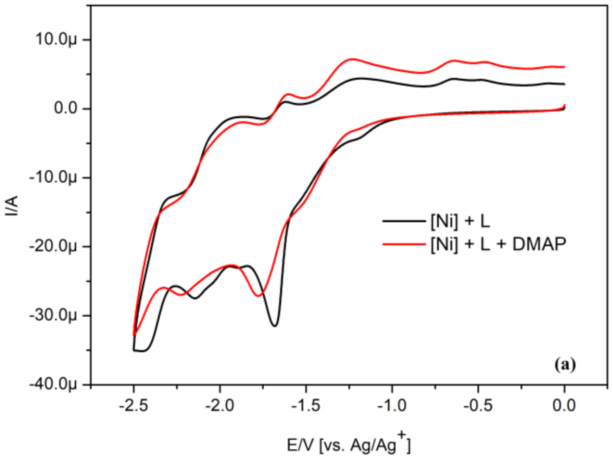

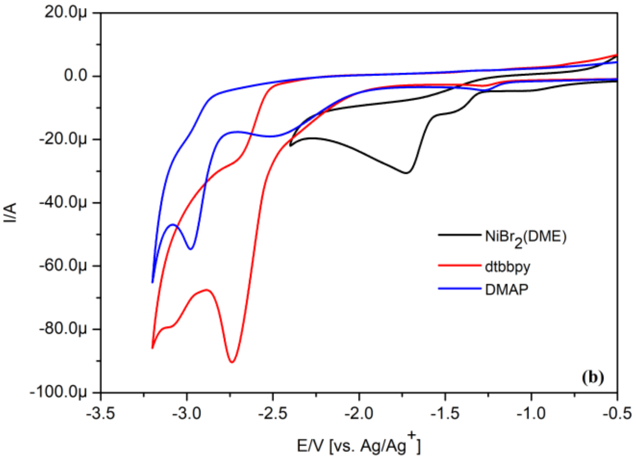

Supplementary Figure 2. CVs of $NiBr_2$(DME) + ligands system. a. [Ni]: $NiBr_2$(DME) (3 mM); L: dtbbpy, 4,4'-di-*tert*-butyl-2,2'-bipyridine (3 mM); DMAP, 4-dimethylaminopyridine (6 mM). b. CVs of each component. [Ni]: $NiBr_2$(DME) (3 mM); dtbbpy, 4,4'-di-*tert*-butyl-2,2'-bipyridine (3 mM); DMAP, 4-dimethylaminopyridine (3 mM).

Supplementary Note: Numerous reductive peaks are observed in this nickel catalyst system (see Supplementary Figure 2a). Also, CVs of the single component (see Supplementary Figure 2b) represents that nickel salt $NiBr_2$(DME) is easier to be reduced than bipyridine ligand and DMAP, the reductive peaks of nickel salt involve in the potential range of -1.2~-1.7 V. Although the CV of nickel catalyzed system is complex, we suppose that the reductive peaks which involve in the range of -1.2~-1.8 V in Supplementary Figure 2a might

be related with nickel reduction since the reduction of nickel salt $NiBr_2$(DME) reveals in the similar potential range (see Supplementary Figure 2b, black line).

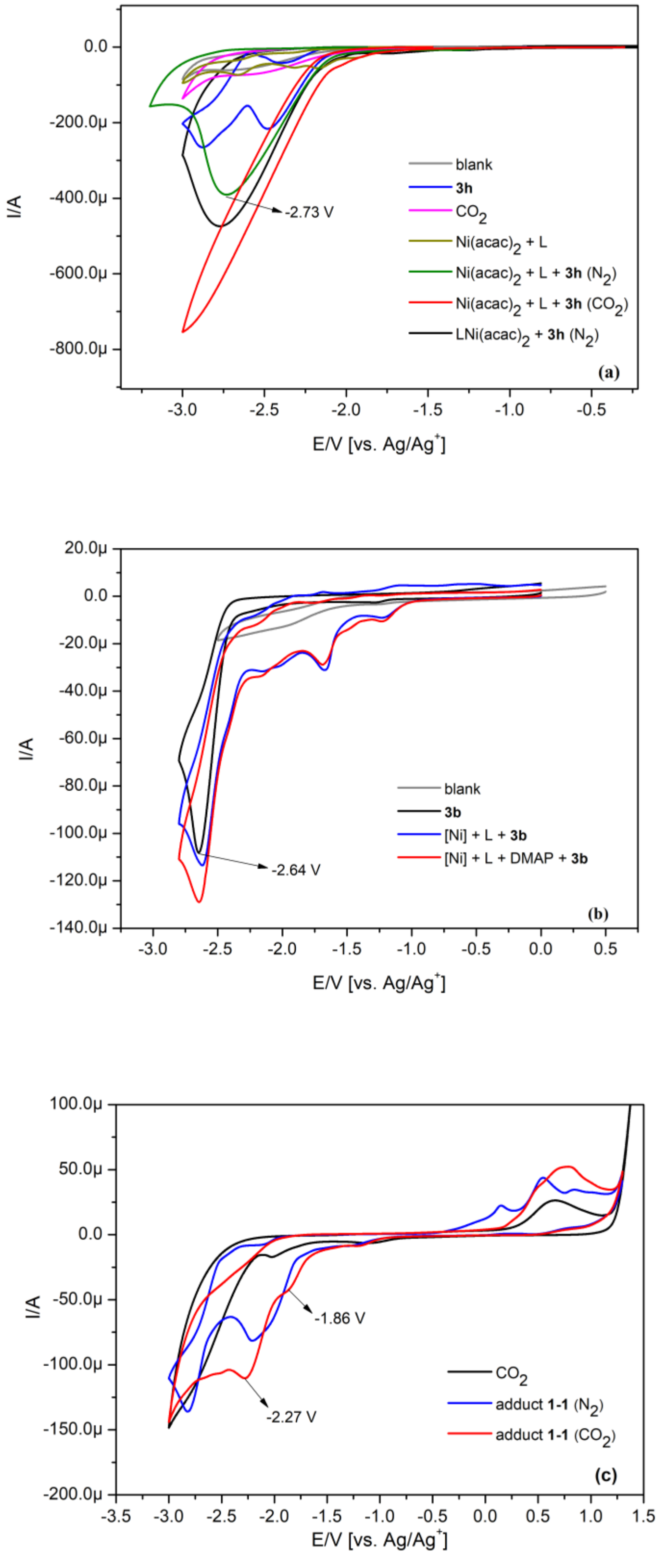

Supplementary Figure 3. (a) Catalytic behavior of aryl bromide **3h** in nickel-catalyzed system. Grey line: blank.; Blue line: **3h** (60 mM); pink line: CO_2 (bubbled in the solution for 10 minutes); brown yellow line: Ni(acac)$_2$ (3 mM) + L (3 mM); green line: Ni(acac)$_2$ (3 mM) + L (3 mM) + **3h** (60 mM); red line: Ni(acac)$_2$ (3 mM) + L (3 mM) + **3h** (60 mM) + CO_2; black line: LNi(acac)$_2$ (3 mM) + **3h** (60 mM). (b) Catalytic behavior of aryl bromide **3b** in nickel-catalyzed system. Black line: **3b** (5 mM); blue line: $NiBr_2$(DME) (3 mM) + L (3 mM) + **3b** (5 mM); red line: $NiBr_2$(DME) (3 mM) + L (3 mM) + DMAP (6 mM) + **3b** (5 mM). (c) Electrochemical behavior of adduct **1-1**. black line: CO_2 was bubbled into the NMP/^{n}Bu$_4$NPF$_6$ for 20 minutes. Blue line: adduct **1-1** (5 mM) in N_2 atmosphere. red line: adduct **1-1** (5 mM) in CO_2 atmosphere, the sample was prepared in glove box then bubbled CO_2 gas for 10 min. L = dtbbpy, 4,4'-di-*tert*-butyl-2,2'-bipyridine; DMAP, 4-dimethylaminopyridine.

Supplementary Note: adduct **1-1** is rather sensitive to air and the solvent NMP/^{n}Bu$_4$NPF$_6$ (0.1 M) should be prepared under N_2 atmosphere. After preparing the sample, the solution should be sealed in the specific CV testing cell for further experiments, the CV testing was conducted under N_2 atmosphere.

6. Mechanistic studies

6.1 Preparation of pre-catalyst LNi(acac)$_2$[2]

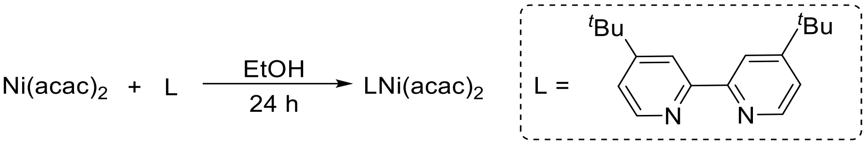

Ni(acac)$_2$ (1.8 mmol) was suspended into 10 mL dry EtOH and then a solution of 1.8 mmol of the ligand in 10 mL dry EtOH was added dropwise via a syringe (for about 10 mins) to a stirred solution of metal salt under N_2 at room temperature. After adding the entire solution, the whole mixture turned into a blue, transparent solution and the stirring lasted for 24 hours. At this time, light blue solid was precipitated, then filtered and washed first with two 4 mL portions of glacier EtOH and then with one 4 mL portion of dry Et_2O to give out the final product.

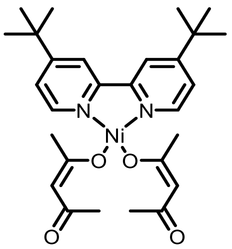

Pre-catalyst, light blue solid, 401.4 mg, 52%.

FT-IR (KBr, cm^{-1}): 3434.45, 2966.51, 1603.08, 1550.33, 1511.97, 1463.29, 1404.55, 1299.93, 1252.08, 1250.91, 1190.92, 1155.44, 1013.86, 915.49, 852.92, 746.49, 661.99, 607.48, 561.04, 416.55; **UV-vis** (3×10^{-5} M in dry NMP solution): 293 nm, 303 nm. **Elemental analysis.** Calcd (found) for $C_{28}H_{38}N_2NiO_4$: C, 64.02 (64.27); H, 7.29 (7.64); N, 5.33 (4.93). **HRMS (ESI+):** calculated m/z for [M-acac]$^+$: 425.1739, found: 425.1736. acac = acetylacetone.

6.2 Reaction of pre-catalyst LNi(acac)$_2$-catalyzed reaction of 3h with CO_2

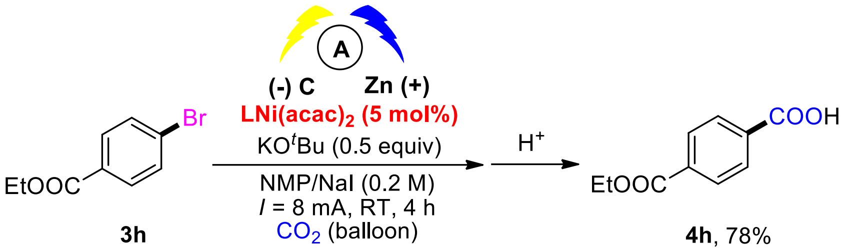

In a 50 mL three-neck flask equipped with a carbon felt cathode (1 cm × 1 cm × 2 cm) and a Zn plate (1 cm × 2 cm), aryl bromides **3h** (0.3 mmol), LNi(acac)$_2$ (0.015 mmol, L = dtbbpy), KO^{t}Bu (0.15 mmol), NaI (1 mmol) were loaded in the glove box. Then the mixture was taken out of the box, degassed under vacuum and back-filled with CO_2 gas for 5 times (each time lasted for 1 min). After that, 6 mL dry 1-methyl-2-pyrrolidinone (NMP) was injected into the flask via a syringe and dissolved the mixture under the strong stirring until the mixture becoming transparent. Then two electrodes were submerged into the solution (the effective surface of cathode: approximately 1 cm × 1 cm × 1 cm; anode: approximately 1 cm × 1 cm). Constant current (8 mA) was passed until the starting material was consumed for 4 hours at room temperature. After the electrolysis, the mixture was acidized by 2 N HCl solution (20 mL) and extracted by EtOAc for 4 times (30 mL × 4). The combined organic layers were washed with water (20 mL × 2) and brine (20 mL) and concentrated *in vacuo*. Then the residue was purified by silica gel column chromatography using petrolumn ether/EtOAc to give out the final product **4h** in 78% yield.

6.3 Reaction of Ni(COD)$_2$-catalyzed reaction of 3h with CO_2

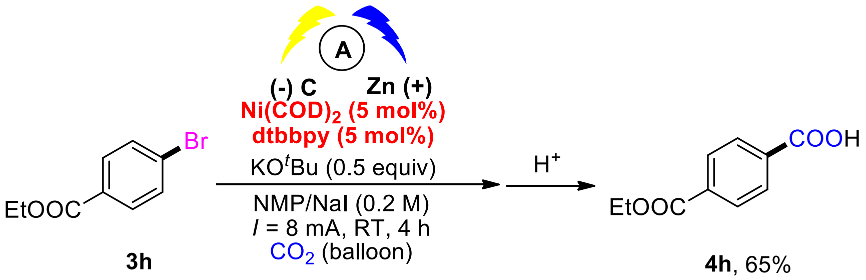

In a 50 mL three-neck flask equipped with a carbon felt cathode (1 cm × 1 cm × 2 cm) and a Zn plate (1 cm × 2 cm), aryl bromides **3h** (0.3 mmol), Ni(COD)$_2$ (0.015 mmol), dtbbpy (0.015 mmol), KO^{t}Bu (0.15 mmol), NaI (1 mmol) were loaded in the glove box. Then the mixture was taken out of the box,

degassed under vacuum and filled back the CO_2 gas for 5 times (each time lasted for 1 min). After that, 6 mL dry 1-methyl-2-pyrrolidinone (NMP) was injected into the flask via a syringe and dissolved the mixture under the strong stirring until the mixture becoming transparent. Then two electrodes were submerged into the solution (the effective surface of cathode: approximately 1 cm × 1 cm × 1 cm; anode: approximately 1 cm × 1 cm). Constant current (8 mA) was passed until the starting material was consumed for 4 hours at room temperature. After the electrolysis, the mixture was acidized by 2 N HCl solution (20 mL) and extracted by EtOAc for 4 times (30 mL × 4). The combined organic layers were washed with water (20 mL × 2) and brine (20 mL) and concentrated *in vacuo*. Then the residue was purified by silica gel column chromatography by using petrolumn ether/EtOAc to give out the final product **4h** in 65% yield.

6.4 Preparation of adduct 1-1[3]

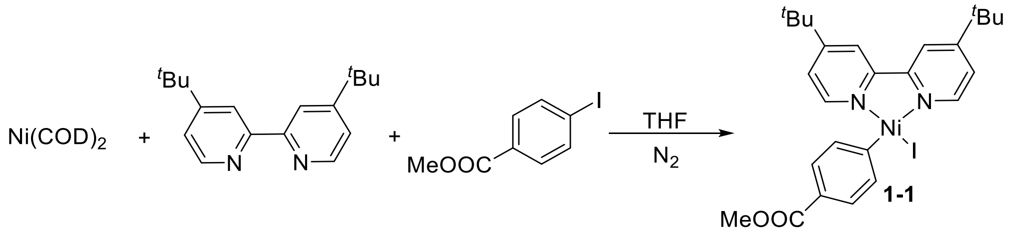

The oxidative addition complex **1-1** was prepared according to the literature. Under N_2 atmosphere, a suspension of $Ni(COD)_2$ (330.4 mg, 1.20 mmol, 100 mol %) in 8 mL of dry THF was stirred for 1 minutes in a 50 mL flame-dried Schlenk tube, at which point a solution of ligand dtbbpy (322.1 mg, 1.20 mmol, 100 mol %) in 8 mL of dry THF was added dropwise. The resulting mixture was allowed to stir overnight at ambient temperature. At this time, the color was changed into royal purple. Then a solution of methyl 4-iodobenzoate (314.4 mg, 1.20 mmol, 100 mol %) in 4 mL of dry THF was added via syringe. The resultant mixture was quickly changed into brownish-red and allowed to stir for another 1 hour. The solvent was removed carefully under vacuum, and the residue was filtrated with a fritted funnel, and washed with dry ether (3×20 mL) under N_2 atmosphere. The brownish red solid was collected, and further stirred in dry ether (50 mL) to dissolve aryl dimer. After filtration, and dried *in vacuo*, the brownish-red solid was obtained, which was stored in the glove box at -30 °C.

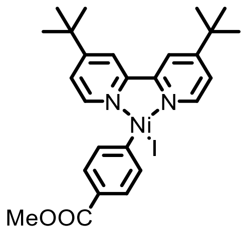

Adduct **1-1** was prepared according to the literature and could be obtained the brownish-red solid in about 70%. **^{1}H NMR (400 MHz, Acetone-d_6):** δ = 9.53 (s, 1H), 8.44 (s, 1H), 8.12 (s, 1H), 8.03 – 7.61 (m, 4H), 7.48 (s, 2H), 6.90 (s, 1H), 3.82 (s, 3H), 1.43 (s, 9H), 1.38 (s, 9H). The spectra data was consistent with the reported literature.

6.5 Adduct 1-1 catalyzed carboxylation of 3ac with CO_2

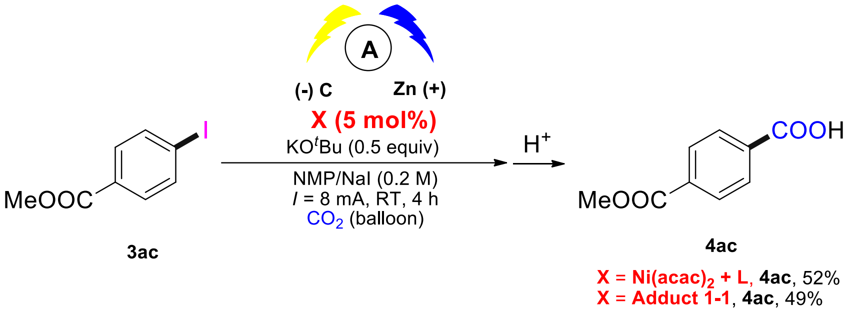

In a 50 mL three-neck flask equipped with a carbon felt cathode (1 cm × 1 cm × 2 cm) and a Zn plate (1 cm × 2 cm), aryl iodine **3ac** (0.3 mmol), adduct **1-1** (0.015 mmol), KO^{t}Bu (0.15 mmol), NaI (1 mmol) were loaded in the glove box. Then the mixture was taken out of the box, degassed under vacuum and back-filled with CO_2 gas for 5 times (each time lasted for 1 min). After that, 6 mL dry 1-methyl-2-pyrrolidinone (NMP) was injected into the flask via a syringe and dissolved the mixture under the strong stirring until the mixture becoming transparent. Then two electrodes were submerged into the solution (the effective surface of cathode: approximately 1 cm × 1 cm × 1 cm; anode: approximately 1 cm × 1 cm). Constant current (8 mA) was passed until the starting material was consumed for 4 hours at room temperature. After the electrolysis, the mixture was acidized by 2 N HCl solution (20 mL) and extracted by EtOAc for 4 times (30 mL × 4). The combined organic layers were washed with water (20 mL × 2) and brine (20 mL) and concentrated *in vacuo*. Then the residue was purified by silica gel column chromatography using petrolumn ether/EtOAc to give out the final product **4ac**.

6.6 Detection of adduct 1-2 from reaction mixture by HRMS

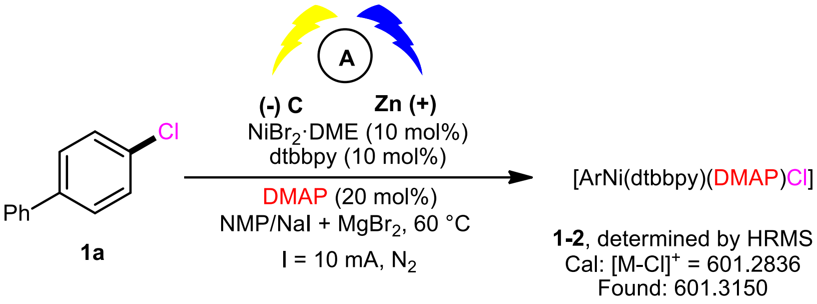

Procedure: In a 50 mL three-neck flask equipped with a carbon felt cathode (1 cm × 1 cm × 2 cm) and a Zn plate (1 cm × 2 cm), aryl chloride **1a** (0.3 mmol), $NiBr_2$•DME (0.015 mmol), dtbbpy (0.015 mmol), DMAP (0.06 mmol), $MgBr_2$ (0.45 mmol), NaI (1 mmol) were loaded in the glove box. Then the mixture was taken out of the box and 6 mL dry 1-methyl-2-pyrrolidinone (NMP) was injected into the flask via a syringe. Two electrodes were submerged into the solution (the effective surface of cathode: approximately 1 cm × 1 cm × 1 cm; anode: approximately 1 cm × 1 cm) and the flask was heated to 60 °C via oil bath. Constant current (10 mA) was passed and electrolyzed for 3 hours under N_2 atmosphere. Then reaction mixture was analyzed by HRMS.

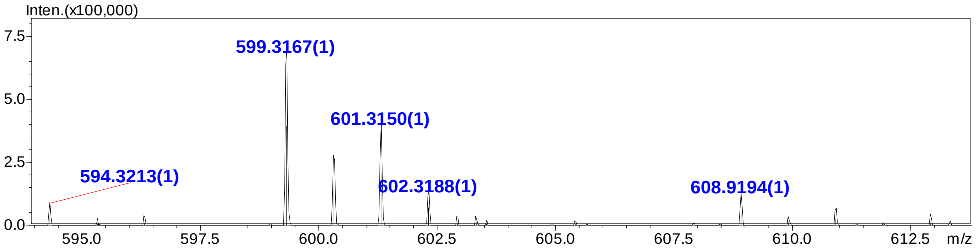

7. Consumed charge for substrates in the carboxylation

The consumed charge for each substrate is calculated by the following formula.

$$\text{F/mol} = \frac{I \times t}{F \times n}$$

I: current intensity, A; *t*: reaction time, s; *F*: faraday constant, ≈96500 C; *n*: mole of substrate, mol.

Supplementary Table 3. Scope of aryl chlorides, bromides, iodines and sulfonates and the corresponding consumed charge.

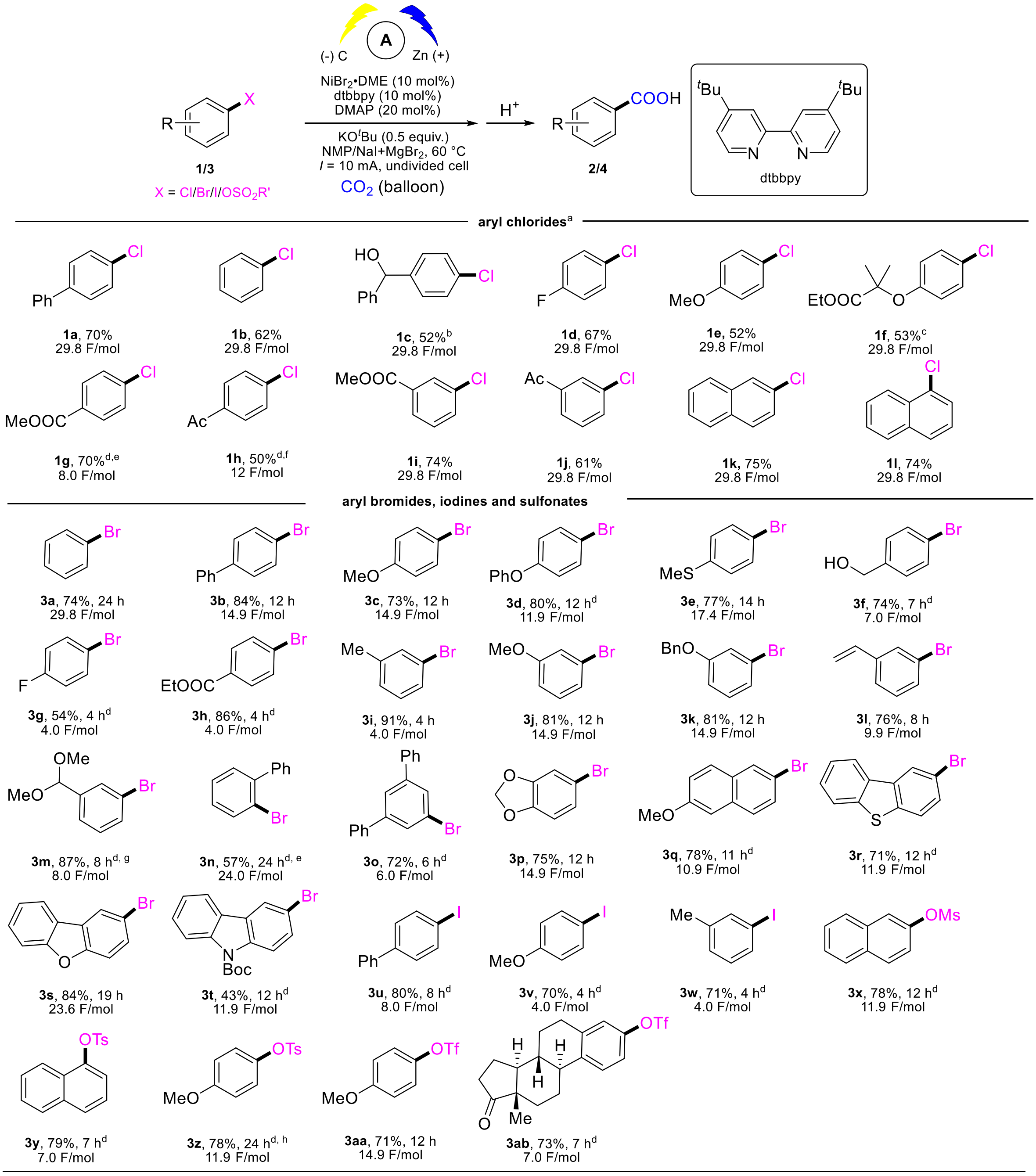

[a]0.3 mmol scale. [b]5 Å molecular sieve was added. [c]$NiBr_2$•DME (5 mol%), dtbbpy (5 mol%), DMAP (10 mol%). [d]$Ni(acac)_2$ (5 mol%), dtbbpy (5 mol%), KO^{t}Bu (0.5 equiv.), I = 8 mA. [e]50 °C, 8 h. [f]room temperature, 12 h. [g]3-formylbenzoic acid was obtained as product. [h]I = 4 mA.

Supplementary Table 4. Scope of alkyl bromides and the corresponding consumed charge.

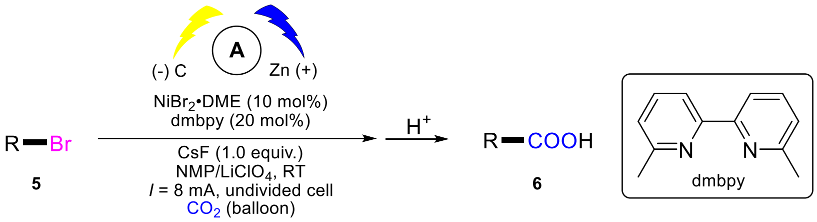

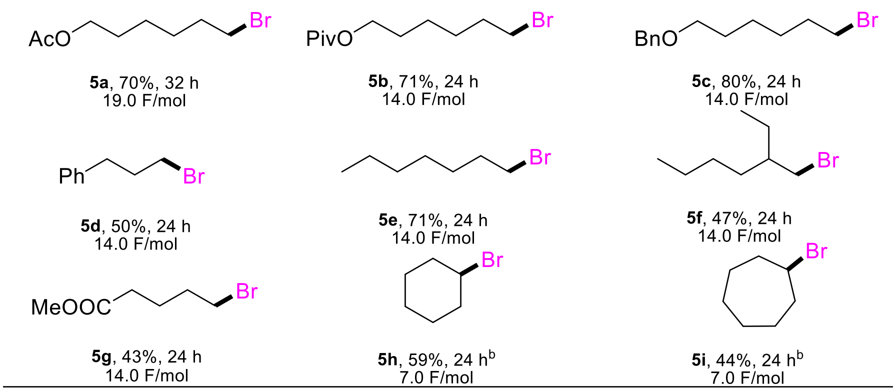

[a]0.5 mmol scale for the substrate. [b]I = 4 mA.

Supplementary Note: Most of inert aryl halides, especially the inert aryl chlorides, have consumed large amount of electricity (up to 29.8 F/mol). Even the non-activated alkyl bromides have also consumed 13 F/mol electricity in average. It is also noted that it takes longer time for the carboxylation with inert aryl halides and alkyl bromides. We suppose that in the cases of inert aryl chlorides, the rate of oxidative addition between inert C-Cl bond and nickel(0) center is slow and other undesirable electrochemical processes might exist such as Zn^{2+} precipitation (Zn^{2+} dissolved from zinc anode may compete to reduce on the cathode since its reduction potential is -0.76 V (vs SHE)) or hydrogen evolution reaction of residual water on the cathodic surface, which reduces the electrolytic efficiency and causes the extra charge consumed. Such non-productive processes consumed the charge and did not help production formation. Compared to the inert aryl chloride substrates, some other relatively active aryl halides such as **3h**, **3g**, **3i** (bromides), **3u**, **3v**, **3w** (iodides), **3y**, **3ab** (sulfonates) are in less consumed electricity (5 F/mol average, see in Table S3). We suppose that the rate of oxidative addition in these substrates with nickel catalyst is faster than the inert ones,

which reduces the electricity loss from other electrochemical processes.

8. Characterization data for carboxylic acids

[1,1'-biphenyl]-4-carboxylic acid (2a&4b&4u)[4]

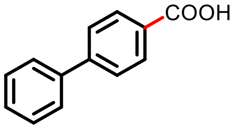

2a: 41.6 mg, 0.21 mmol, 70% with procedure 2.1;

4b: 49.9 mg, 0.252 mmol, 84% with procedure 2.1;

4u: 47.5 mg, 0.24 mmol, 80% with procedure 2.2;

4b: 41.6 mg, 0.21 mmol, 70% with procedure 2.4;

4b: 36.2 mg, 0.183 mmol, 61% with procedure 2.4.

White solid;

R$_f$ (PE/EA 1:1): 0.5;

^{1}H NMR (400 MHz, DMSO-*d*$_6$): δ = 12.96 (s, 1H), 8.03 (d, *J* = 8.4 Hz, 2H), 7.80 (d, *J* = 8.3 Hz, 2H), 7.74 (d, *J* = 7.6 Hz, 2H), 7.51 (t, *J* = 7.5 Hz, 2H), 7.43 (t, *J* = 7.3 Hz, 1H). **^{13}C NMR (101 MHz, DMSO-*d*$_6$):** δ = 167.6, 144.8, 139.5, 130.4, 130.1, 129.5, 128.7, 127.4, 127.3. **Exact Mass ESI-MS:** calculated m/z for [M-H$^+$]: 197.06, found: 196.94. The spectra data was consistent with the reported literature.

Benzoic acid (2b&4a)[4]

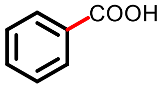

2b: 22.7 mg, 0.186 mmol, 62% with procedure 2.1;

4a: 27.1 mg, 0.222 mmol, 74% with procedure 2.1;

4a: 50.6 mg, 0.415 mmol, 83% with procedure 2.4 (0.5 mmol scale).

White solid;

R$_f$ (EA/PE 1:5): 0.5;

^{1}H NMR (400 MHz, $CDCl_3$): δ = 12.83 (s, 1H), 8.12 (dd, *J* = 8.2, 0.9 Hz, 2H), 7.65 – 7.49 (m, 1H), 7.50 – 7.42 (m, 2H). **^{13}C NMR (101 MHz, $CDCl_3$):** δ = 172.8, 133.9, 130.3, 129.4, 128.5. **Exact Mass ESI-MS:** calculated m/z for [M-H$^+$]: 121.03, found: 120.98. The spectra data was consistent with the reported literature.

4-(hydroxy(phenyl)methyl)benzoic acid (2c)[5]

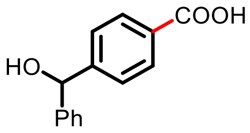

2c: 35.6 mg, 0.156 mmol, 52% with procedure 2.1;

2c: 38.3 mg, 0.168 mmol, 56% with procedure 2.4.

White solid;

R_f (PE/EA 1:2): 0.3;

^{1}H NMR (400 MHz, DMSO-*d_6*) δ = 12.80 (s, 1H), 7.85 (d, *J* = 8.3 Hz, 2H), 7.47 (d, *J* = 8.2 Hz, 2H), 7.37 – 7.32 (m, 2H), 7.30 – 7.23 (m, 2H), 7.20 – 7.14 (m, 1H), 6.02 (d, *J* = 3.8 Hz, 1H), 5.73 (d, *J* = 3.0 Hz, 1H). **^{13}C NMR (101 MHz, DMSO-*d_6*)** δ =δ = 167.6, 151.0, 145.5, 129.7, 129.6, 128.6, 127.4, 126.7, 126.7, 74.3. **Exact Mass ESI-MS:** calculated m/z for [M-H$^+$]: 227.07, found: 226.99.

4-fluorobenzoic acid (2d&4g)[4]

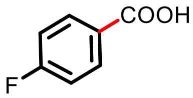

2d: 28.1 mg, 0.201 mmol, 67% with procedure 2.1;

4g: 22.7 mg, 0.162 mmol, 54% with procedure 2.2;

White solid;

R_f (PE/EA 1:1): 0.5

^{1}H NMR (400 MHz, $CDCl_3$): δ = 8.34 – 7.96 (m, 2H), 7.25 – 7.07 (m, 2H). **^{13}C NMR (101 MHz, $CDCl_3$):** δ = 171.0 (s), 166.4 (d, *J* = 255.3 Hz), 132.9 (d, *J* = 9.6 Hz), 125.5 (d, *J* = 2.9 Hz), 115.8 (d, *J* = 22.1 Hz). **^{19}F NMR (376 MHz, $CDCl_3$):** δ = -104.03. **Exact Mass ESI-MS:** calculated m/z for [M-H$^+$]: 139.02, found: 138.92. The spectra data was consistent with the reported literature.

4-methoxybenzoic acid (2e&4c&4v&4z&4aa)[4]

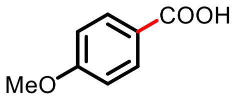

2e: 23.7 mg, 0.156 mmol, 52% with procedure 2.1;

4c: 33.3 mg, 0.219 mmol, 73% with procedure 2.1;

4v: 31.9 mg, 0.21 mmol, 70% with procedure 2.2;

4z: 35.6 mg, 0.234 mmol, 78% with procedure 2.2;

4aa: 32.4 mg, 0.213 mmol, 71% with procedure 2.1;

4aa: 35.1 mg, 0.231 mmol, 77% with procedure 2.4;

2e: 23.7 mg, 0.152 mmol, 52% with procedure 2.4.

White solid;

R_f (PE/EA 1:1): 0.33;

^{1}H NMR (400 MHz, $CDCl_3$): δ = 8.07 (d, 2H), 6.95 (d, 2H), 3.88 (s, 3H). **^{13}C NMR (101 MHz, $CDCl_3$):** δ = 171.5, 164.0, 132.4, 121.6, 113.8, 55.5. **Exact Mass ESI-MS:** calculated m/z for [M-H^+]: 151.04, found: 150.98. The spectra data was consistent with the reported literature.

4-((1-ethoxy-2-methyl-1-oxopropan-2-yl)oxy)benzoic acid (2f)[6]

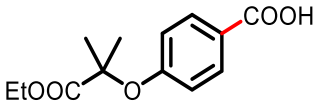

2f: 40.1 mg, 0.159 mmol, 53% with procedure 2.1;

2f: 37.8 mg, 0.150 mmol, 50% with procedure 2.4.

White solid;

R_f (PE/EA 1:2): 0.5;

^{1}H NMR (400 MHz, $CDCl_3$) δ = 7.98 (d, *J* = 8.8 Hz, 2H), 6.82 (d, *J* = 8.8 Hz, 2H), 4.21 (q, *J* = 7.1 Hz, 2H), 1.64 (s, 6H), 1.19 (t, *J* = 7.1 Hz, 3H). **^{13}C NMR (101 MHz, $CDCl_3$)** δ = 173.7, 171.7, 160.3, 131.9, 122.2, 117.2, 79.3, 61.7, 25.3, 14.0. **Exact Mass ESI-MS:** calculated m/z for [M-H^+]: 251.0925, found: 251.0929.

4-(methoxycarbonyl)benzoic acid (2g)[4]

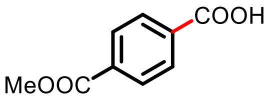

2g: 37.8 mg, 0.21 mmol, 70% with procedure 2.1;

2g: 37.8 mg, 0.21 mmol, 70% with procedure 2.4.

Yellow solid;

R_f (PE/EA 1:1): 0.2;

^{1}H NMR (400 MHz, DMSO-d_6): δ = 13.38 (s, 1H), 8.42 – 7.85 (m, 4H), 3.89 (s, 3H). **^{13}C NMR (101 MHz, DMSO-d_6):** δ = 169.1, 166.3, 139.0, 132.4, 130.0, 129.4, 52.7. **Exact Mass ESI-MS:** calculated m/z for [M-H$^+$]: 179.03, found: 178.96. The spectra data was consistent with the reported literature.

4-acetylbenzoic acid (2h)[4]

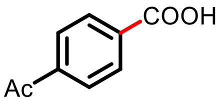

24.6 mg, 0.150 mmol, 50% with procedure 2.1;

Yellow solid;

R_f (PE/EA 1:1): 0.2;

^{1}H NMR (400 MHz, DMSO-d_6): δ = 13.28 (s, 1H), 8.06 (m, 4H), 2.64 (s, 3H). **^{13}C NMR (101 MHz, DMSO-d_6):** δ = 198.2, 167.1, 140.3, 135.0, 130.0, 128.8, 27.5. **Exact Mass ESI-MS:** calculated m/z for [M-H$^+$]: 163.04, found: 162.97. The spectra data was consistent with the reported literature.

3-(methoxycarbonyl) benzoic acid (2i)[2]

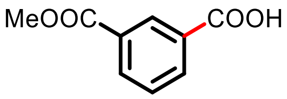

2i: 40.0 mg, 0.222 mmol, 74% with procedure 2.1;

2i: 36.2 mg, 0.201 mmol, 67% with procedure 2.4.

White solid;

R_f (PE/EA 1:1): 0.45;

^{1}H NMR (400 MHz, DMSO-d_6): δ = 13.34 (s, 1H), 8.49 (d, J = 1.7 Hz, 1H), 8.24 – 8.16 (m, 2H), 7.68 (t, J = 7.8 Hz, 1H), 3.90 (s, 3H). **^{13}C NMR (101 MHz, DMSO-d_6):** δ = 166.9, 166.0, 134.2, 133.7, 131.8, 130.5, 130.2, 129.8, 52.9. **Exact Mass ESI-MS:** calculated m/z for [M-H$^+$]: 179.03, found: 178.95. The spectra data was consistent with the reported literature.

3-acetylbenzoic acid (2j) [2]

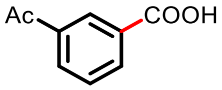

30.0 mg, 0.183 mmol, 61% with procedure 2.1;

White solid;

R$_f$ (PE/EA 1:2): 0.5;

^{1}H NMR (400 MHz, DMSO-d_6) δ = 13.22 (s, 1H), 8.46 (s, 1H), 8.28 – 8.14 (m, 2H), 7.67 (t, J = 7.7 Hz, 1H), 2.64 (s, 3H). **^{13}C NMR (101 MHz, DMSO-d_6)** δ = 197.8, 167.1, 137.4, 134.0, 132.8, 131.7, 129.7, 129.1, 27.3. **Exact Mass ESI-MS:** calculated m/z for [M-H$^+$]: 163.04, found: 162.98.

2-naphthoic acid (2k&4x)[4]

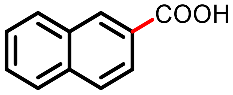

2k:38.7 mg, 0.225 mmol, 75% with procedure 2.1;

4x: 40.2 mg, 0.234 mmol, 78% with procedure 2.2;

4x: 38.2 mg, 0.222 mmol, 74% with procedure 2.4.

White solid;

R$_f$ (PE/EA 1:1): 0.5;

^{1}H NMR (400 MHz, DMSO-d_6): δ = 13.06 (s, 1H), 8.64 (s, 1H), 8.13 (d, J = 7.7 Hz, 1H), 8.07 – 7.95 (m, 3H), 7.72 – 7.56 (m, 2H). **^{13}C NMR (101 MHz, DMSO-d_6):** δ = 167.9, 135.4, 132.6, 131.0, 129.7, 128.8, 128.6, 128.5, 128.1, 127.3, 125.6. **Exact Mass ESI-MS:** calculated m/z for [M-H$^+$]: 171.05, found: 170.98.

1-naphthoic acid (2l&4y)[4]

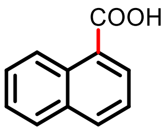

2l: 38.2 mg, 0.222 mmol, 74% with procedure 2.1;

4y: 40.8 mg, 0.237 mmol, 79% with procedure 2.2;

4y: 46.4 mg, 0.270 mmol, 90% with procedure 2.4.

2l: 33.0 mg, 0.192 mmol, 64% with procedure 2.4.

White solid;

R_f (PE/EA 1:1): 0.4;

^{1}H NMR (400 MHz, DMSO-*d*$_6$): δ = 13.15 (s, 1H), 8.87 (d, *J* = 8.5 Hz, 1H), 8.16 (d, *J* = 7.3 Hz, 2H), 8.03 (d, *J* = 7.7 Hz, 1H), 7.71 – 7.53 (m, 3H). **^{13}C NMR (101 MHz, DMSO-*d*$_6$):** δ = 169.1, 139.9, 133.4, 131.2, 130.3, 129.1, 128.2, 128.0, 126.7, 126.0, 125.4. **Exact Mass ESI-MS:** calculated m/z for [M-H$^+$]: 171.05, found: 170.93. The spectra data was consistent with the reported literature.

4-phenoxybenzoic acid (4d)[2]

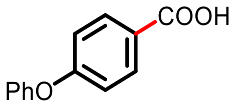

4d: 51.4 mg, 0.24 mmol, 80% with procedure 2.2;

4d: 53.9 mg, 0.252 mmol, 84% with procedure 2.4.

White solid;

R_f (PE/EA 1:1): 0.3;

^{1}H NMR (400 MHz, $CDCl_3$) δ = 8.08 (d, *J* = 8.8 Hz, 2H), 7.41 (t, *J* = 7.9 Hz, 2H), 7.21 (t, *J* = 7.4 Hz, 1H), 7.09 (d, *J* = 7.8 Hz, 2H), 7.01 (d, *J* = 8.8 Hz, 2H). **^{13}C NMR (101 MHz, $CDCl_3$)** δ = 171.6, 162.7, 155.4, 132.5, 130.1, 124.7, 123.4, 120.3, 117.2. **Exact Mass ESI-MS:** calculated m/z for [M-H$^+$]: 213.06, found: 212.97. The spectra data was consistent with the reported literature.

4-(methylthio)benzoic acid (4e)[4]

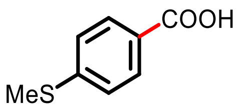

4e: 38.8 mg, 0.231 mmol, 77% with procedure 2.1;

4e: 33.3 mg, 0.198 mmol, 66% with procedure 2.4.

White solid;

R_f (PE/EA 1:1): 0.35;

^{1}H NMR (400 MHz, $CDCl_3$): δ = 8.00 (d, *J* = 8.6 Hz, 2H), 7.28 (d, *J* = 8.6 Hz, 2H), 2.53 (s, 3H). **^{13}C NMR (101 MHz, $CDCl_3$)**: δ = 171.3, 146.7, 130.5, 125.2, 124.9, 14.8. **Exact Mass ESI-MS:** calculated m/z for [M-H^+]: 167.02, found: 166.97. The spectra data was consistent with the reported literature.

4-(hydroxymethyl) benzoic acid (4f)[7]

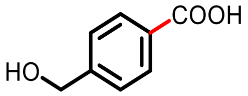

4f: 33.7 mg, 0.222 mmol, 74% with procedure 2.2;

4f: 34.2 mg, 0.225 mmol, 75% with procedure 2.4.

White solid;

R_f (PE/EA 1:1): 0.24;

^{1}H NMR (400 MHz, DMSO-*d*$_6$) δ = 12.81 (s, 1H), 7.91 (d, *J* = 8.2 Hz, 2H), 7.44 (d, *J* = 8.1 Hz, 2H), 4.58 (s, 2H). **^{13}C NMR (101 MHz, DMSO-*d*$_6$)**: δ = 167.8, 148.3, 129.7, 129.6, 126.6, 62.9. **Exact Mass ESI-MS:** calculated m/z for [M-H^+]: 151.04, found: 150.95. The spectra data was consistent with the reported literature.

4-(ethoxycarbonyl) benzoic acid (4h)[2]

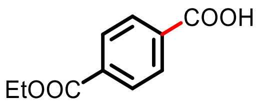

50.6 mg, 0.261 mmol, 86% with procedure 2.2;

White solid;

R_f (PE/EA 1:1): 0.3;

^{1}H NMR (400 MHz, CDCl$_3$): δ = 8.17 (m, 4H), 4.43 (q, J = 7.1 Hz, 2H), 1.43 (t, J = 7.1 Hz, 3H); **^{13}C NMR (101 MHz, CDCl$_3$):** δ = 171.1, 165.7, 135.1, 132.9, 130.2, 129.6, 61.6, 14.3. **Exact Mass ESI-MS:** calculated m/z for [M-H$^+$]: 193.05, found: 192.92. The spectra data was consistent with the reported literature.

3-methylbenzoic acid (4i&4w)[4]

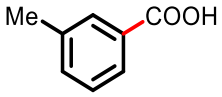

4i: 37.2 mg, 0.273 mmol, 91% with procedure 2.1;

4w: 29.0 mg, 0.213 mmol, 71% with procedure 2.2;

White solid;

R$_f$ (PE/EA 1:1): 0.5;

^{1}H NMR (400 MHz, CDCl$_3$): δ = 11.23 (s, 1H), 7.93 (d, J = 7.9 Hz, 2H), 7.42 (d, J = 7.5 Hz, 1H), 7.36 (t, J = 7.6 Hz, 1H), 2.42 (s, 3H). **^{13}C NMR (101 MHz, CDCl$_3$):** δ = 172.6, 138.3, 134.6, 130.7, 129.3, 128.4, 127.4, 21.3. **Exact Mass ESI-MS:** calculated m/z for [M-H$^+$]: 135.05, found: 135.01. The spectra data was consistent with the reported literature.

3-methoxybenzoic acid (4j)[2]

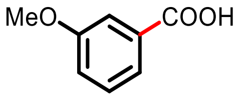

4j: 36.9 mg, 0.243 mmol, 81% with procedure 2.1;

4j: 35.1 mg, 0.231 mmol, 77% with procedure 2.4.

White solid;

R$_f$ (PE/EA 1:1): 0.4;

^{1}H NMR (400 MHz, CDCl$_3$): δ = 7.73 (d, J = 7.7 Hz, 1H), 7.63 (dd, J = 2.4, 1.5 Hz, 1H), 7.39 (t, J = 7.9 Hz, 1H), 7.16 (ddd, J = 13.2, 7.0, 1.1 Hz, 1H), 3.87 (s, 3H). **^{13}C NMR (101 MHz, CDCl$_3$):** δ = 172.3, 159.6, 130.6, 129.6, 122.7, 120.5, 114.4, 55.5. **Exact Mass ESI-MS:** calculated m/z for [M-H$^+$]: 151.04, found: 150.98. The spectra data was consistent with the reported literature.

3-(benzyloxy)benzoic acid (4k)[8]

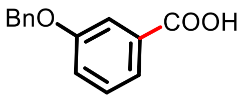

4k: 55.4 mg, 0.243 mmol, 81% with procedure 2.1;

4k: 47.9 mg, 0.210 mmol, 70% with procedure 2.4.

White solid;

R_f (PE/EA 1:1): 0.3;

^{1}H NMR (400 MHz, $CDCl_3$): δ = 7.73 (t, *J* = 5.2 Hz, 2H), 7.49 – 7.44 (m, 2H), 7.44 – 7.38 (m, 3H), 7.36 (dd, *J* = 5.9, 4.3 Hz, 1H), 7.23 (ddd, *J* = 8.3, 2.5, 0.9 Hz, 1H), 5.13 (s, 2H). **^{13}C NMR (101 MHz, $CDCl_3$):** δ = 158.8, 136.5, 130.6, 129.6, 128.7, 128.2, 127.6, 123.0, 121.2, 115.5, 100.0, 70.2. **Exact Mass ESI-MS:** calculated m/z for [M-H$^+$]: 227.07, found: 226.98. The spectra data was consistent with the reported literature.

3-vinylbenzoic acid (4l)

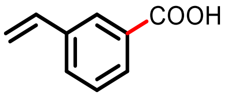

33.7 mg, 0.228 mmol, 76% with procedure 2.1;

White solid;

R_f (PE/EA 1:1): 0.6;

^{1}H NMR (400 MHz, DMSO-*d*$_6$) δ = 12.99 (s, 1H), 8.00 (t, *J* = 1.8 Hz, 1H), 7.85 (dt, *J* = 7.7, 1.5 Hz, 1H), 7.74 (dd, *J* = 7.7, 1.8 Hz, 1H), 7.48 (t, *J* = 7.7 Hz, 1H), 6.82 (dd, *J* = 17.7, 10.9 Hz, 1H), 5.91 (dd, *J* = 17.8, 1.1 Hz, 1H), 5.34 (d, *J* = 10.9 Hz, 1H).

^{13}C NMR (101 MHz, DMSO-*d*$_6$): δ = 167.7, 137.9, 136.4, 131.7, 130.6, 129.1, 129.2, 127.5, 116.0. **Exact Mass ESI-MS:** calculated m/z for [M-H$^+$]: 147.05, found: 146.97.

3-formylbenzoic acid (4m)[9]

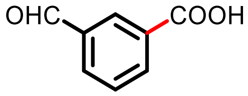

39.2 mg, 0.261 mmol, 87% with procedure 2.2;

White solid;

R_f (PE/EA 1:1): 0.4;

^{1}**H NMR (400 MHz, DMSO-*d*$_6$)** δ = 13.33 (s, 1H), 10.10 (s, 1H), 8.45 (s, 1H), 8.19 (d, *J* = 39.5 Hz, 2H), 7.75 (s, 1H). ^{13}**C NMR (101 MHz, DMSO-*d*$_6$)** δ = 193.3, 166.9, 136.9, 135.3, 133.5, 132.2, 130.8, 130.1. **Exact Mass ESI-MS:** calculated m/z for [M-H$^+$]: 149.02, found: 148.93. The spectra data was consistent with the reported literature.

[1,1'-biphenyl]-2-carboxylic acid (4n)[10]

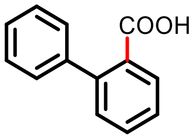

4n: 33.9 mg, 0.171 mmol, 57% with procedure 2.2;

4n: 30.3 mg, 0.153 mmol, 51% with procedure 2.4.

White solid;

R$_f$ (PE/EA 1:1): 0.3;

^{1}**H NMR (400 MHz, DMSO-*d*$_6$):** δ = 12.77 (s, 1H), 7.73 – 7.65 (m, 1H), 7.58 – 7.51 (m, 1H), 7.47 – 7.26 (m, 7H). ^{13}**C NMR (101 MHz, DMSO-*d*$_6$):** δ = 170.2, 141.4, 141.3, 132.8, 131.3, 130.9, 129.5, 128.8, 128.6, 127.7, 127.6. **Exact Mass ESI-MS:** calculated m/z for [M-H$^+$]: 197.06, found: 196.96. The spectra data was consistent with the reported literature.

[1,1':3',1''-terphenyl]-5'-carboxylic acid (4o)[11]

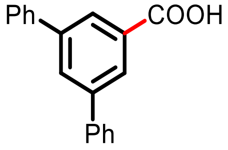

4o: 59.2 mg, 0.216 mmol, 72% with procedure 2.2;

4o: 68.2 mg, 0.249 mmol, 83% with procedure 2.4.

Yellow solid;

R$_f$ (PE/EA 1:2): 0.2;

^{1}**H NMR (400 MHz, DMSO-*d*$_6$)**: δ = 13.21 (s, 1H), 8.16 (d, *J* = 10.2 Hz, 3H), 7.82 (d, *J* = 7.4 Hz, 4H), 7.53 (t, *J* = 7.5 Hz, 4H), 7.44 (t, *J* = 7.3 Hz, 2H). ^{13}**C NMR (101 MHz, DMSO-*d*$_6$)**: δ = 167.6, 141.9, 139.7, 132.7, 129.9, 129.6, 128.5, 127.6, 126.9. **Exact Mass ESI-MS:** calculated m/z for [M-H$^+$]: 273.09, found: 272.98. The spectra data was consistent with the reported literature.

Benzo[*d*][1,3]dioxole-5-carboxylic acid (4p)[4]

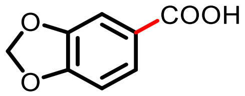

4p: 37.4 mg, 0.225 mmol, 75% with procedure 2.1;

4p: 41.3 mg, 0.249 mmol, 83% with procedure 2.4.

White solid;

R_f (PE/EA 1:1): 0.45;

^{1}H NMR (400 MHz, DMSO-d$_6$): δ = 12.74 (s, 1H), 7.55 (dd, *J* = 8.1, 1.7 Hz, 1H), 7.36 (d, *J* = 1.6 Hz, 1H), 7.00 (d, *J* = 8.1 Hz, 1H), 6.12 (s, 2H). **^{13}C NMR (101 MHz, DMSO-*d*$_6$):** δ = 166.6, 151.1, 147.4, 124.9, 124.6, 108.7, 108.0, 101.9. **Exact Mass ESI-MS:** calculated m/z for [M-H$^+$]: 165.02, found: 164.99. The spectra data was consistent with the reported literature.

6-methoxy-2-naphthoic acid (4q)[12]

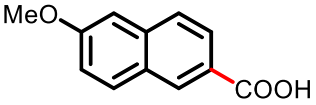

4q: 47.3 mg, 0.234 mmol, 78% with procedure 2.2;

4q: 30.3 mg, 0.150 mmol, 50% with procedure 2.4.

White solid;

R_f (PE/EA 1:1): 0.5;

^{1}H NMR (400 MHz, DMSO-*d*$_6$): δ = 12.92 (s, 1H), 8.54 (s, 1H), 8.02 (d, *J* = 9.0 Hz, 1H), 7.95 (d, *J* = 8.4 Hz, 1H), 7.88 (d, *J* = 8.4 Hz, 1H), 7.39 (d, *J* = 1.6 Hz, 1H), 7.24 (dd, *J* = 8.9, 2.2 Hz, 1H), 3.90 (s, 3H). **^{13}C NMR (101 MHz, DMSO-*d*$_6$):** δ = 159.6, 137.2, 131.4, 130.9, 128.0, 127.4, 126.3, 119.9, 106.4, 55.8. **Exact Mass ESI-MS:** calculated m/z for [M-H$^+$]: 201.06, found: 201.03. The spectra data was consistent with the reported literature.

Dibenzo[*b,d*]thiophene-2-carboxylic acid (4r)[13]

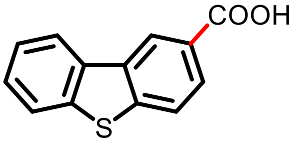

4r: 48.6 mg, 0.213 mmol, 71% with procedure 2.2;

4r: 52.7 mg, 0.231 mmol, 77% with procedure 2.4.

White solid;

m.p. : 250-252 °C;

R_f (PE/EA 1:1): 0.3;

^{1}H NMR (400 MHz, DMSO-*d₆*): δ = 13.11 (s, 1H), 8.91 (d, *J* = 1.2 Hz, 1H), 8.56 – 8.47 (m, 1H), 8.16 (d, *J* = 8.4 Hz, 1H), 8.08 (ddd, *J* = 8.5, 5.4, 1.8 Hz, 2H), 7.64 – 7.52 (m, 2H). **^{13}C NMR (101 MHz, DMSO-d_6):** δ = 167.8, 143.8, 139.4, 135.5, 135.1, 128.1, 128.0, 127.8, 125.7, 123.7, 123.6, 123.5, 122.9. **Exact Mass ESI-MS:** calculated m/z for [M-H$^+$]: 227.0172, found: 227.0167.

Dibenzo[*b,d*]furan-2-carboxylic acid (4s)[14]

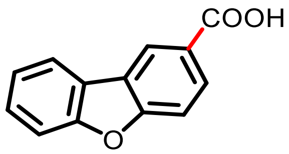

4s: 53.4 mg, 0.252 mmol, 84% with procedure 2.1;

4s: 56.6 mg, 0.267 mmol, 89% with procedure 2.4.

White solid;

R_f (PE/EA 1:1): 0.7;

^{1}H NMR (400 MHz, DMSO-d_6): δ = 13.03 (s, 1H), 8.80 (d, *J* = 1.6 Hz, 1H), 8.31 (d, *J* = 7.4 Hz, 1H), 8.14 (dd, *J* = 8.6, 1.8 Hz, 1H), 7.78 (dd, *J* = 17.1, 8.4 Hz, 2H), 7.66 – 7.56 (m, 1H), 7.46 (t, *J* = 7.3 Hz, 1H). **^{13}C NMR (101 MHz, DMSO-d_6):** δ = 167.6, 158.4, 156.5, 129.5, 128.7, 126.4, 124.3, 124.1, 123.5, 123.5, 122.2, 112.3, 112.1. **Exact Mass ESI-MS:** calculated m/z for [M-H$^+$]: 211.04, found: 211.01. The spectra data was consistent with the reported literature.

9-(*tert*-butoxycarbonyl)-9H-carbazole-3-carboxylic acid (4t)

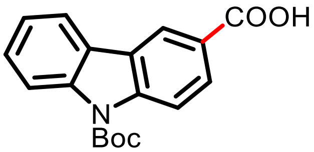

40.1 mg, 0.129 mmol, 43% with procedure 2.2;

White solid;

R$_f$ (PE/EA 1:1): 0.33;

m.p.: 180-182 °C;

^{1}H NMR (400 MHz, DMSO-*d$_6$*): δ = 8.74 (s, 1H), 8.35 – 8.21 (m, 3H), 8.13 (dd, *J* = 8.7, 1.8 Hz, 1H), 7.61 – 7.54 (m, 1H), 7.49 – 7.41 (m, 1H), 1.75 (s, 9H). **^{13}C NMR (101 MHz, DMSO-*d$_6$*):** δ = 167.8, 150.5, 140.8, 138.6, 128.9, 128.3, 126.1, 125.5, 125.0, 124.0, 122.0, 120.9, 116.3, 116.0, 85.2, 28.2. **Exact Mass ESI-MS:** calculated m/z for [M-H$^+$]: 310.1085, found: 310.1086.

(8*R*,9*S*,13*S*,14*S*)-13-methyl-17-oxo-7,8,9,11,12,13,14,15,16,17-decahydro-6*H* cyclopenta[*a*]phenanthrene-3-carboxylic acid (4ab)[15]

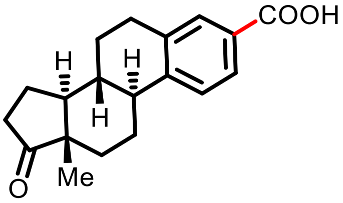

4ab: 65.3 mg, 0.219 mmol, 73% with procedure 2.2;

4ab: 73.3 mg, 0.246 mmol, 82% with procedure 2.4.

White solid;

R$_f$ (PE/EA 1:3): 0.25;

^{1}H NMR (400 MHz, DMSO-*d$_6$*): δ = 12.80 (s, 1H), 7.71 (dd, *J* = 12.2, 5.0 Hz, 2H), 7.43 (d, *J* = 8.1 Hz, 1H), 2.92 (dd, *J* = 7.2, 4.6 Hz, 2H), 2.55 (t, *J* = 1.8 Hz, 1H), 2.48 – 2.42 (m, 1H), 2.35 – 2.26 (m, 1H), 2.10 (d, *J* = 9.7 Hz, 1H), 2.04 – 1.95 (m, 2H), 1.81 (dd, *J* = 7.0, 4.7 Hz, 1H), 1.67 – 1.51 (m, 3H), 1.52 – 1.39 (m, 3H), 0.86 (s, 3H). **^{13}C NMR (101 MHz, DMSO-*d$_6$*):** δ = 219.9, 167.8, 145.3, 137.0, 130.2, 128.5, 127.0, 126.0, 50.1, 47.7, 44.5, 37.7, 35.8, 31.7, 29.2, 26.2, 25.6, 21.6, 13.9. **Exact Mass ESI-MS:** calculated m/z for [M-H$^+$]: 297.15, found: 297.06. The spectra data was consistent with the reported literature.

4-(*tert*-butyl) benzoic acid (4ac)[23]

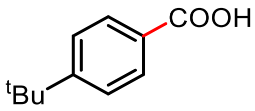

37.4 mg, 0.21 mmol, 70%

White solid

R$_f$ (PE/EA 1:1): 0.26

^{1}H NMR (400 MHz, CDCl$_3$): δ = 8.05 (d, *J* = 8.4 Hz, 2H), 7.49 (d, 2H), 1.35 (s, 9H). **^{13}C NMR (101 MHz, CDCl$_3$)** δ = 171.9, 157.6, 130.1, 126.5, 125.5, 35.2, 31.1. **Exact Mass ESI-MS:** calculated m/z for [M-H$^+$]: 177.09, found: 177.03.

4-(trifluoromethoxy)benzoic acid (4ad)[20]

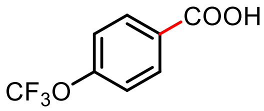

45.1 mg, 0.219 mmol, 73% with procedure 2.4.

White solid

R$_f$ (PE/EA 1:1): 0.3

^{1}H NMR (400 MHz, CDCl$_3$) δ = 8.23 – 8.08 (m, 2H), 7.32 – 7.26 (m, 2H). **^{13}C NMR (101 MHz, CDCl$_3$)** δ = 171.0, 153.4, 132.3, 127.6, 120.3 (d, *J* = 259.0 Hz), 120.3. **^{19}F NMR (376 MHz, CDCl$_3$)** δ = -57.64. **Exact Mass ESI-MS:** calculated m/z for [M-H$^+$]: 205.01, found: 204.98. The spectra data was consistent with the reported literature.

3,5-di-*tert*-butylbenzoic acid (4ae)[21]

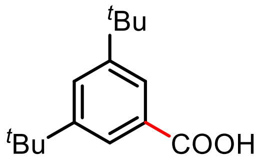

59 mg, 0.252 mmol, 84% with procedure 2.4.

White solid

R$_f$ (PE/EA 1:1): 0.7

^{1}H NMR (400 MHz, CDCl$_3$) δ = 7.98 (d, *J* = 1.8 Hz, 2H), 7.69 (t, *J* = 1.9 Hz, 1H), 1.37 (s, 18H). **^{13}C NMR (101 MHz, CDCl$_3$):** δ = 173.3, 151.2, 128.7, 128.1, 124.5, 35.0, 31.4. **Exact Mass ESI-MS:**

calculated m/z for [M-H$^+$]: 233.15, found: 233.05. The spectra data was consistent with the reported literature.

4-(trifluoromethyl)benzoic acid (2m)[22]

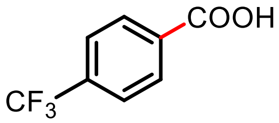

41.6 mg, 0.219 mmol, 73% with procedure 2.4.

White solid

R$_f$ (PE/EA 1:1): 0.3;

^{1}H NMR (400 MHz, DMSO-*d*$_6$) δ = 13.46 (s, 1H), 8.10 (d, *J* = 8.1 Hz, 2H), 7.85 (d, *J* = 8.2 Hz, 2H). **^{13}C NMR (101 MHz, DMSO-*d*$_6$)** δ = 166.7, 135.1, 133.1, 132.8, 130.6, 126.1 (d, *J* = 3.6 Hz). **^{19}F NMR (376 MHz, DMSO)** δ = -61.56. **Exact Mass ESI-MS:** calculated m/z for [M-H$^+$]: 189.02, found: 188.92. The spectra data was consistent with the reported literature.

7-acetoxyheptanoic acid (6a)

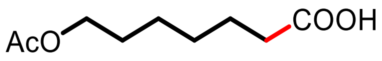

6a: 65.8mg, 0.35 mmol, 70% with procedure 2.3;

Yellow oil;

R$_f$ (PE/EA 1:1): 0.4;

^{1}H NMR (400 MHz, CDCl$_3$) δ = 4.06 (t, *J* = 6.7 Hz, 2H), 2.36 (t, *J* = 7.4 Hz, 2H), 2.05 (s, 3H), 1.75 – 1.56 (m, 4H), 1.54 – 1.24 (m, 4H). **^{13}C NMR (101 MHz, CDCl$_3$)** δ = 179.7, 171.4, 64.5, 33.9, 28.6, 28.4, 25.6, 24.5, 21.0. **Exact Mass ESI-MS:** calculated m/z for [M-H$^+$]: 187.10, found: 186.90.

7-(pivaloyloxy)heptanoic acid (6b)[16]

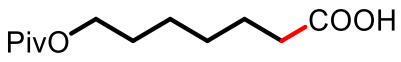

6b: 81.7 mg, 0.355 mmol, 71% with procedure 2.3;

6b: 31.1 mg, 0.135 mmol, 27% with procedure 2.4.

Colorless oil;

Rf (PE/EA 5:1): 0.5;

^{1}H NMR (400 MHz, CDCl$_3$) δ = 4.05 (t, *J* = 6.6 Hz, 2H), 2.36 (t, *J* = 7.5 Hz, 2H), 1.70 – 1.60 (m, 4H), 1.42 – 1.36 (m, 4H), 1.19 (s, 9H). **^{13}C NMR (101 MHz, CDCl$_3$)** δ = 180.0, 178.7, 64.3, 38.7, 33.9, 28.6, 28.3, 27.1, 25.6, 24.5. **Exact Mass ESI-MS:** calculated m/z for [M-H$^+$]: 229.14, found: 228.89. The spectra data was consistent with the reported literature.

7-(benzyloxy)heptanoic acid (6c)[16]

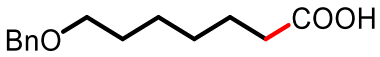

6c: 94.4 mg, 0.4 mmol, 80% with procedure 2.3;

6c: 18.9 mg, 0.08 mmol, 16% with procedure 2.4.

Yellow oil;

Rf (PE/EA 3:1): 0.3;

^{1}H NMR (400 MHz, CDCl$_3$) δ = 7.37 – 7.31 (m, 4H), 7.30 – 7.25 (m, 1H), 4.49 (s, 2H), 3.45 (t, *J* = 6.6 Hz, 2H), 2.33 (t, *J* = 7.5 Hz, 2H), 1.70 – 1.56 (m, 4H), 1.46 – 1.29 (m, 4H). **^{13}C NMR (101 MHz, CDCl$_3$)** δ = 180.1, 138.5, 128.3, 127.6, 127.5, 72.8, 70.2, 34.0, 29.5, 28.9, 25.8, 24.6. **Exact Mass ESI-MS:** calculated m/z for [M-H$^+$]: 235.13, found: 234.96. The spectra data was consistent with the reported literature.

4-phenylbutanoic acid (6d)[17]

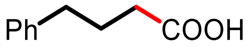

6d: 41 mg, 0.25 mmol, 50% with procedure 2.3;

6d: 36.9 mg, 0.225 mmol, 45% with procedure 2.4.

White solid;

Rf (PE/EA 5:1): 0.5;

^{1}H NMR (400 MHz, CDCl$_3$) δ = 7.24 – 7.17 (m, 2H), 7.16 – 7.07 (m, 3H), 2.59 (t, 2H), 2.29 (t, *J* = 7.4 Hz, 2H), 1.95 – 1.82 (m, 2H). **^{13}C NMR (101 MHz, CDCl$_3$)** δ = 180.2, 141.2, 128.5, 128.4, 126.0, 35.0, 33.4, 26.2. **Exact Mass ESI-MS:** calculated m/z for [M-H$^+$]: 163.08, found: 162.92. The spectra data was consistent with the reported literature.

Octanoic acid (6e)

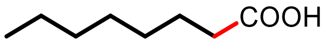

51.1 mg, 0.355 mmol, 71% with procedure 2.3;

Yellow oil;

R_f (PE/EA 3:1): 0.3;

^{1}H NMR (400 MHz, $CDCl_3$) δ = 2.35 (t, *J* = 7.5 Hz, 2H), 1.70 – 1.56 (m, 2H), 1.39 – 1.20 (m, 8H), 0.88 (t, *J* = 6.9 Hz, 3H). **^{13}C NMR (101 MHz, $CDCl_3$)** δ = 180.5, 34.1, 31.6, 29.0, 28.9, 24.6, 22.6, 14.0. **Exact Mass ESI-MS:** calculated m/z for [M-H$^+$]: 143.11, found: 142.93.

3-ethylheptanoic acid (6f)[2]

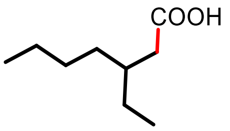

37.1 mg, 0.235 mmol, 47% with procedure 2.3;

Colorless oil;

R_f (PE/EA 5:1): 0.5;

^{1}H NMR (400 MHz, $CDCl_3$) δ 2.28 (d, *J* = 6.8 Hz, 2H), 1.89 – 1.75 (m, 1H), 1.39 – 1.24 (m, 8H), 0.94 – 0.84 (m, 6H). **^{13}C NMR (101 MHz, $CDCl_3$)** δ = 180.1, 38.6, 36.2, 32.9, 28.7, 26.2, 22.9, 14.1, 10.7. **Exact Mass ESI-MS:** calculated m/z for [M-H$^+$]: 157.12, found: 156.88. The spectra data was consistent with the reported literature.

6-methoxy-6-oxohexanoic acid (6g)[18]

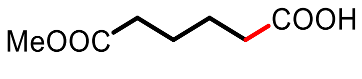

34.4 mg, 0.215 mmol, 43% with procedure 2.3;

Colorless oil;

R_f (PE/EA 1:1): 0.2;

^{1}H NMR (400 MHz, $CDCl_3$) δ = 3.67 (s, 3H), 2.36 (dt, *J* = 14.0, 7.0 Hz, 4H), 1.85 – 1.53 (m, 4H). **^{13}C NMR (101 MHz, $CDCl_3$)** δ = 179.2, 173.8, 51.6, 33.6, 33.6, 24.2, 24.0. **Exact Mass ESI-MS:**

calculated m/z for [M-H+]: 159.07, found: 158.95. The spectra data was consistent with the reported literature.

Cyclohexanecarboxylic acid (6h)[2]

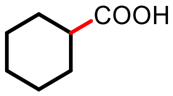

37.8 mg, 0.295 mmol, 59% with procedure 2.3;

Colorless oil;

R_f (PE/EA 5:1): 0.4;

^{1}H NMR (400 MHz, $CDCl_3$) δ = 2.26 (tt, *J* = 11.2, 3.6 Hz, 1H), 1.92 – 1.80 (m, 2H), 1.78 – 1.64 (m, 2H), 1.58 (dd, *J* = 10.1, 4.2 Hz, 1H), 1.45 – 1.33 (m, 2H), 1.28 – 1.12 (m, 3H). **^{13}C NMR (101 MHz, $CDCl_3$)** δ = 182.5, 42.9, 28.7, 25.6, 25.3. **Exact Mass ESI-MS:** calculated m/z for [M-H+]: 127.08, found: 126.98. The spectra data was consistent with the reported literature.

Cycloheptanecarboxylic acid (6i)[19]

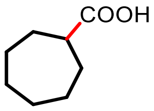

31.2 mg, 0.22 mmol, 44% with procedure 2.3;

Colorless oil;

R_f (PE/EA 5:1): 0.5;

^{1}H NMR (400 MHz, $CDCl_3$) δ = 2.51 (tt, *J* = 9.0, 4.4 Hz, 1H), 2.02 – 1.91 (m, 2H), 1.78 – 1.64 (m, 4H), 1.63 – 1.40 (m, 6H). **^{13}C NMR (101 MHz, $CDCl_3$)** δ = 183.4, 44.7, 30.6, 28.3, 26.2. **Exact Mass ESI-MS:** calculated m/z for [M-H+]: 141.09, found: 140.96. The spectra data was consistent with the reported literature.

9. The spectrums of ^{1}H NMR, ^{19}F NMR and ^{13}C NMR

[1,1'-biphenyl]-4-carboxylic acid (2a&4b&4u, *DMSO-d$_6$* as solvent)

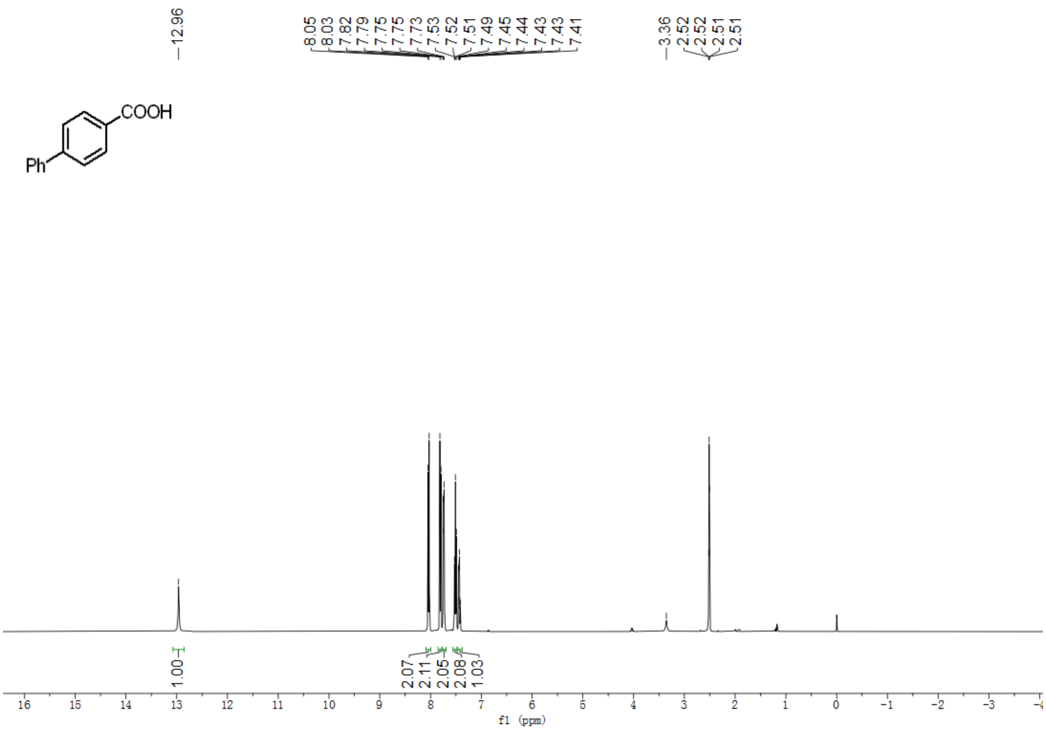

Supplementary Figure 4. ^{1}H NMR spectra of compound **2a&4b&4u**

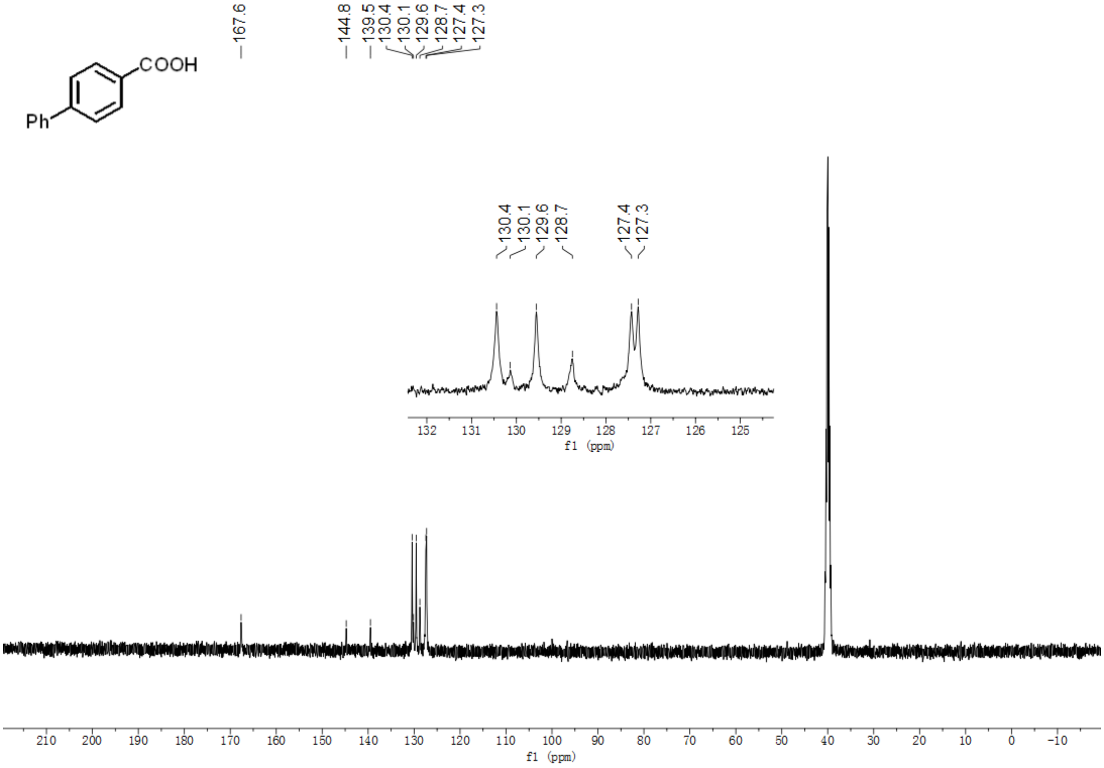

Supplementary Figure 5. ^{13}C NMR spectra of compound **2a&4b&4u**

Benzoic acid (2b&4a, *CDCl₃* as solvent)

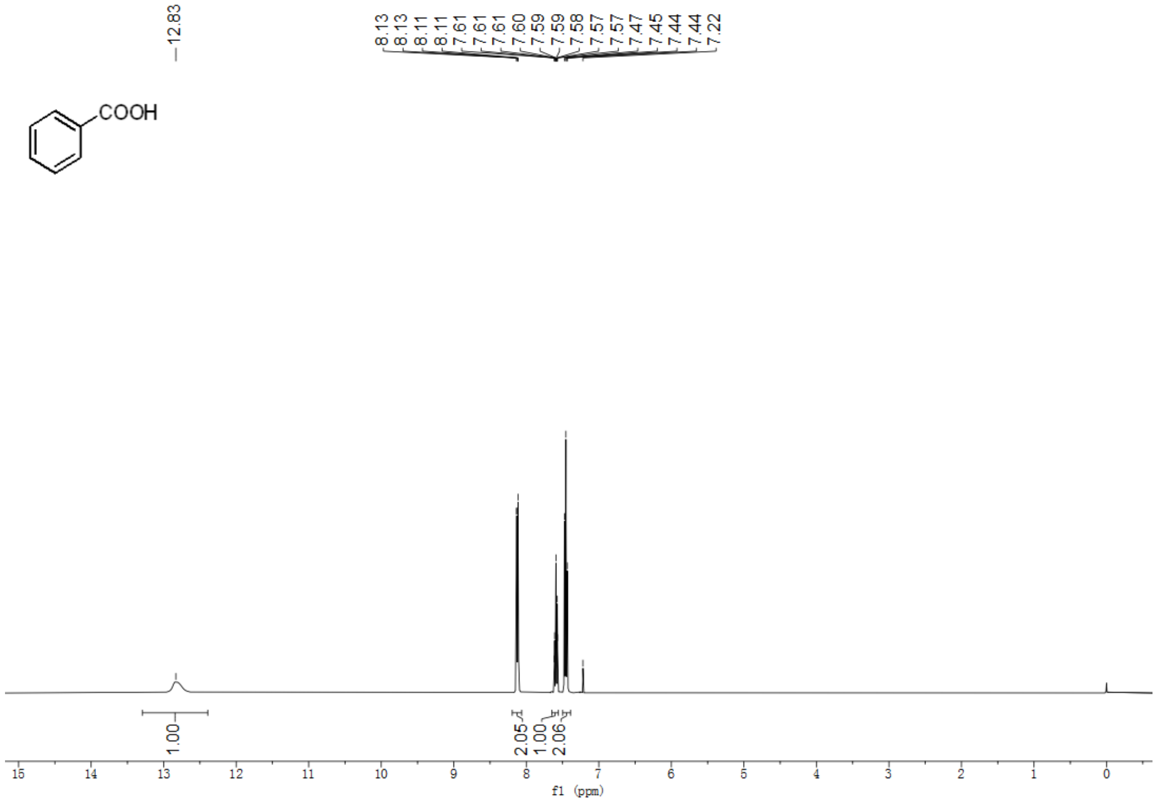

Supplementary Figure 6. ^{1}H NMR spectra of compound **2b&4a**

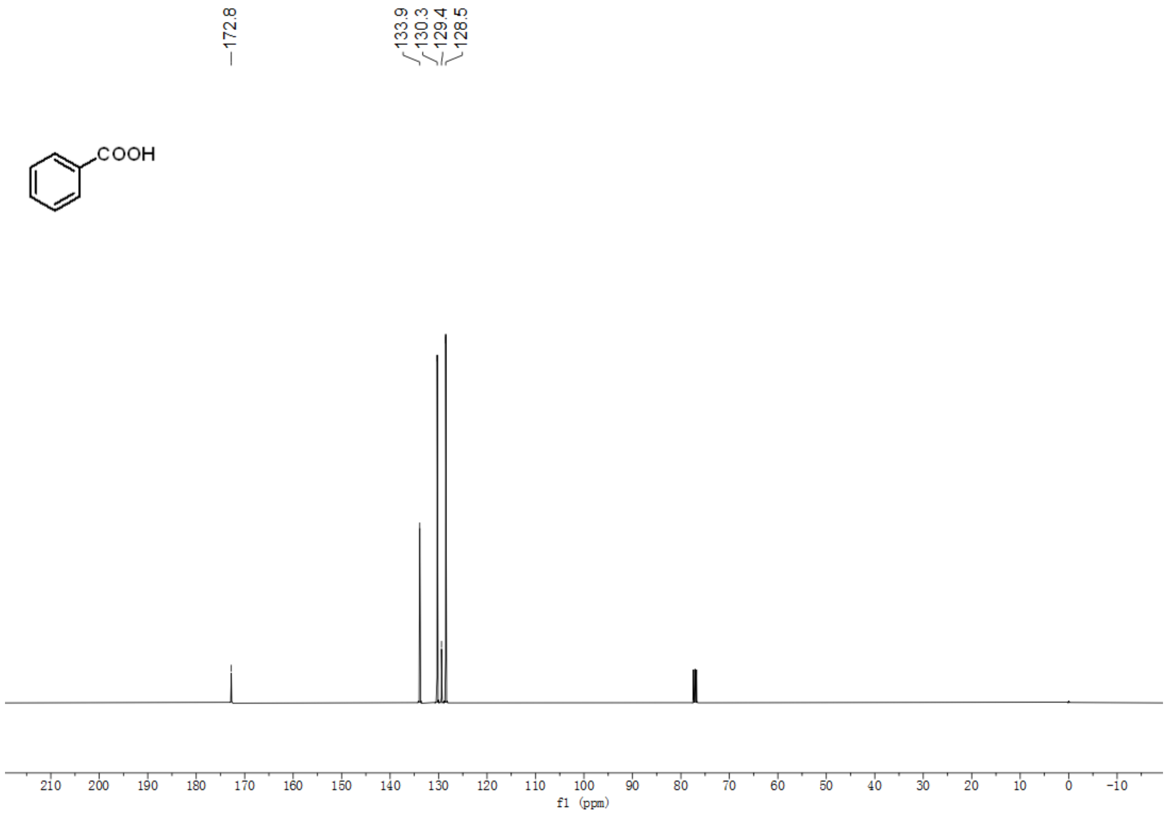

Supplementary Figure 7. ^{13}C NMR spectra of compound **2b&4a**

4-(hydroxy(phenyl)methyl)benzoic acid (2c, *DMSO-d_6* as solvent)

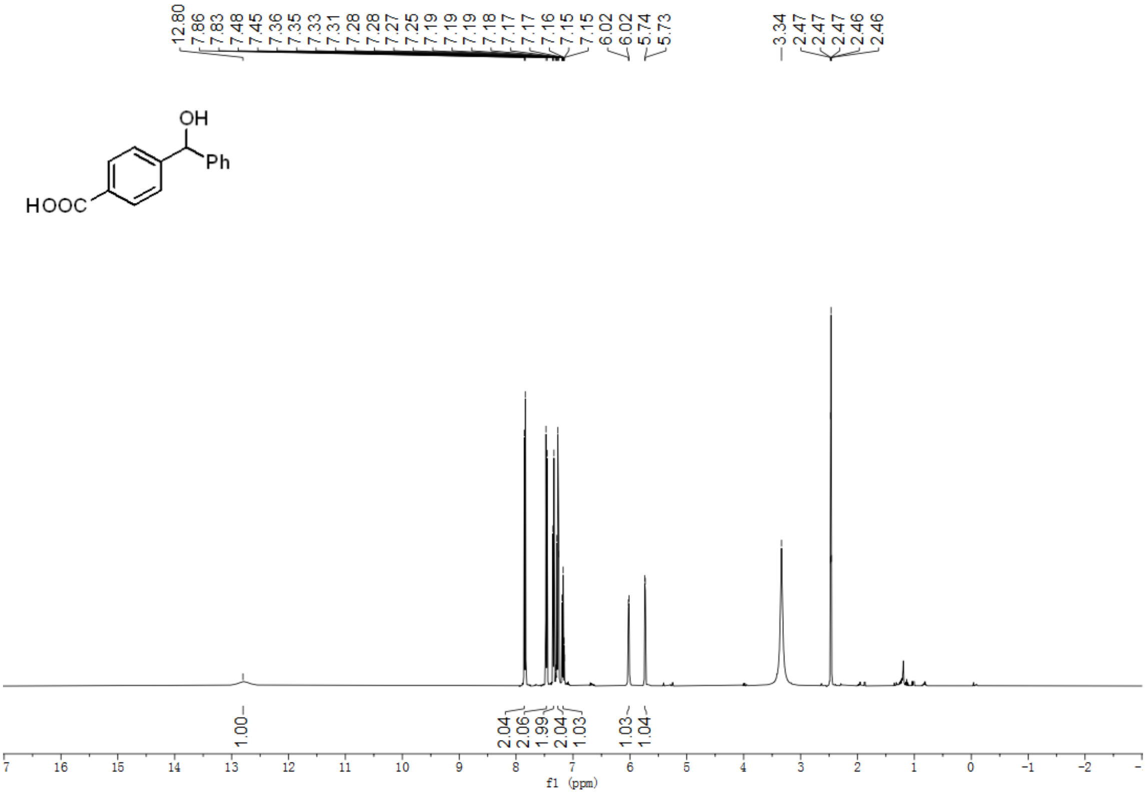

Supplementary Figure 8. ^{1}H NMR spectra of compound **2c**

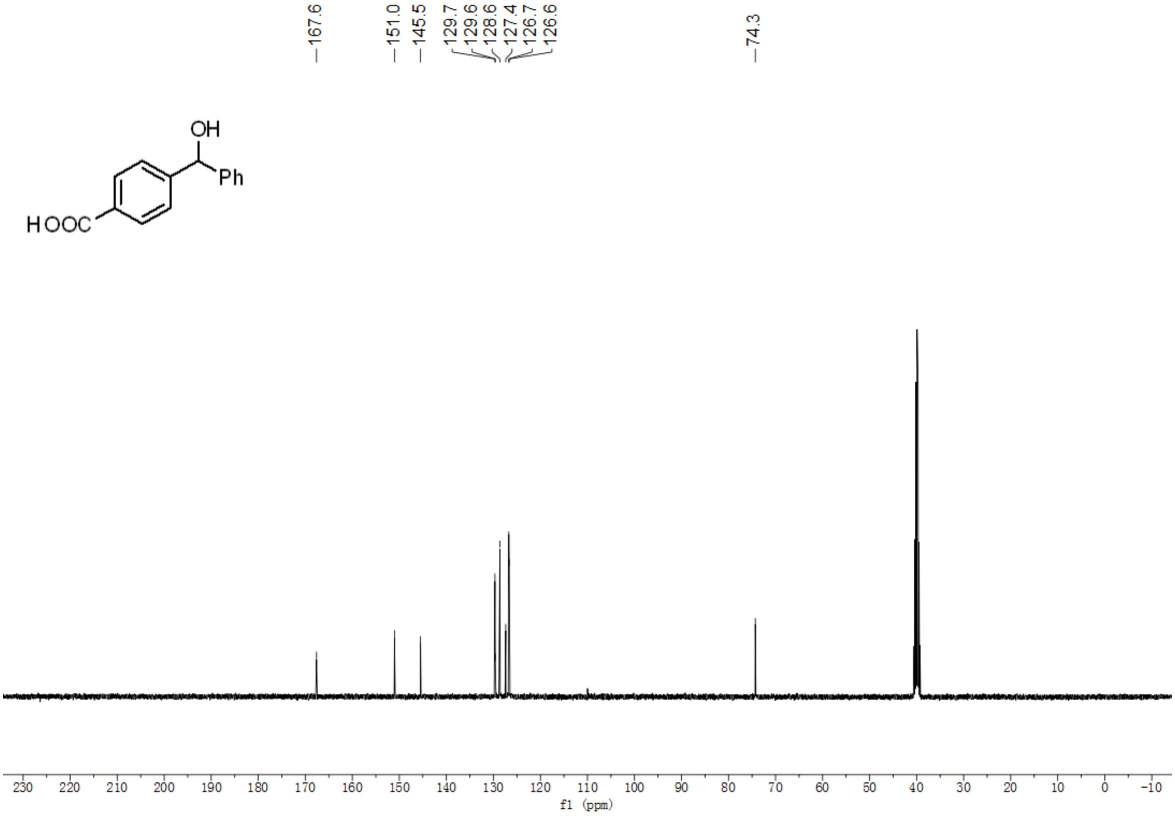

Supplementary Figure 9. ^{13}C NMR spectra of compound **2c**

4-fluorobenzoic acid (2d&4d, *$CDCl_3$* as solvent)

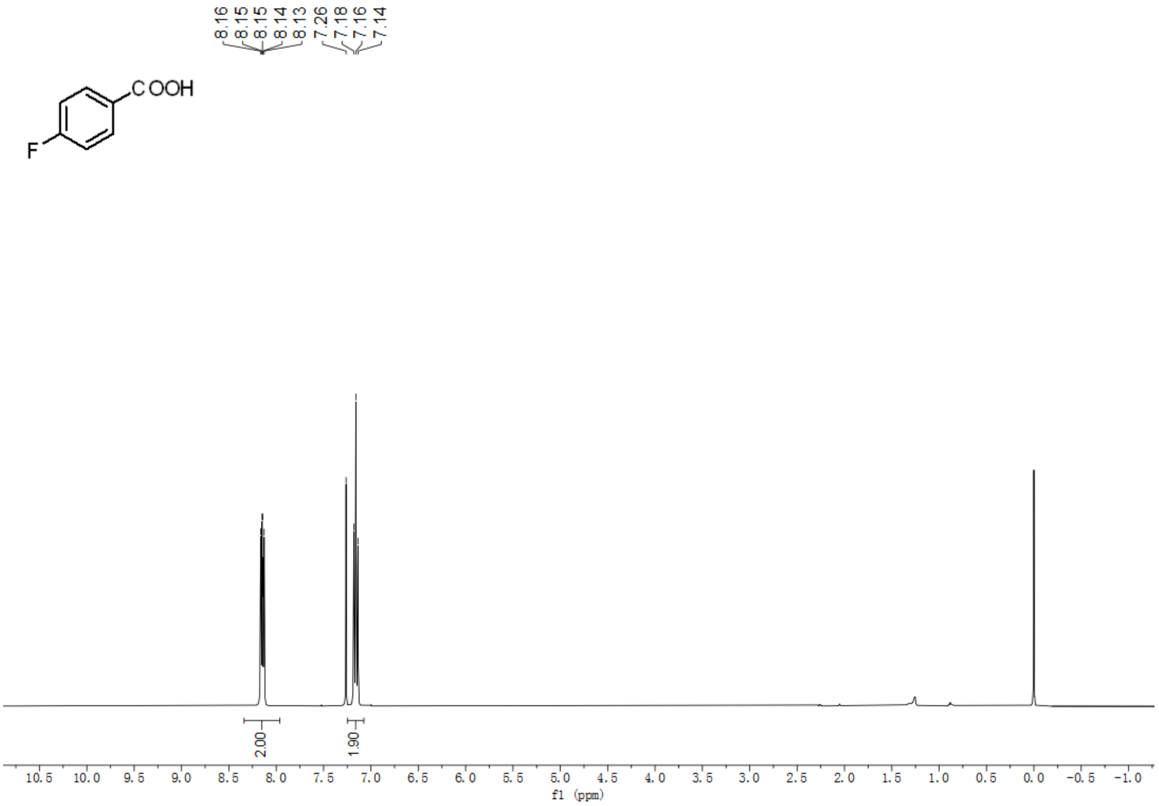

Supplementary Figure 10. ^{1}H NMR spectra of compound **2d&4d**

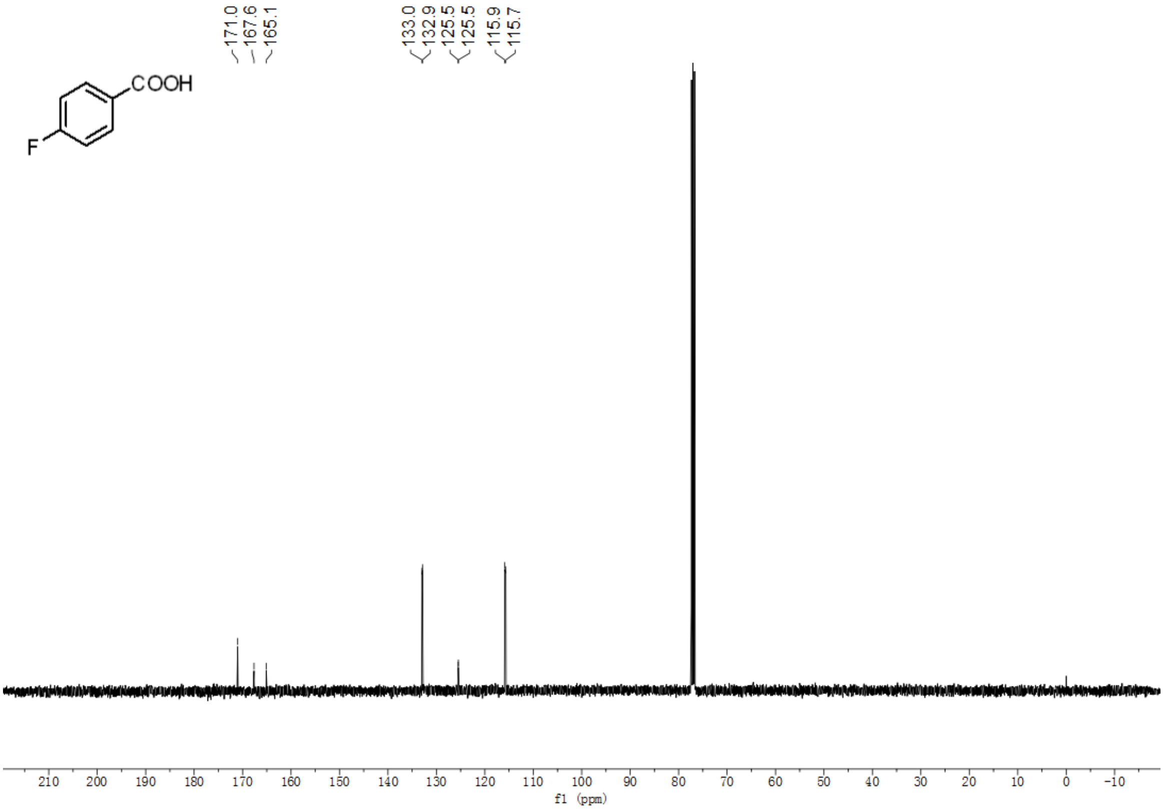

Supplementary Figure 11. ^{13}C NMR spectra of compound **2d&4d**

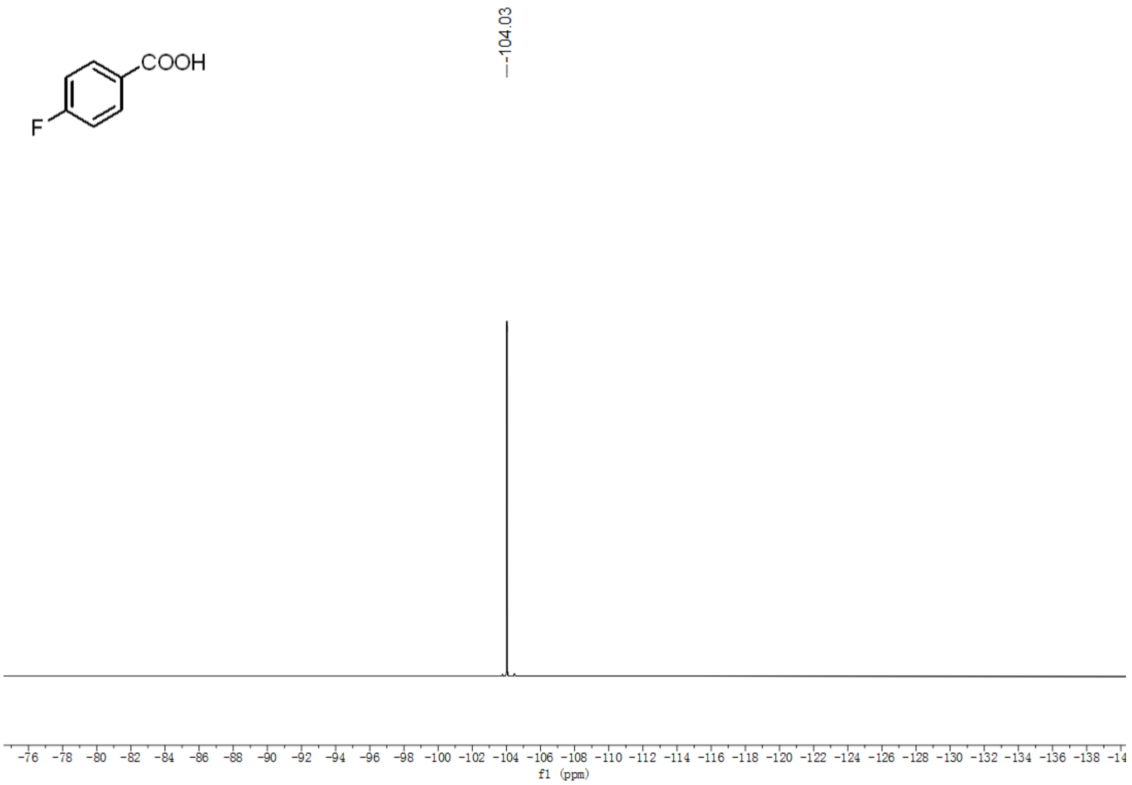

Supplementary Figure 12. ^{19}F NMR spectra of compound **2d&4d**

4-methoxybenzoic acid (2e&4c&4v&4z&4aa, *$CDCl_3$* as solvent)

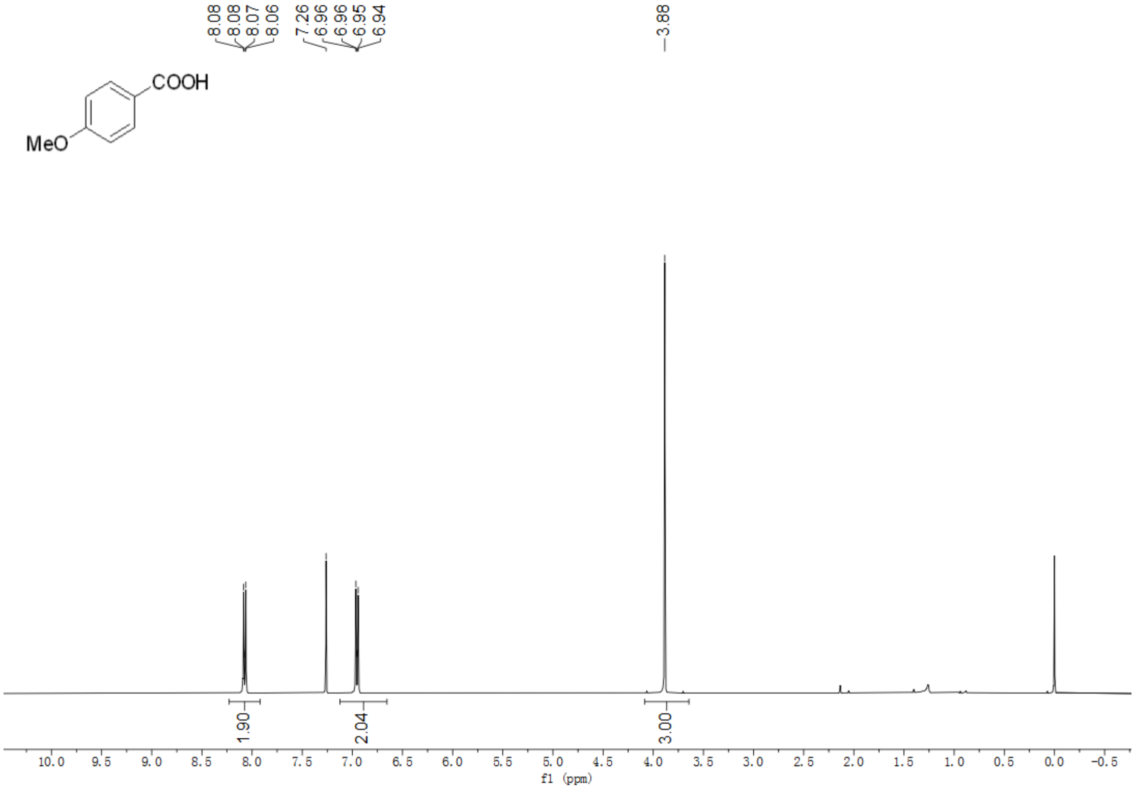

Supplementary Figure 13. ^{1}H NMR spectra of compound **2e&4c&4v&4z&4aa**

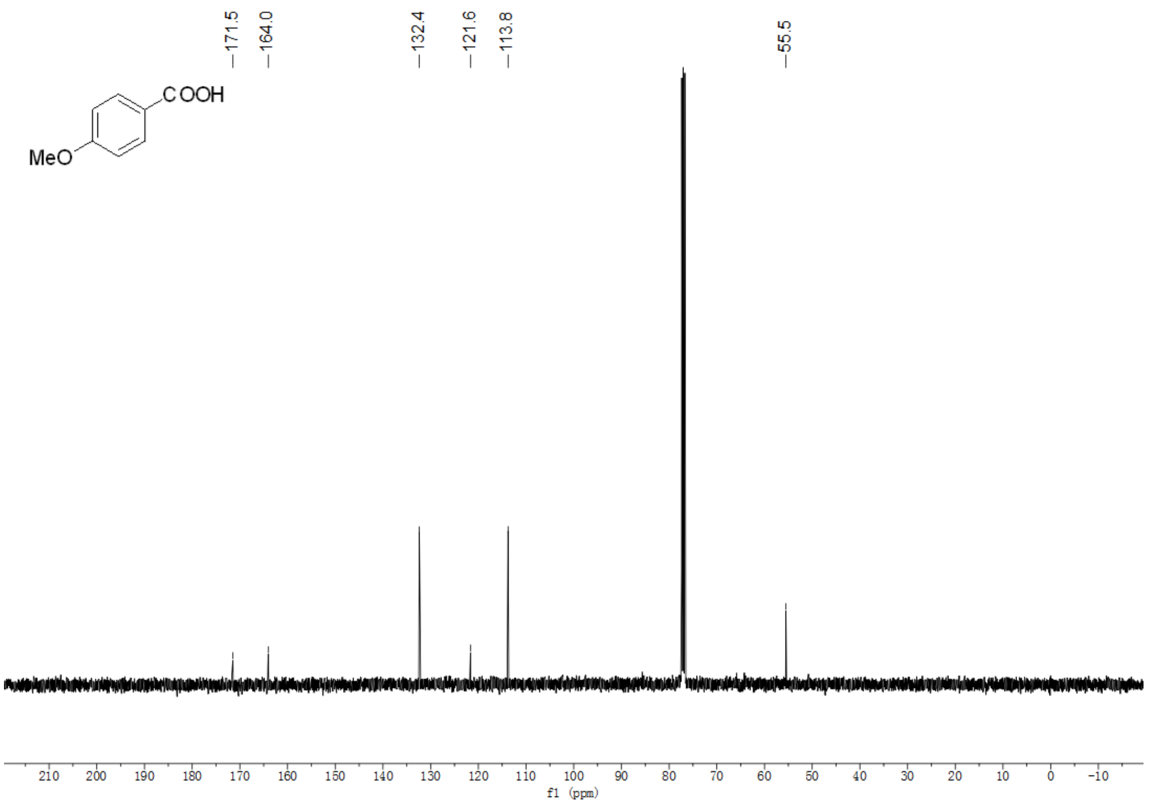

Supplementary Figure 14. ^{13}C NMR spectra of compound **2e&4c&4v&4z&4aa**

4-((1-ethoxy-2-methyl-1-oxopropan-2-yl)oxy)benzoic acid (2f, *$CDCl_3$* as solvent)

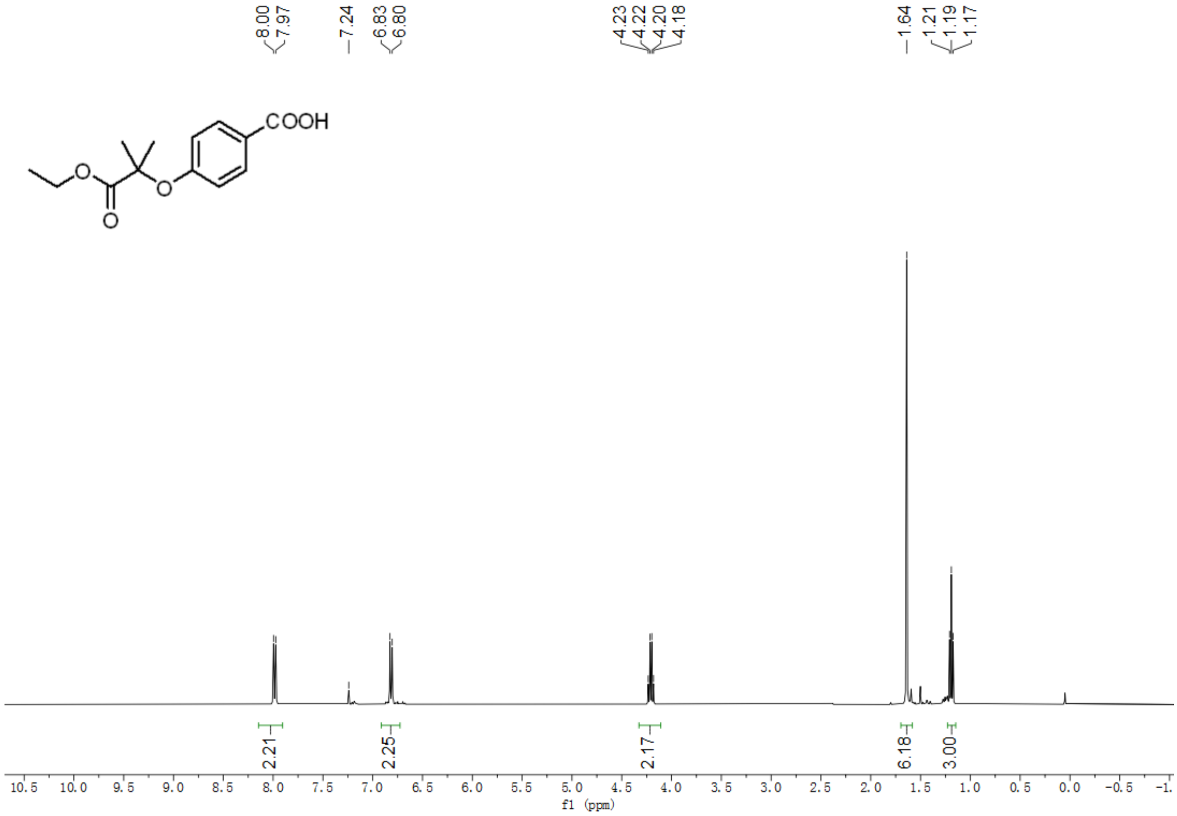

Supplementary Figure 15. ^{1}H NMR spectra of compound **2f**

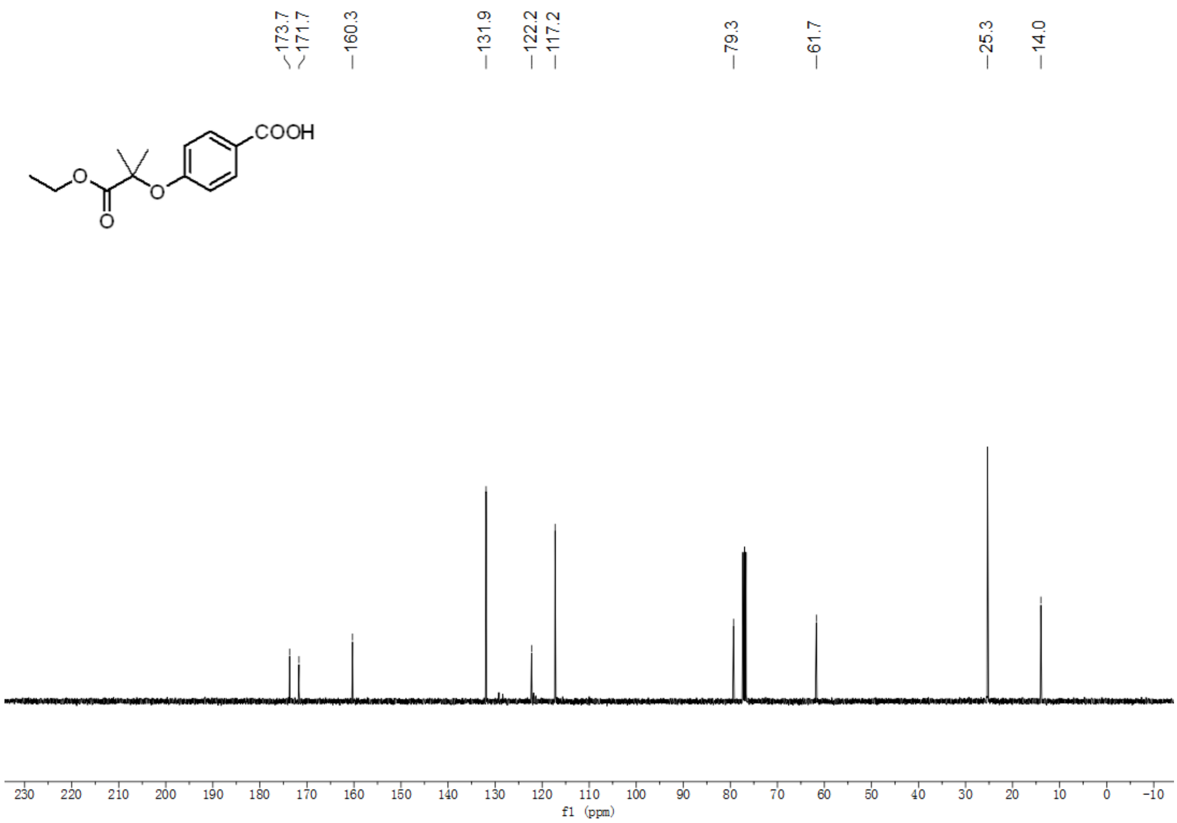

Supplementary Figure 16. ^{13}C NMR spectra of compound **2f**

4-(methoxycarbonyl)benzoic acid (2g, *DMSO-d_6* as solvent)

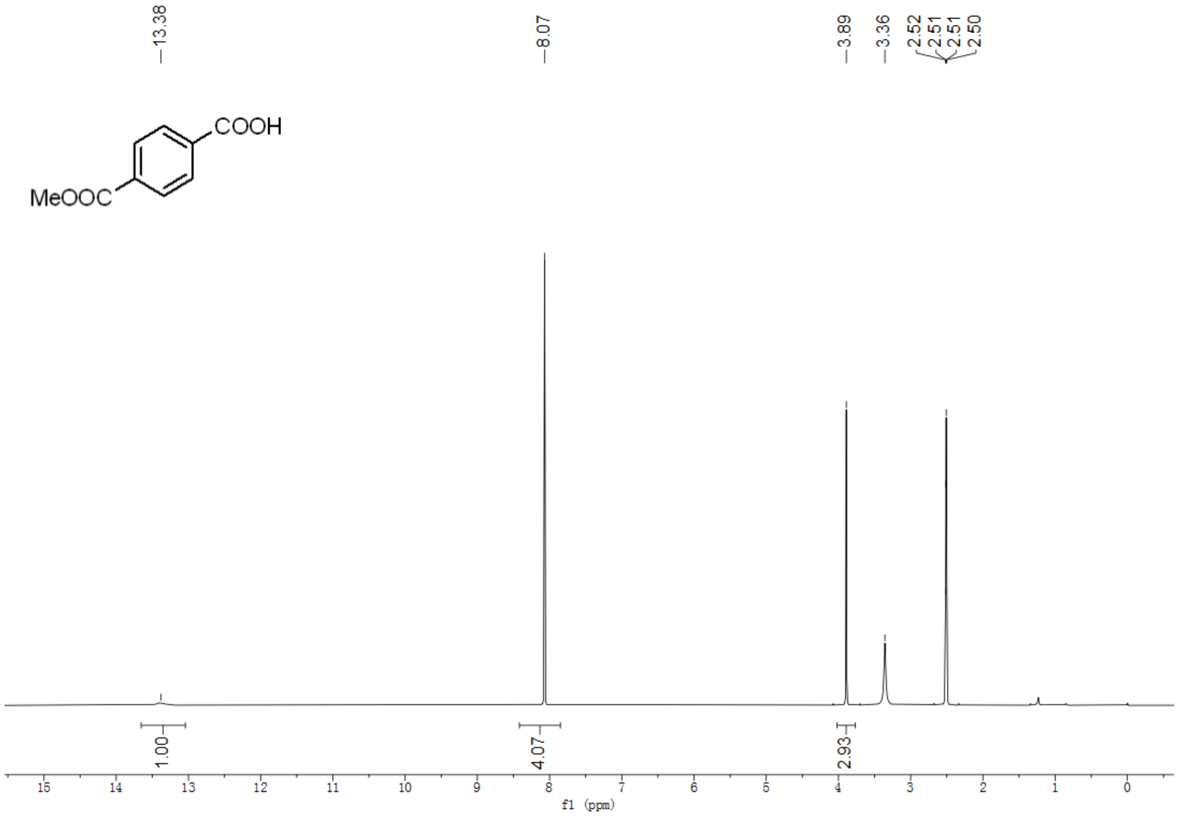

Supplementary Figure 17. ^{1}H NMR spectra of compound **2g**

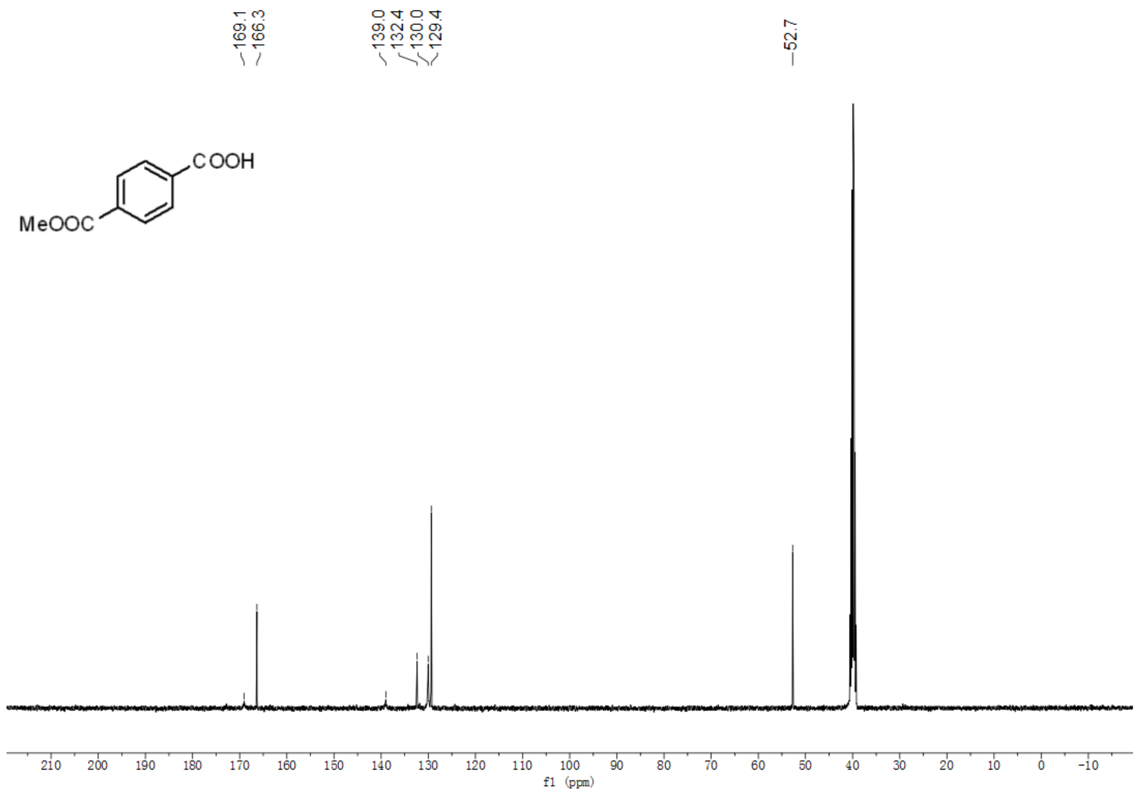

Supplementary Figure 18. ^{13}C NMR spectra of compound **2g**

4-acetylbenzoic acid (2h, *DMSO-d_6* as solvent)

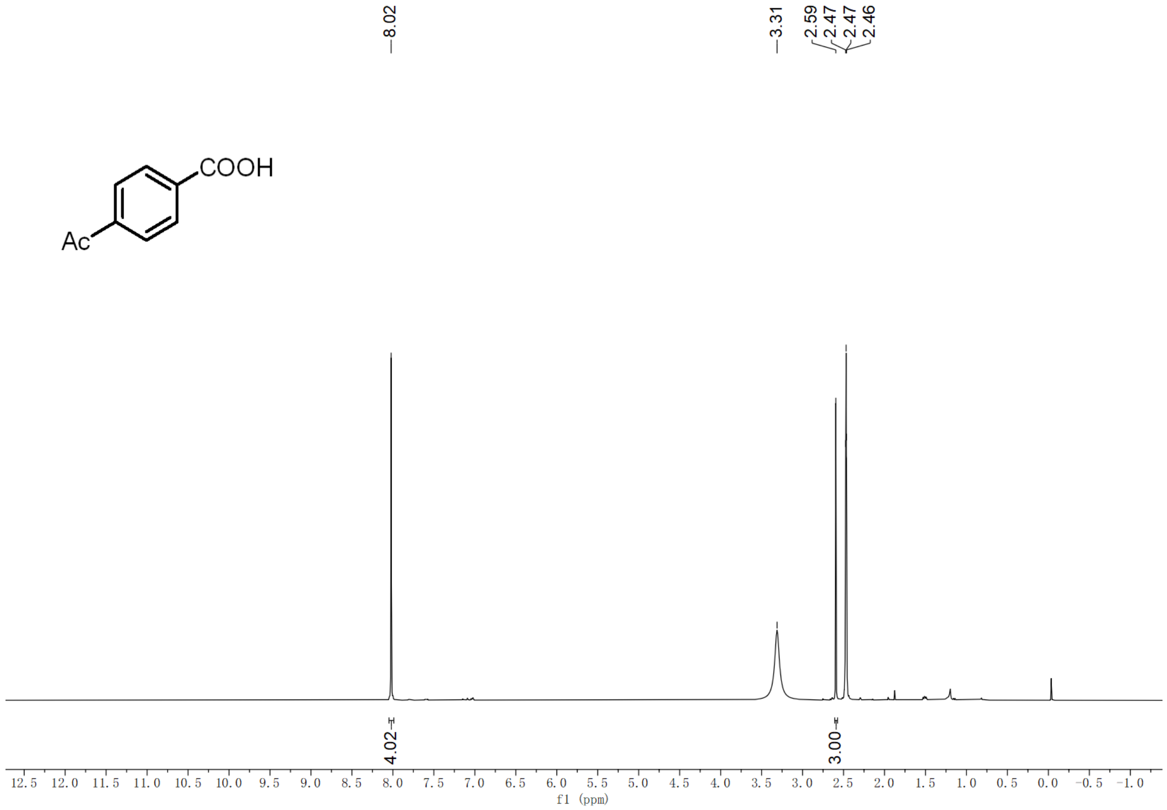

Supplementary Figure 19. ^{1}H NMR spectra of compound **2h**

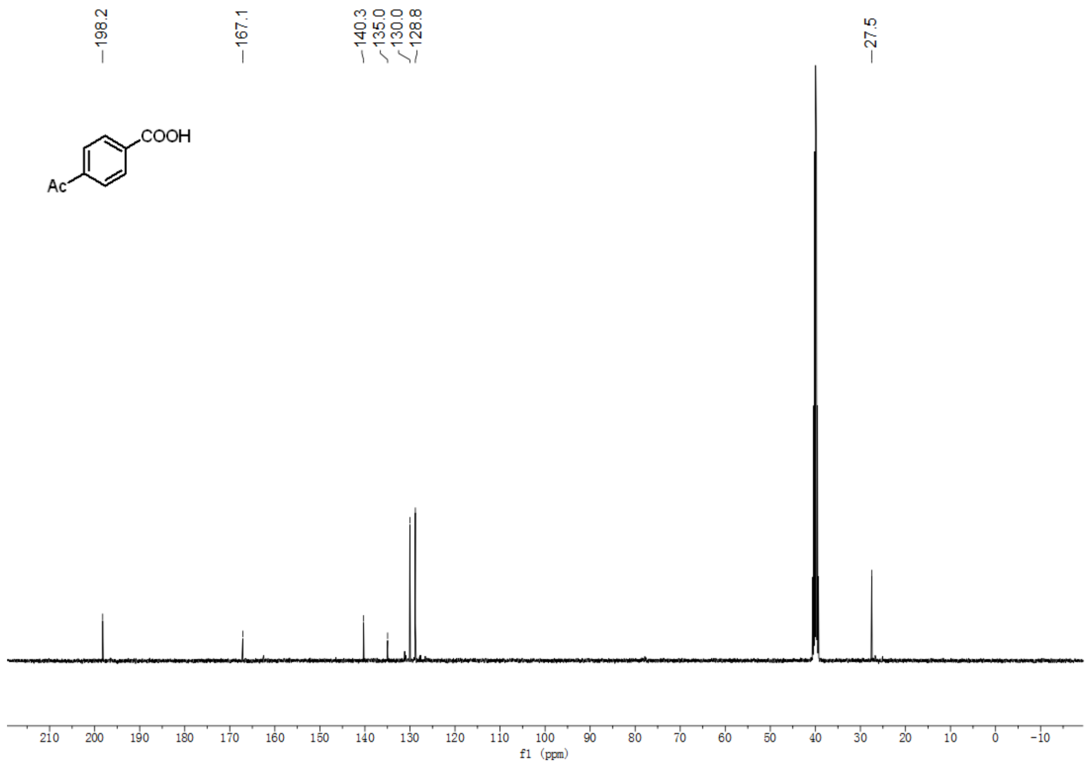

Supplementary Figure 20. ^{13}C NMR spectra of compound **2h**

3-(methoxycarbonyl)benzoic acid (2i, *DMSO-d_6* as solvent)

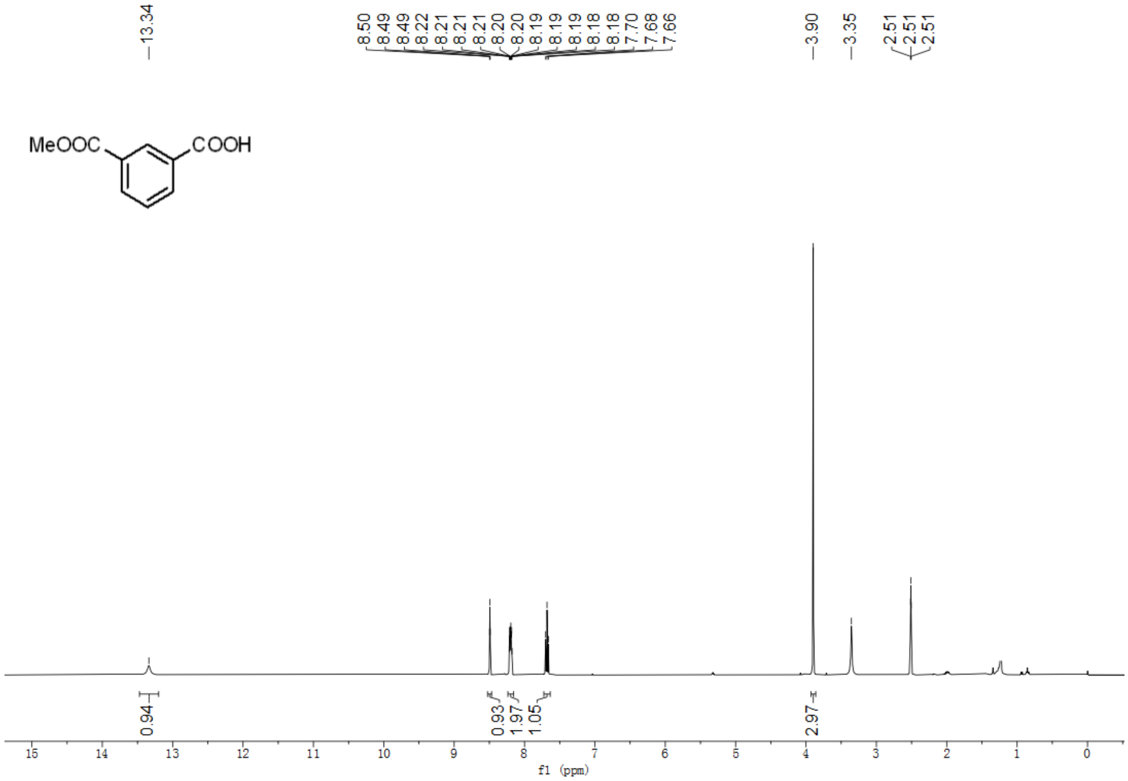

Supplementary Figure 21. ^{1}H NMR spectra of compound **2i**

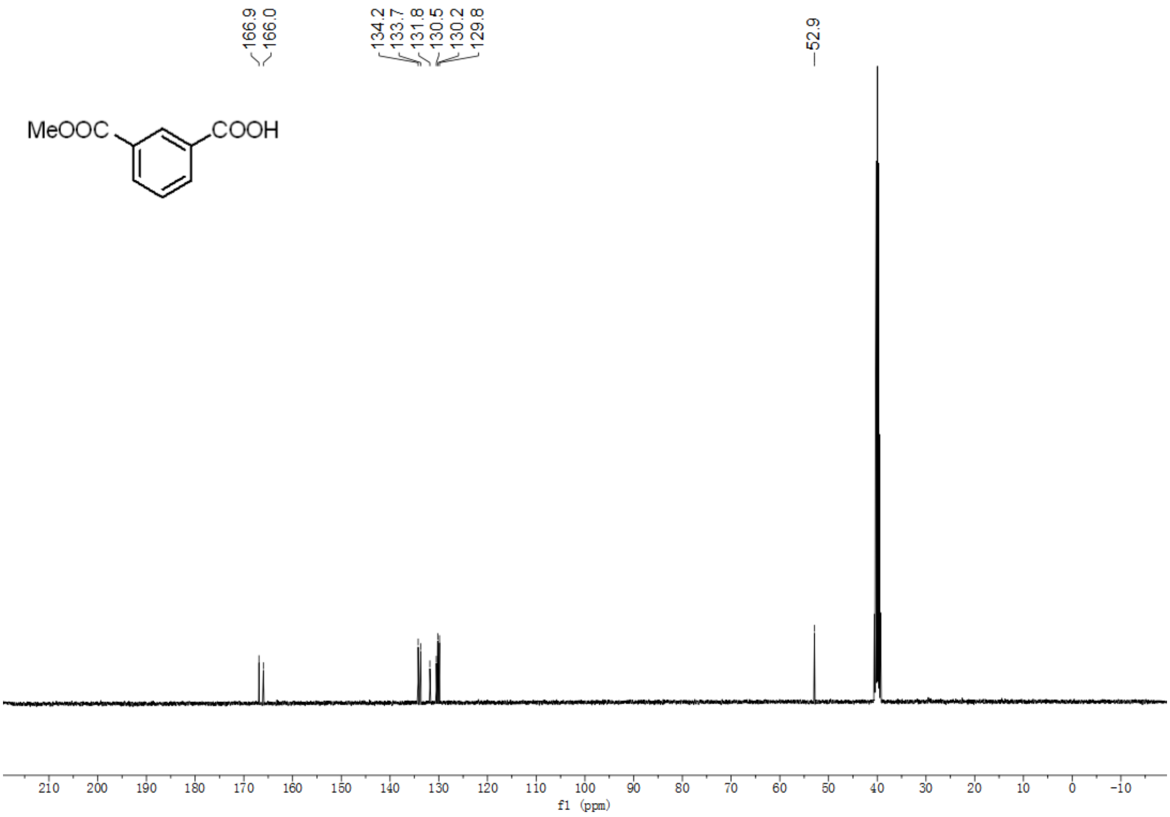

Supplementary Figure 22. ^{13}C NMR spectra of compound **2i**

3-acetylbenzoic acid (2j, *DMSO-d_6* as solvent)

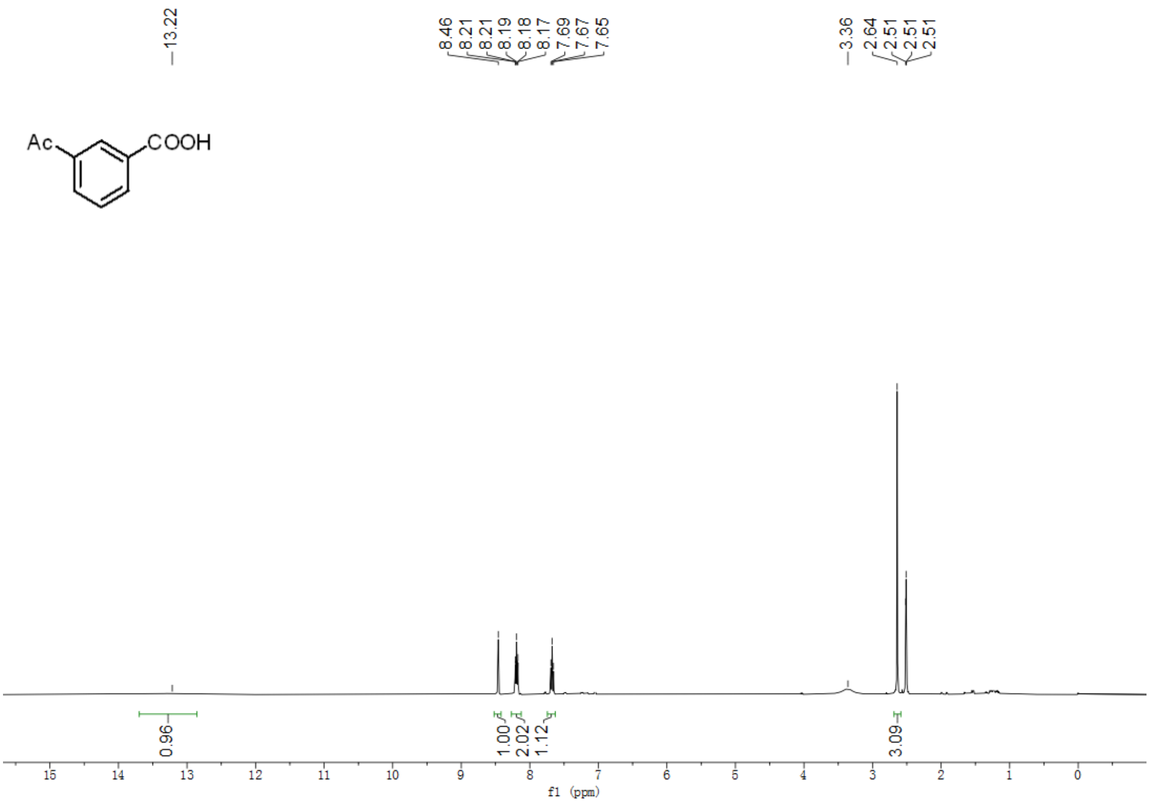

Supplementary Figure 23. ^{1}H NMR spectra of compound **2j**

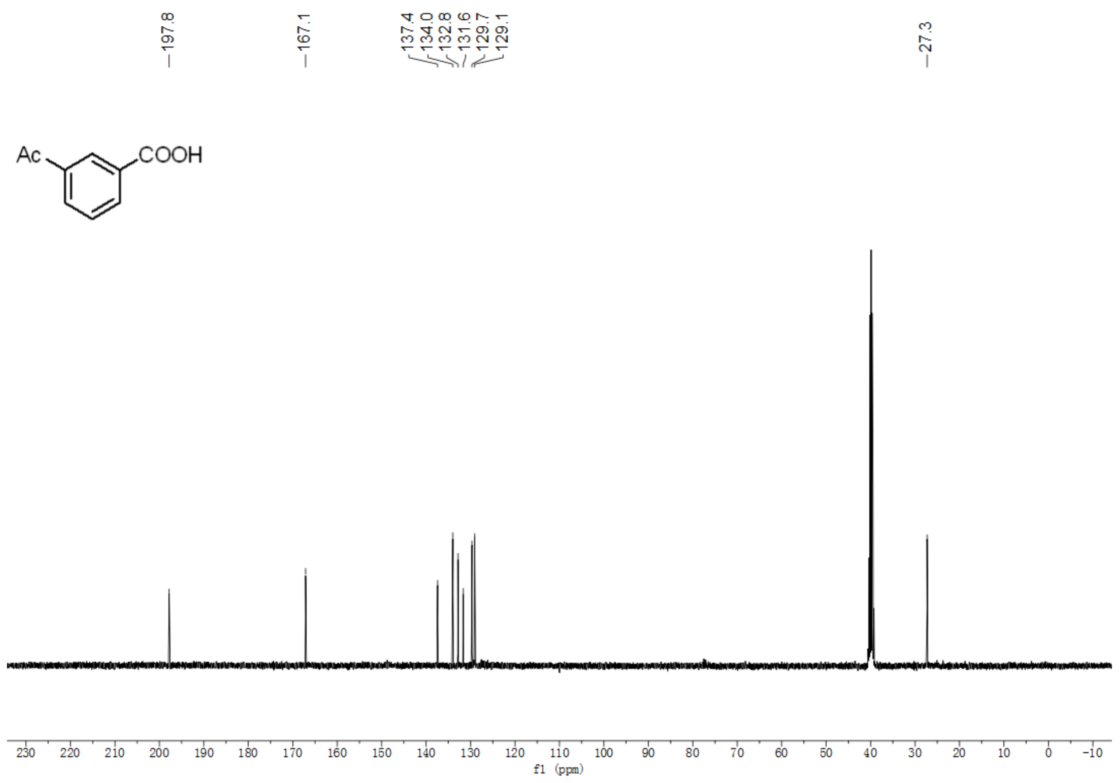

Supplementary Figure 24. ^{13}C NMR spectra of compound **2j**

2-naphthoic acid (2k&4x, *DMSO-d_6* as solvent)

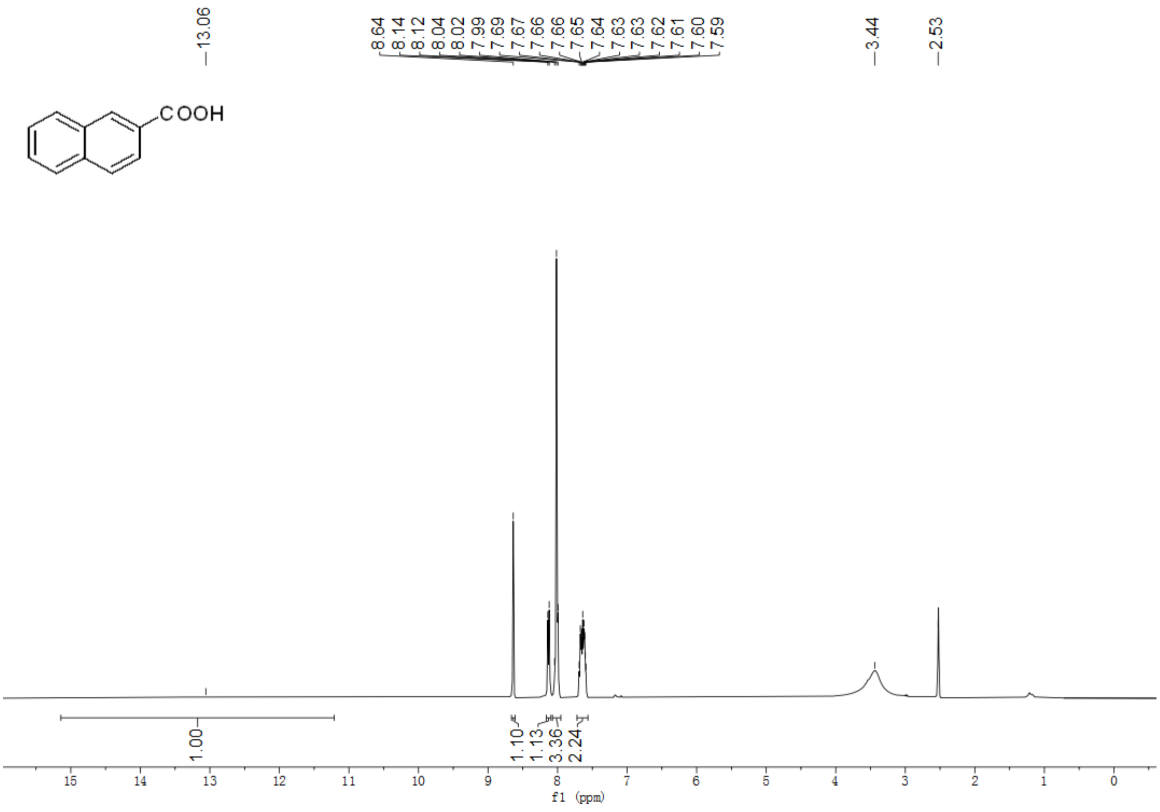

Supplementary Figure 25. ^{1}H NMR spectra of compound **2k&4x**

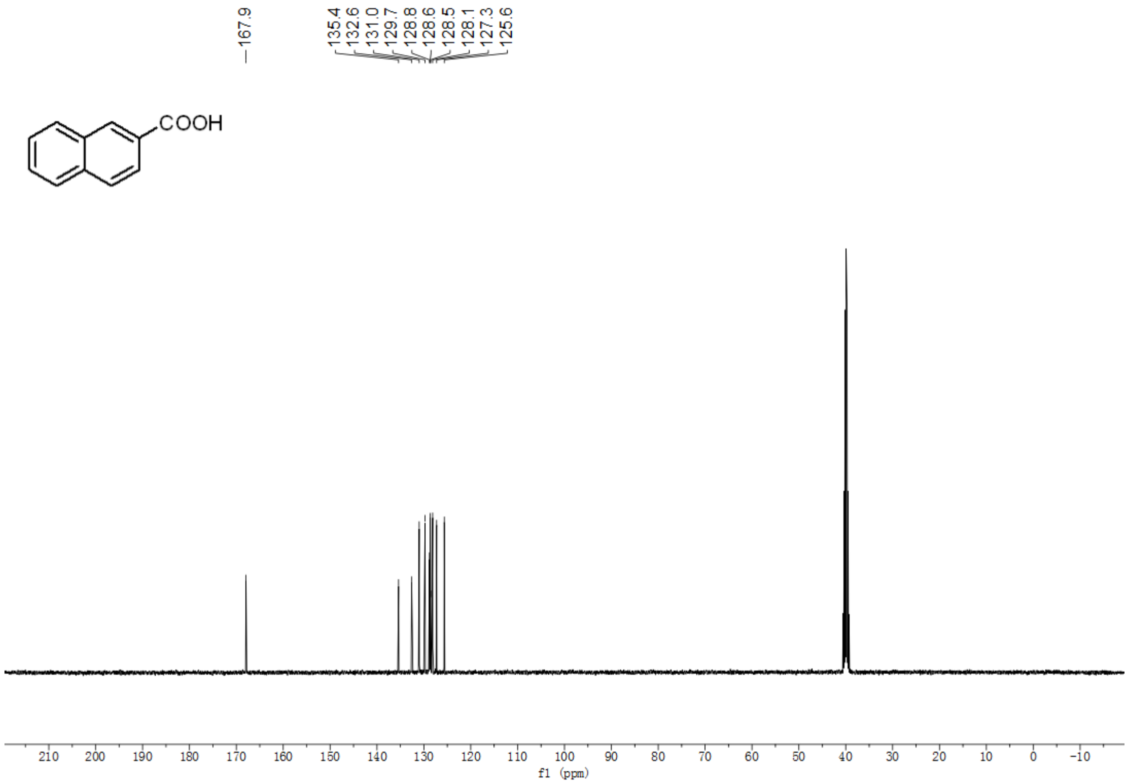

Supplementary Figure 26. ^{13}C NMR spectra of compound **2k&4x**

1-naphthoic acid (2l&4y, *DMSO-d_6* as solvent)

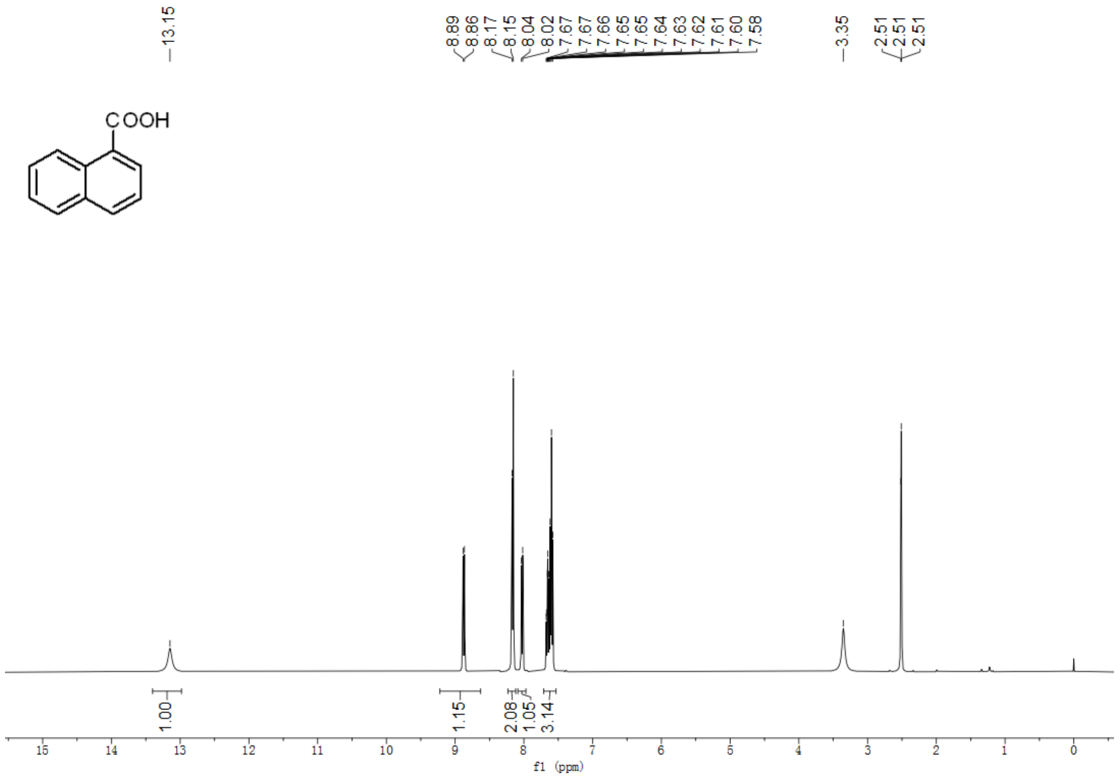

Supplementary Figure 27. ^{1}H NMR spectra of compound **2l&4y**

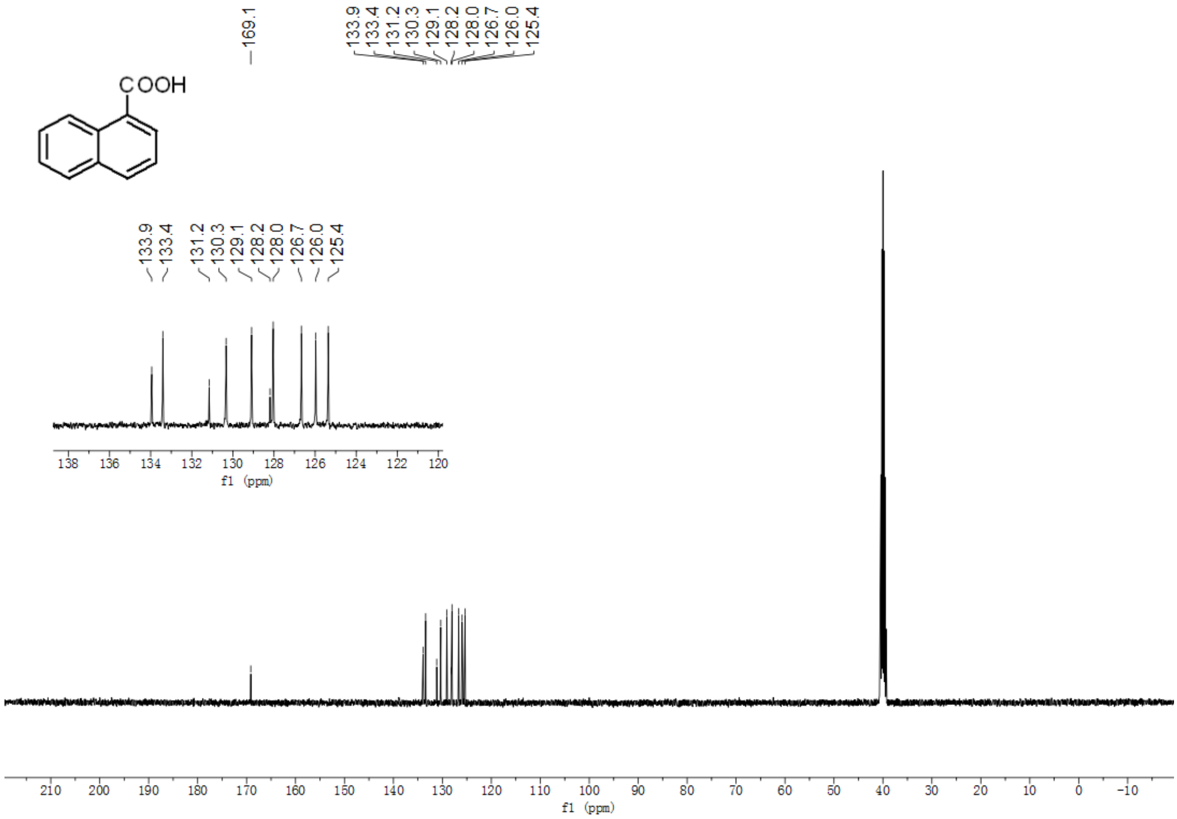

Supplementary Figure 28. ^{13}C NMR spectra of compound **2l&4y**

4-phenoxybenzoic acid (4d, *CDCl₃* as solvent)

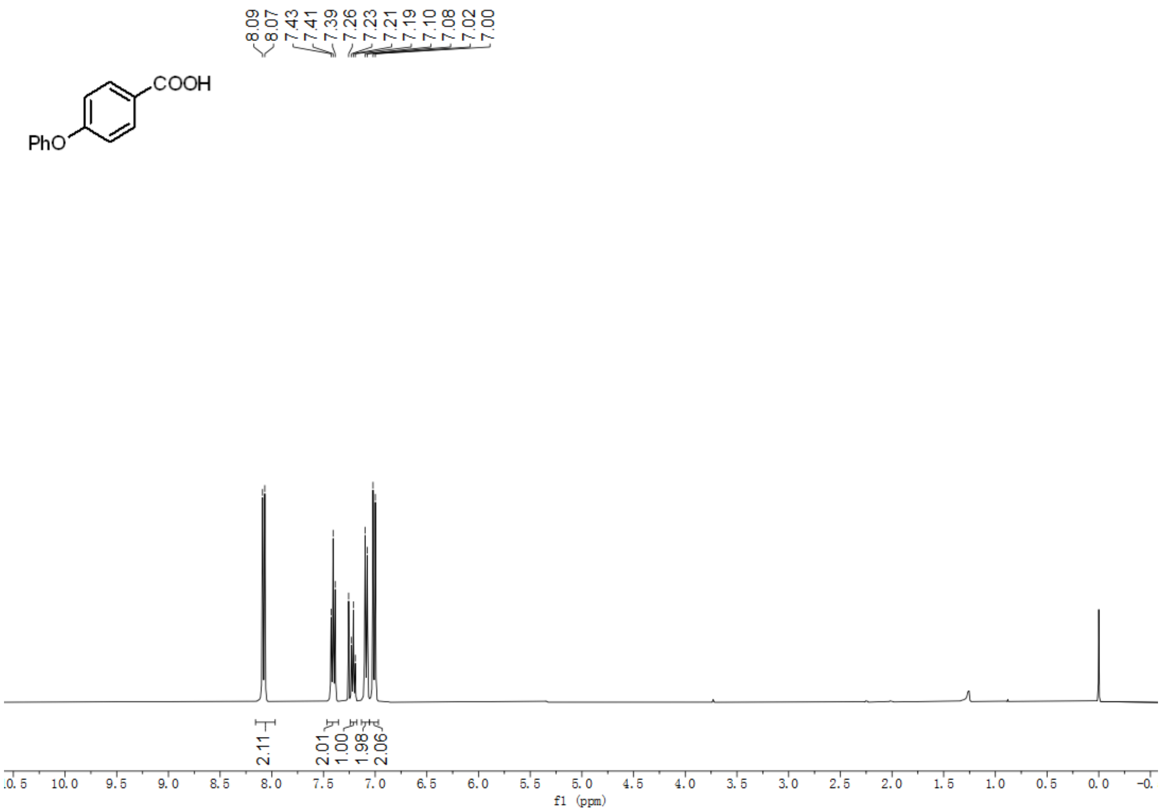

Supplementary Figure 29. ^{1}H NMR spectra of compound **4d**

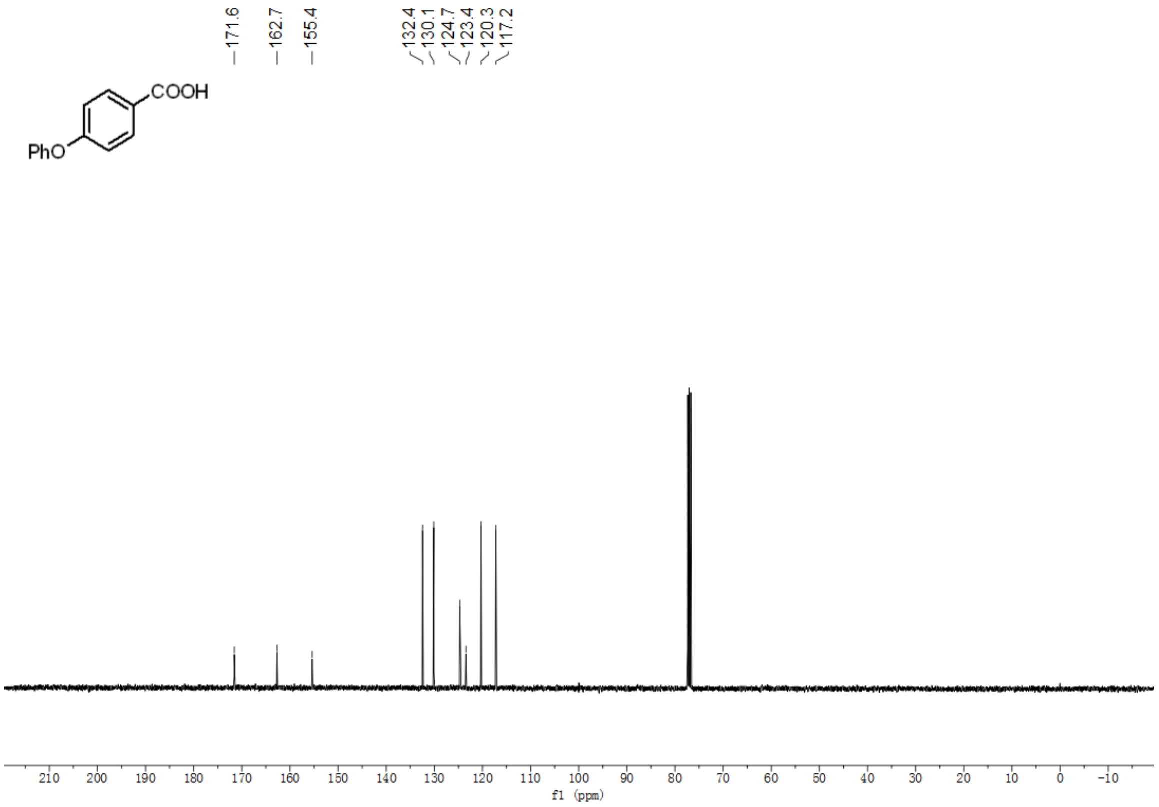

Supplementary Figure 30. ^{13}C NMR spectra of compound **4d**

4-(methylthio)benzoic acid (4e, *$CDCl_3$* as solvent)

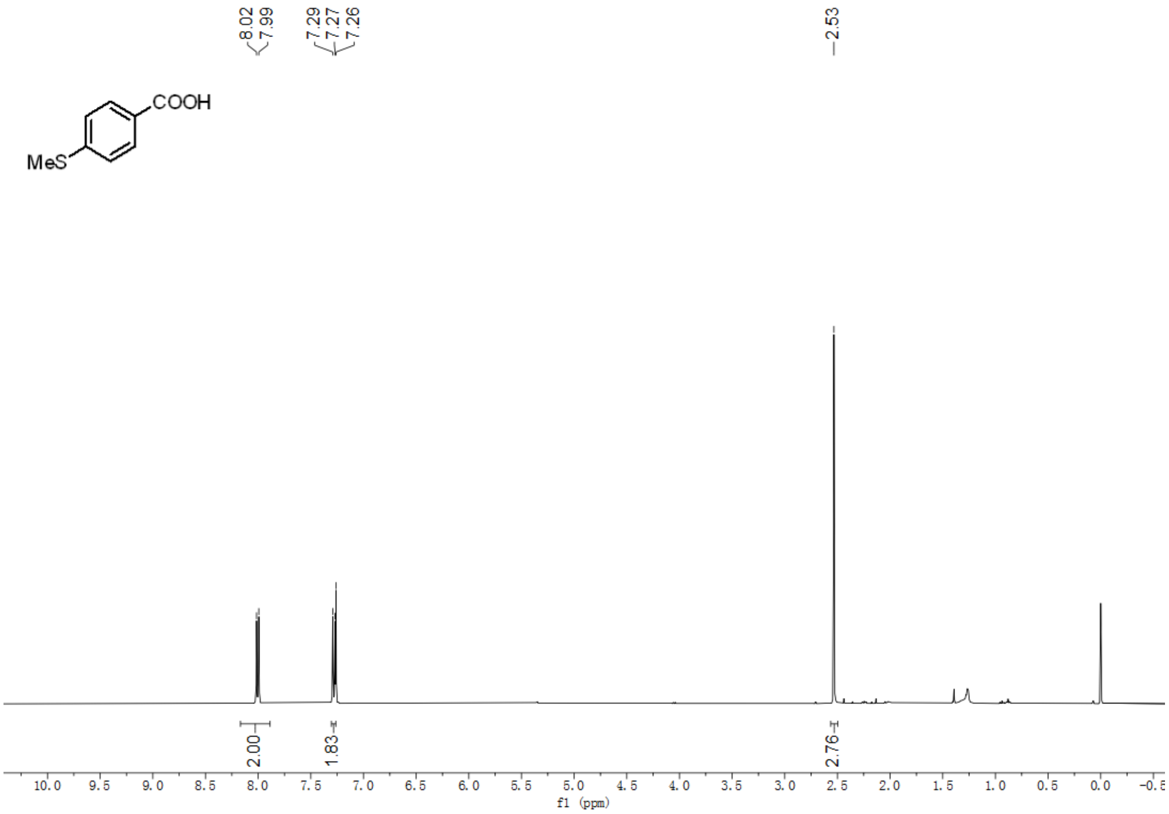

Supplementary Figure 31. ^{1}H NMR spectra of compound **4e**

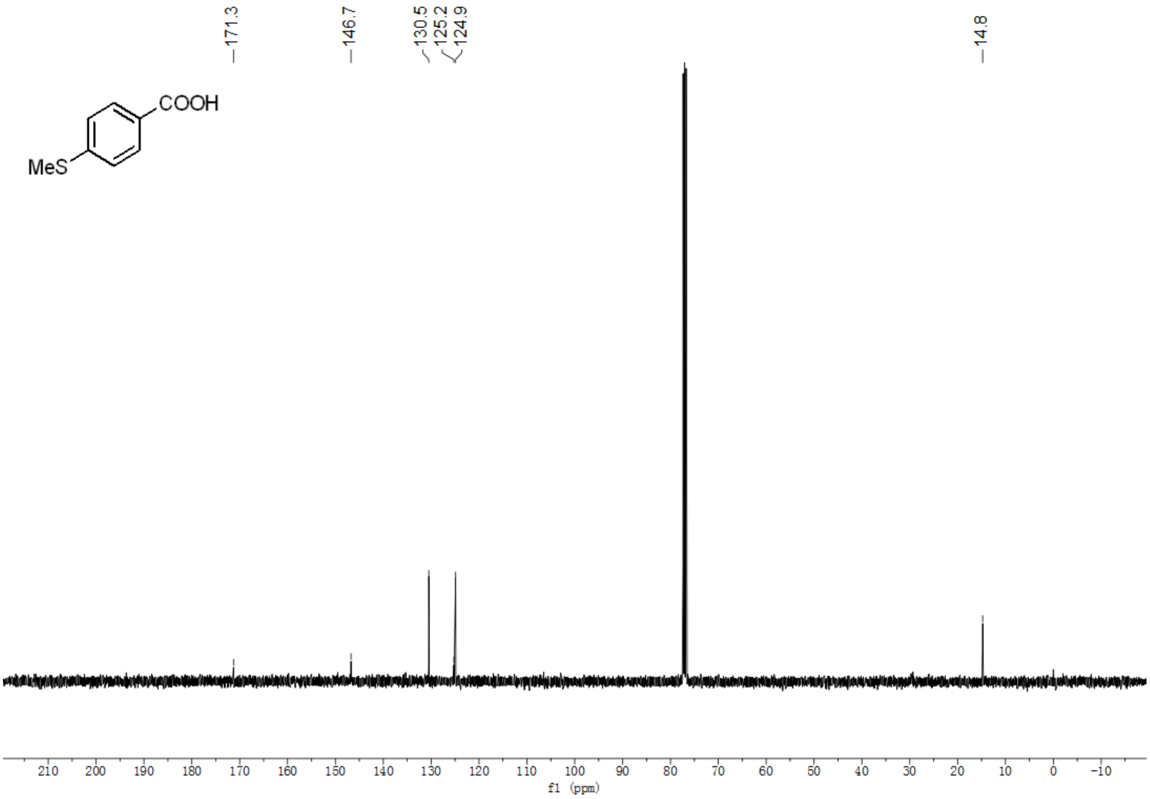

Supplementary Figure 32. ^{13}C NMR spectra of compound **4e**

4-(hydroxymethyl)benzoic acid (4f, *DMSO-d_6* as solvent)

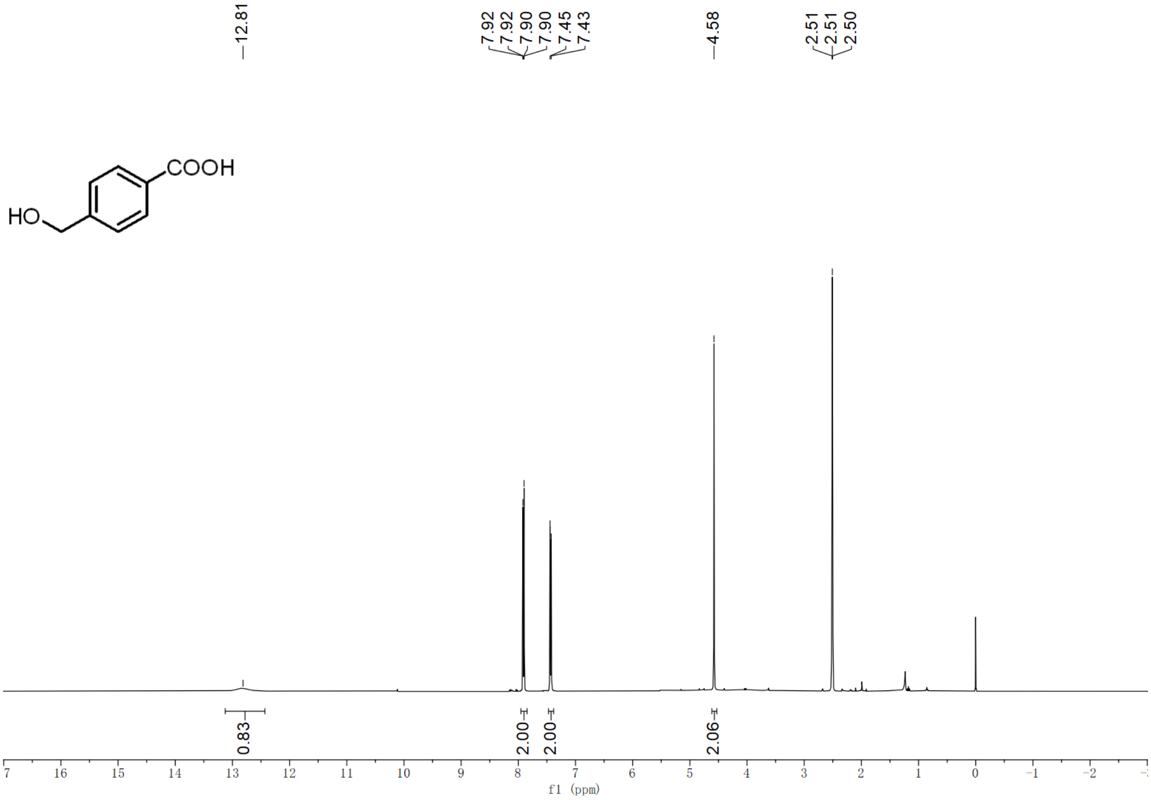

Supplementary Figure 33. ^{1}H NMR spectra of compound **4f**

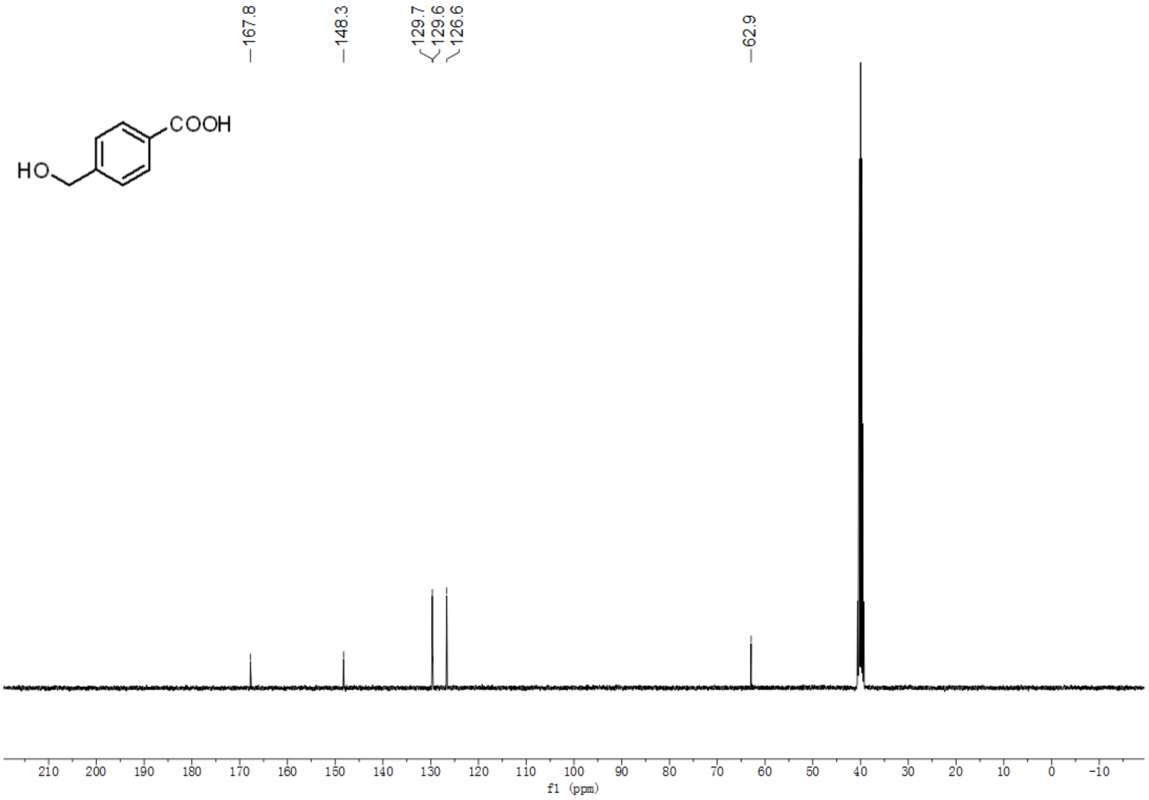

Supplementary Figure 34. ^{13}C NMR spectra of compound **4f**

4-(ethoxycarbonyl) benzoic acid (4h, *$CDCl_3$* as solvent)

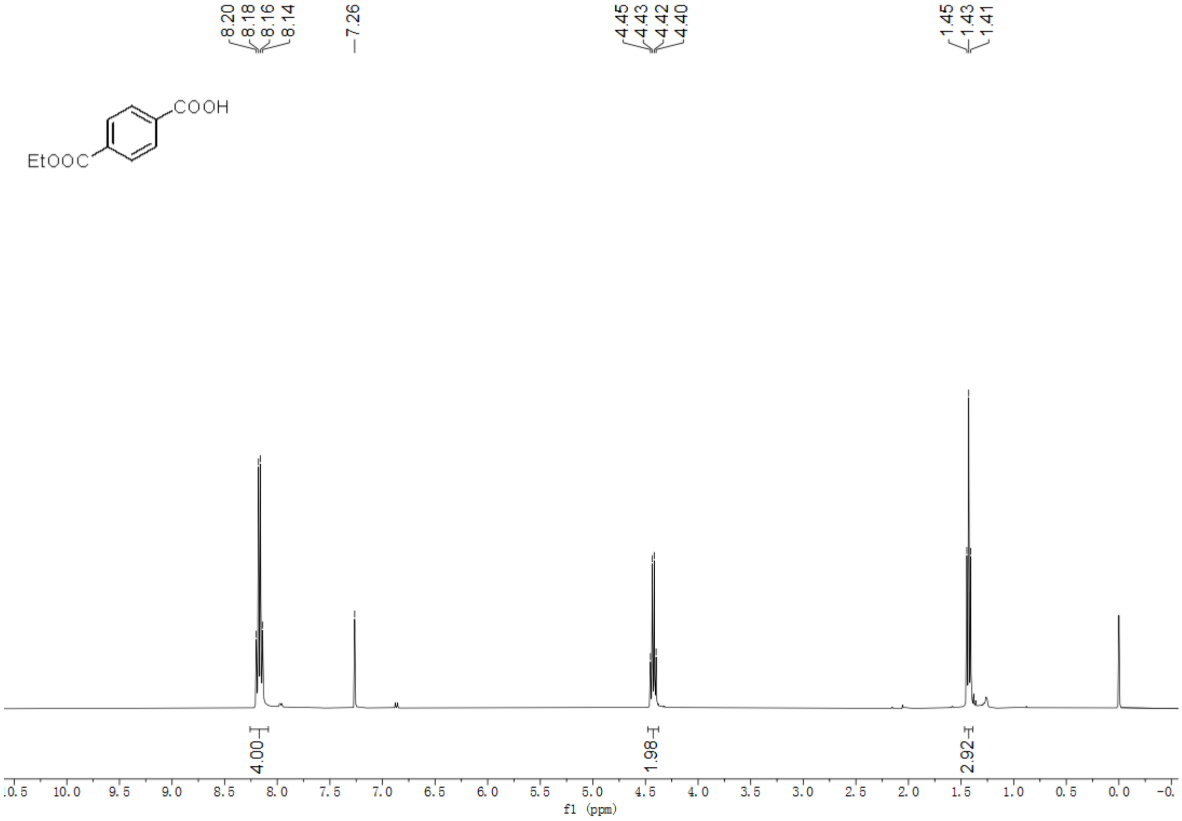

Supplementary Figure 35. ^{1}H NMR spectra of compound **4h**

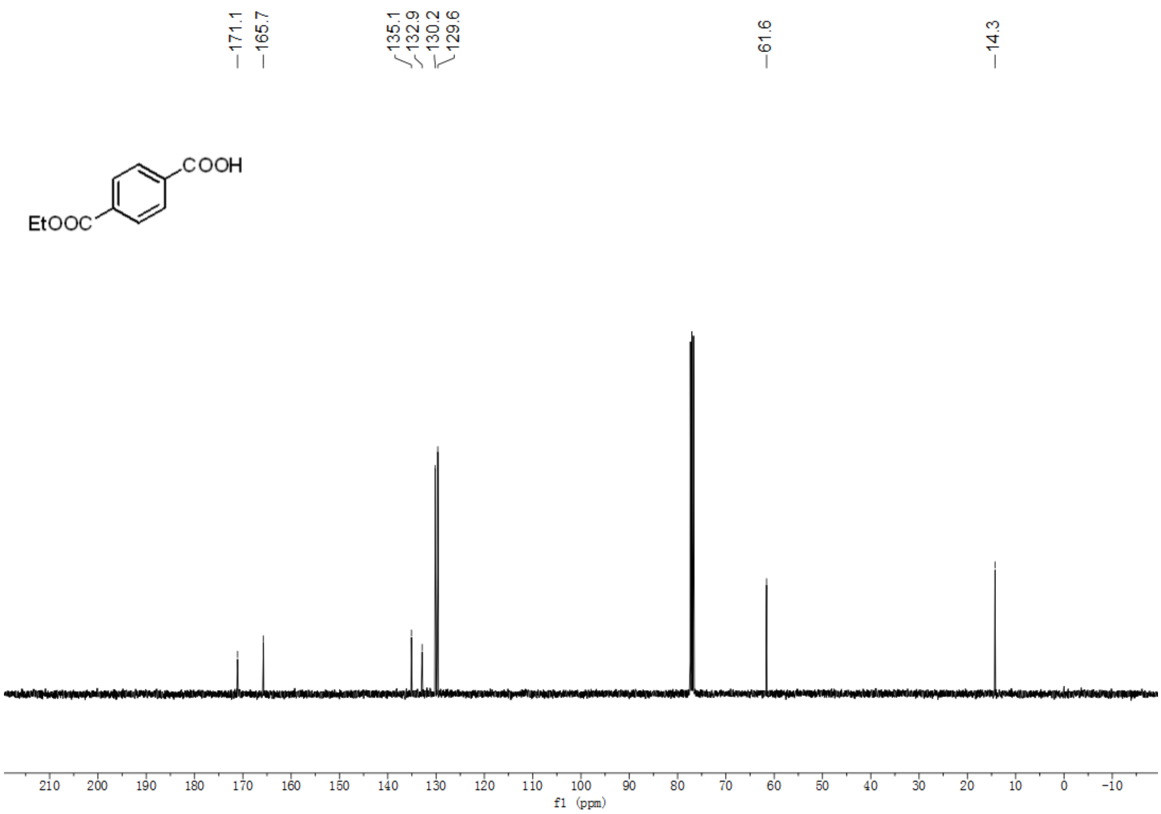

Supplementary Figure 36. ^{13}C NMR spectra of compound **4h**

3-methylbenzoic acid (4i&4w, *$CDCl_3$* as solvent)

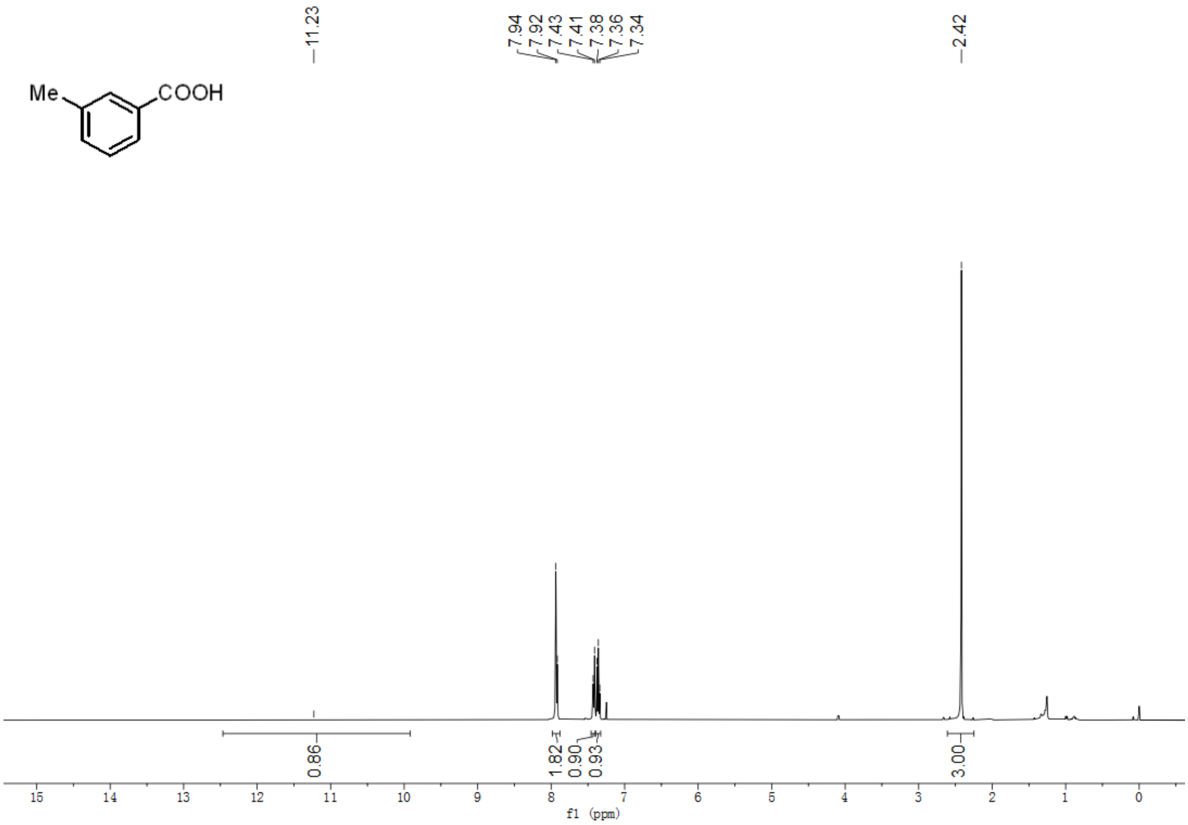

Supplementary Figure 37. ^{1}H NMR spectra of compound **4i&4w**

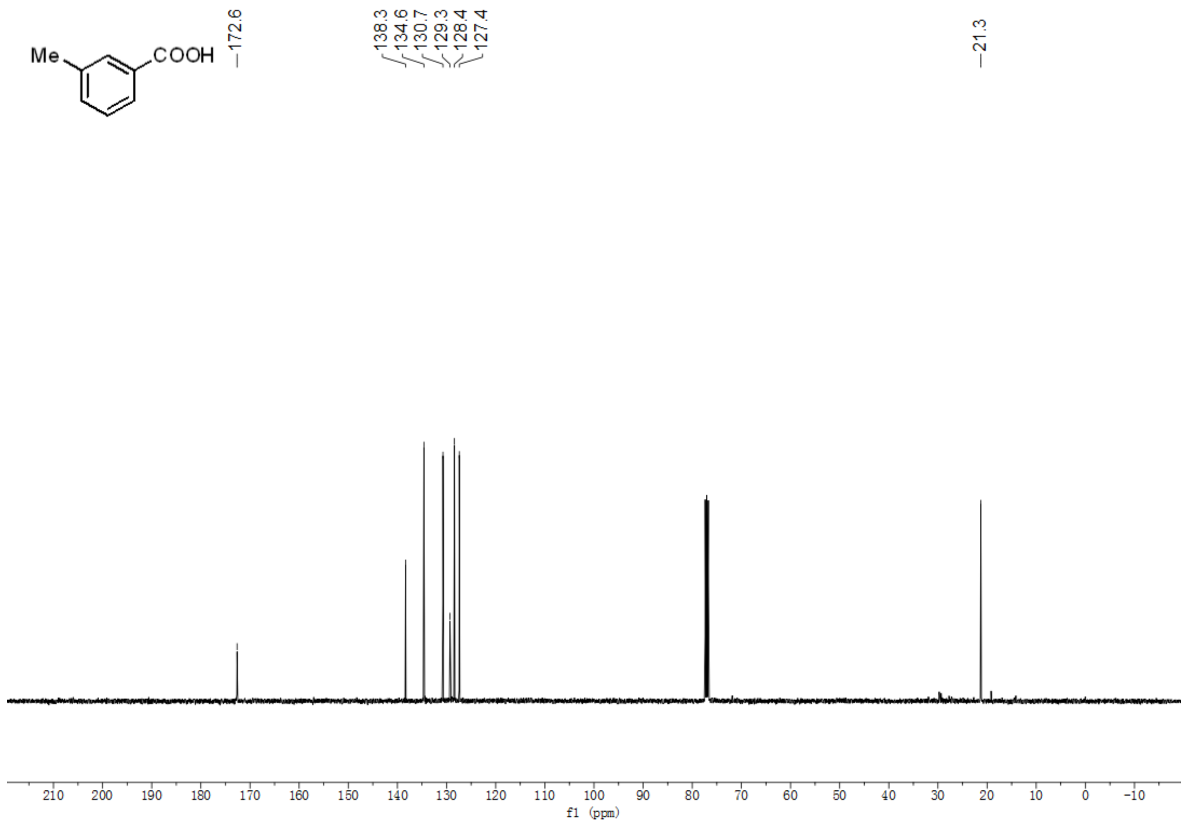

Supplementary Figure 38. ^{13}C NMR spectra of compound **4i&4w**

3-methoxybenzoic acid (4j, *CDCl₃* as solvent)

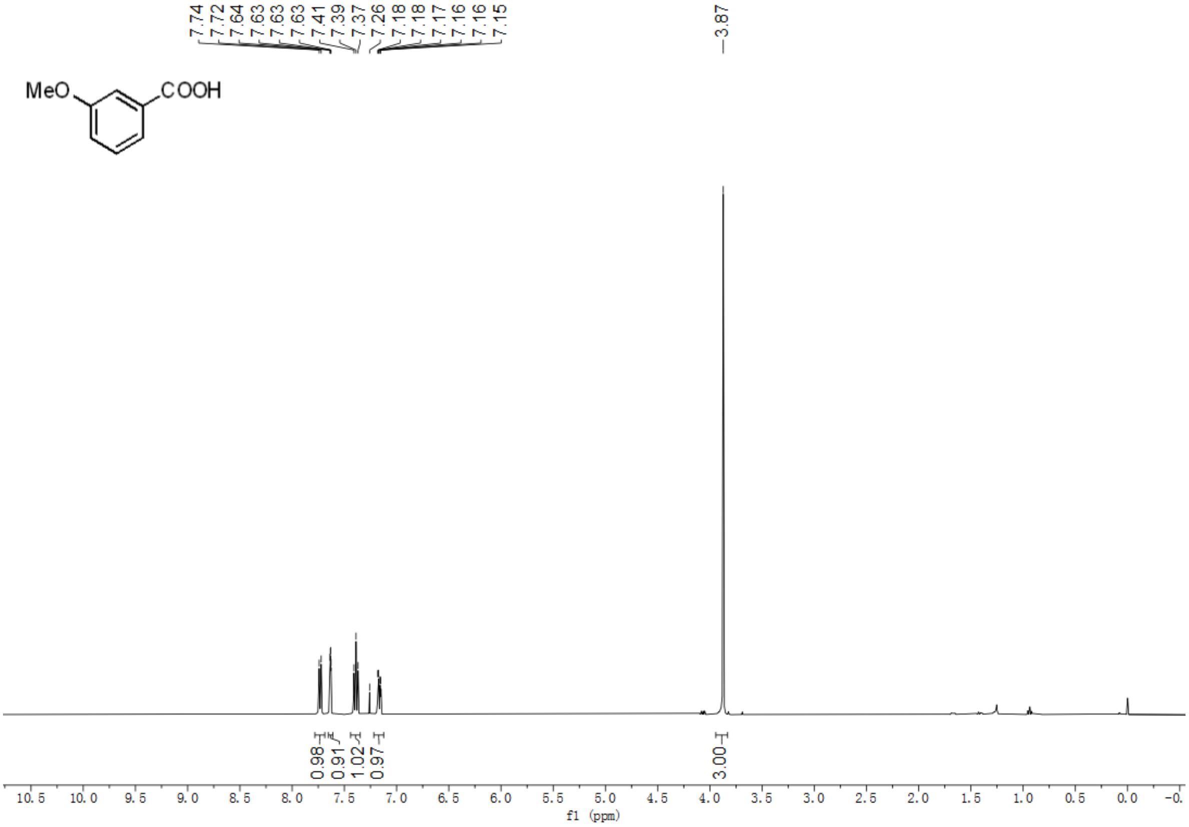

Supplementary Figure 39. ^{1}H NMR spectra of compound **4j**

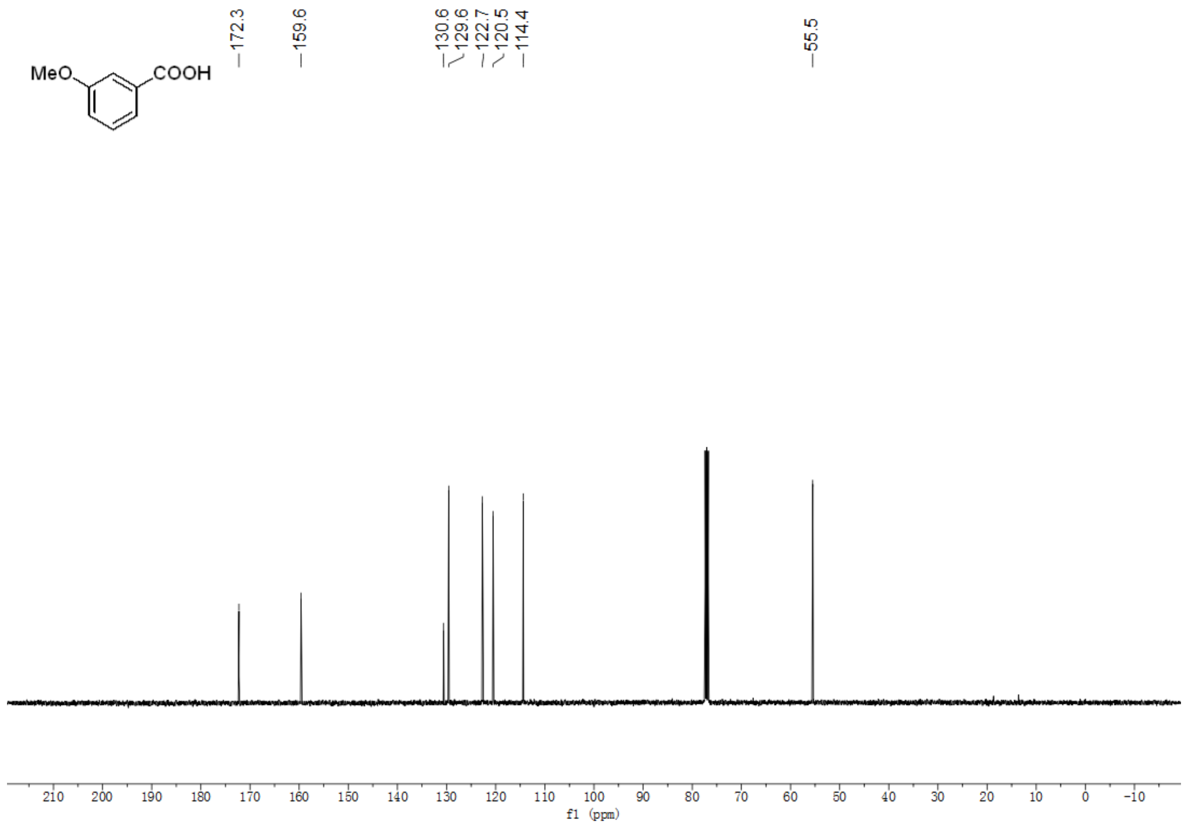

Supplementary Figure 40. ^{13}C NMR spectra of compound **4j**

3-(benzyloxy)benzoic acid (4k, *$CDCl_3$* as solvent)

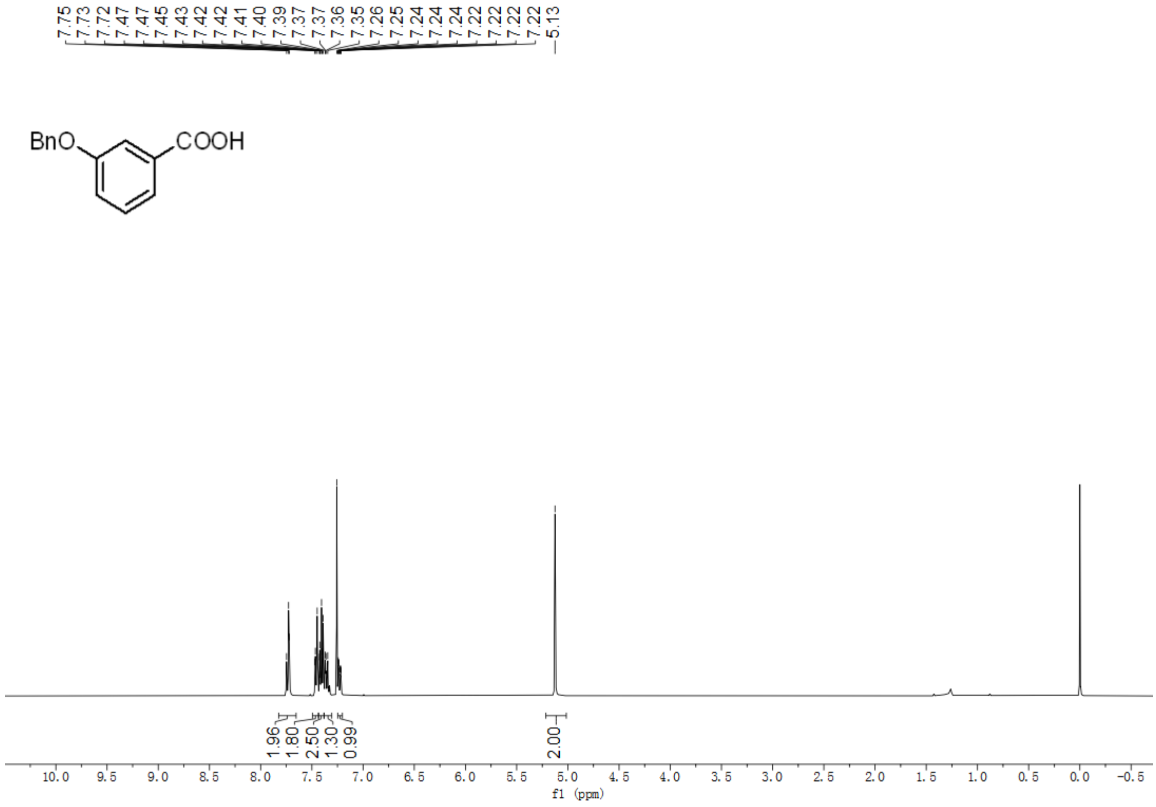

Supplementary Figure 41. ^{1}H NMR spectra of compound **4k**

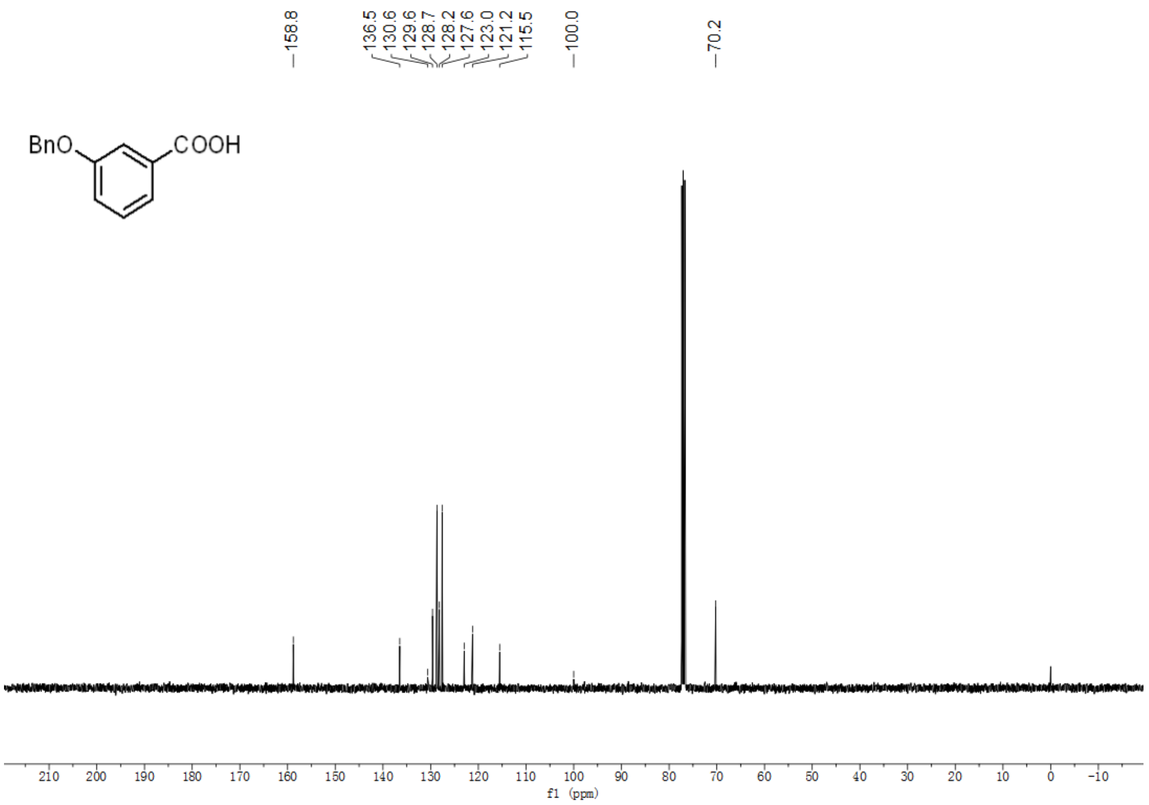

Supplementary Figure 42. ^{13}C NMR spectra of compound **4k**

3-vinylbenzoic acid (4l, *DMSO-d_6* as solvent)

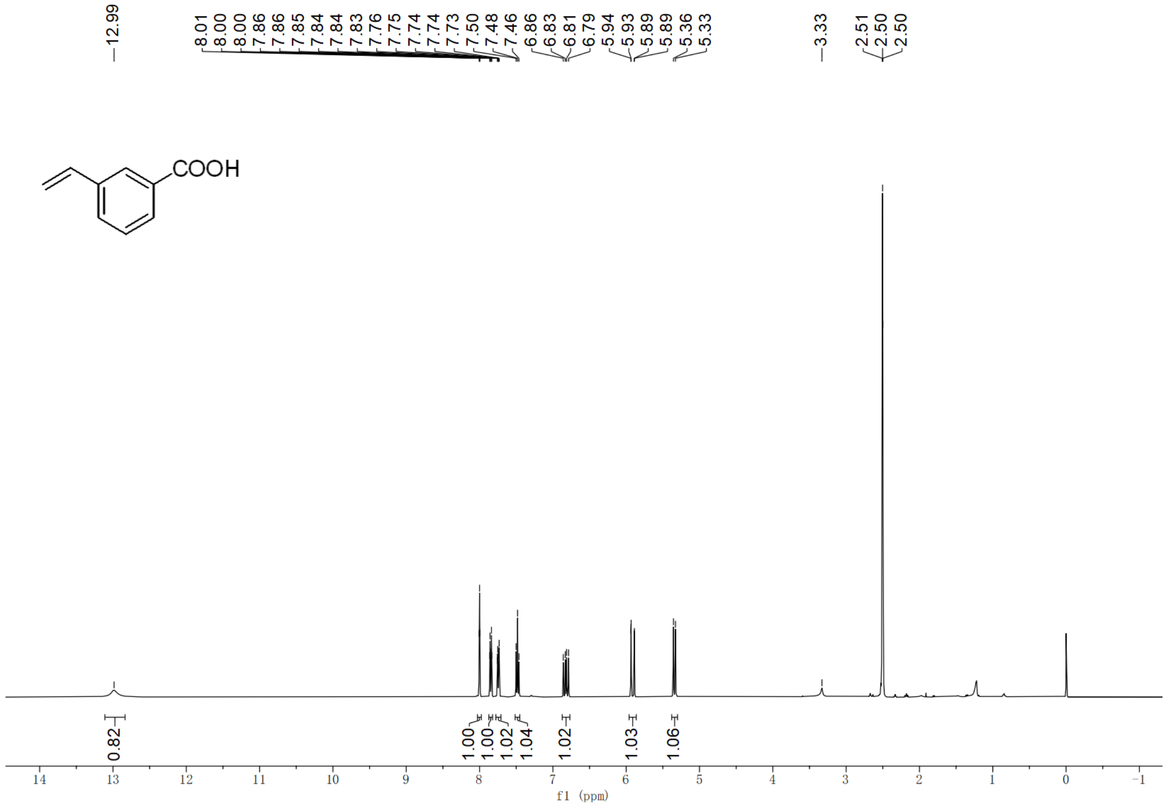

Supplementary Figure 43. ^{1}H NMR spectra of compound **4l**

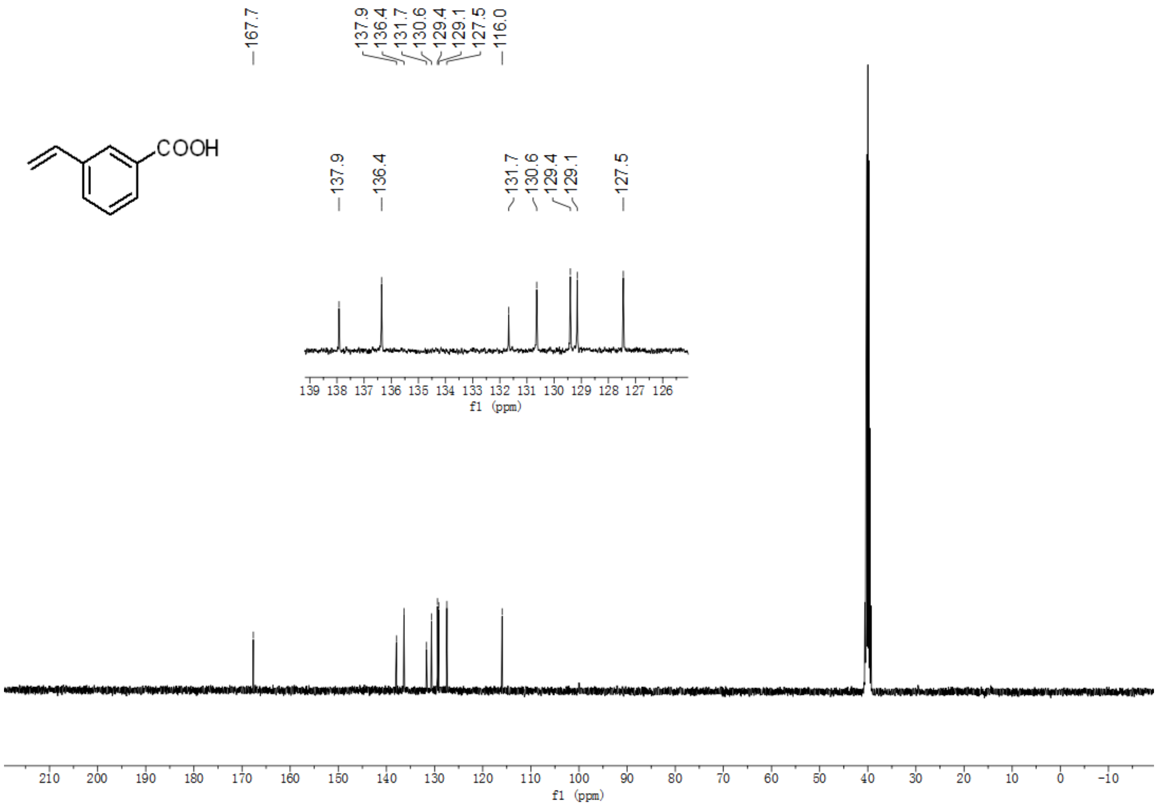

Supplementary Figure 44. ^{13}C NMR spectra of compound **4l**

3-formylbenzoic acid (4m, *DMSO-d_6* as solvent)

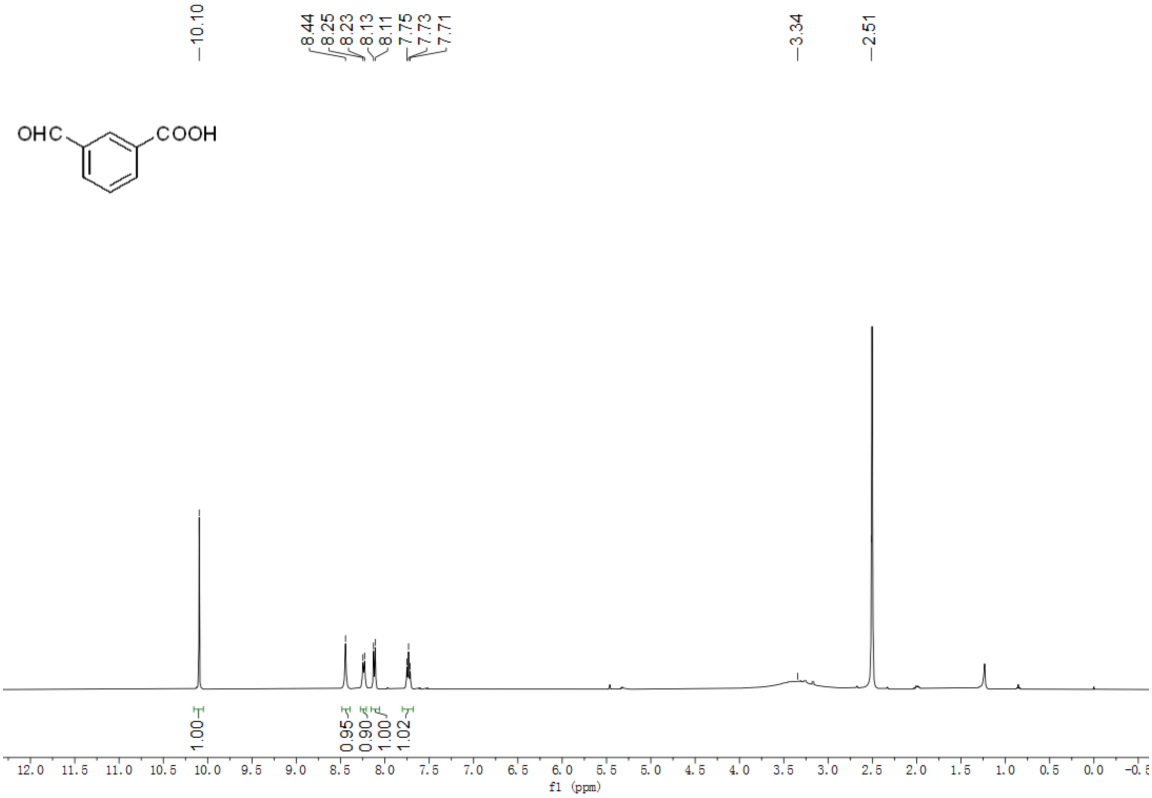

Supplementary Figure 45. ^{1}H NMR spectra of compound **4m**

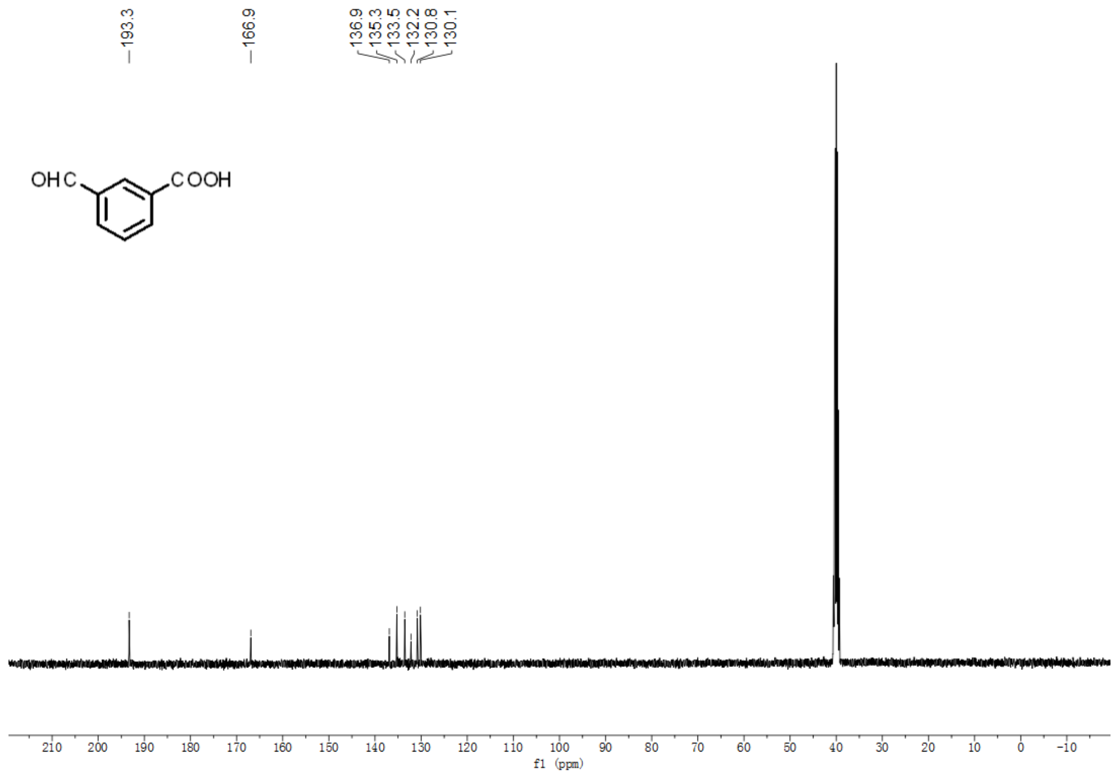

Supplementary Figure 46. ^{13}C NMR spectra of compound **4m**

[1,1'-biphenyl]-2-carboxylic acid (4n, *DMSO-d₆* as solvent)

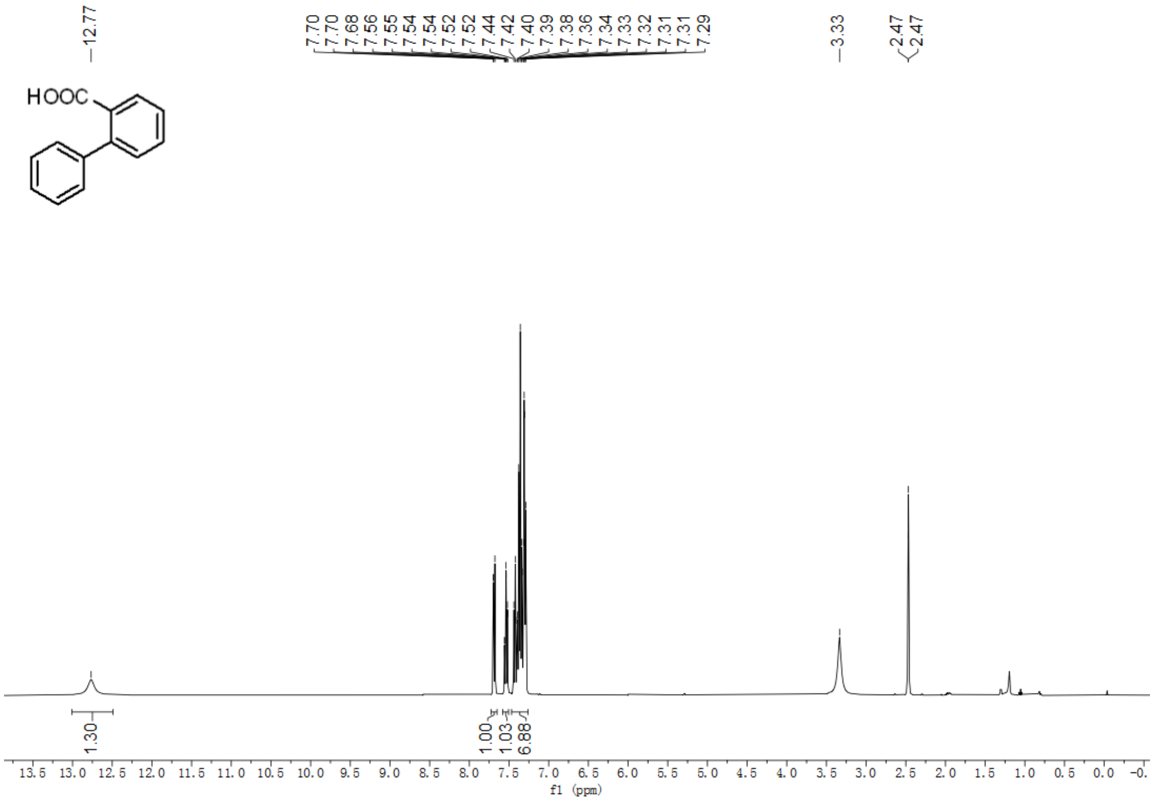

Supplementary Figure 47. ^{1}H NMR spectra of compound **4n**

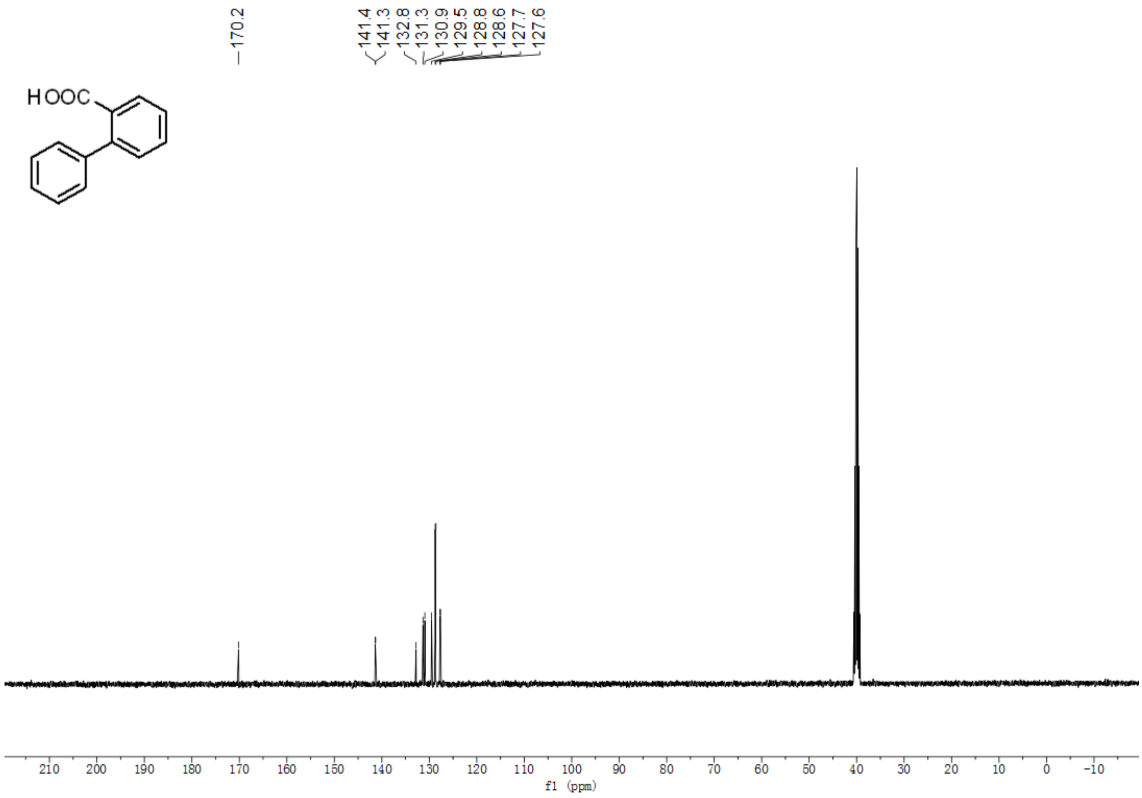

Supplementary Figure 48. ^{13}C NMR spectra of compound **4n**

[1,1':3',1''-terphenyl]-5'-carboxylic acid (4o, *DMSO-d₆* as solvent)

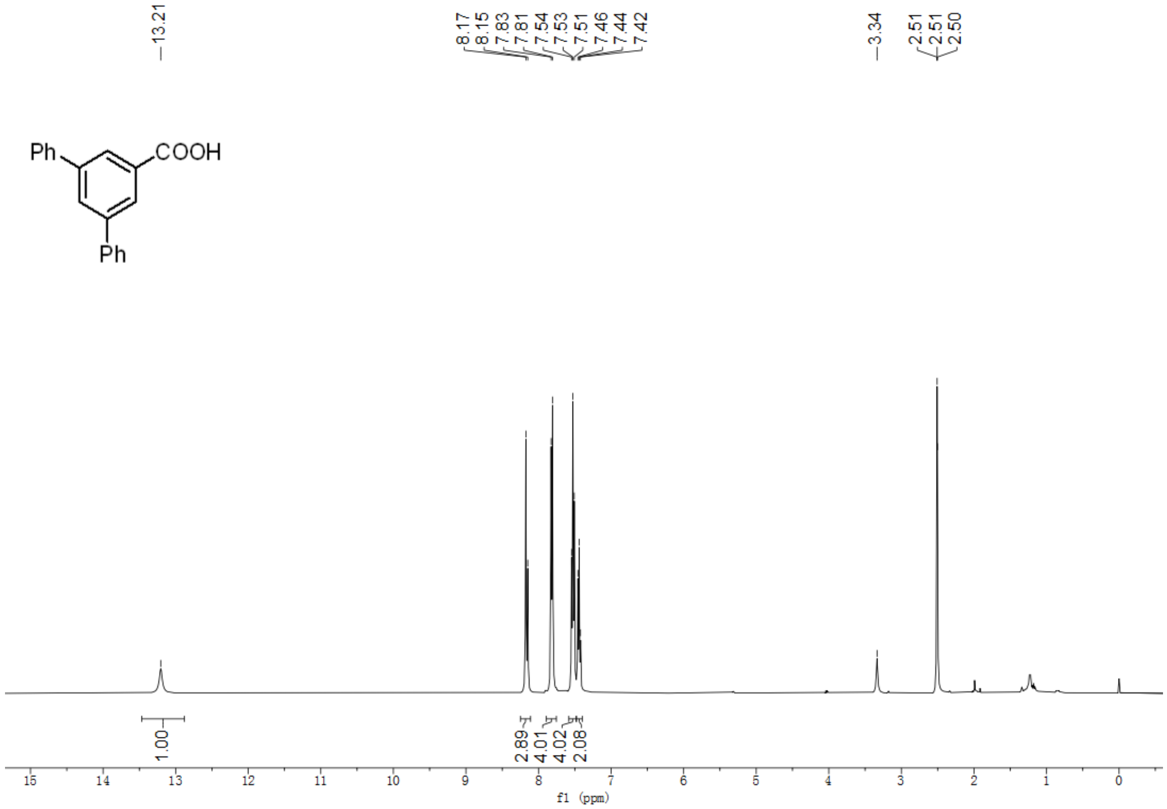

Supplementary Figure 49. ^{1}H NMR spectra of compound **4o**

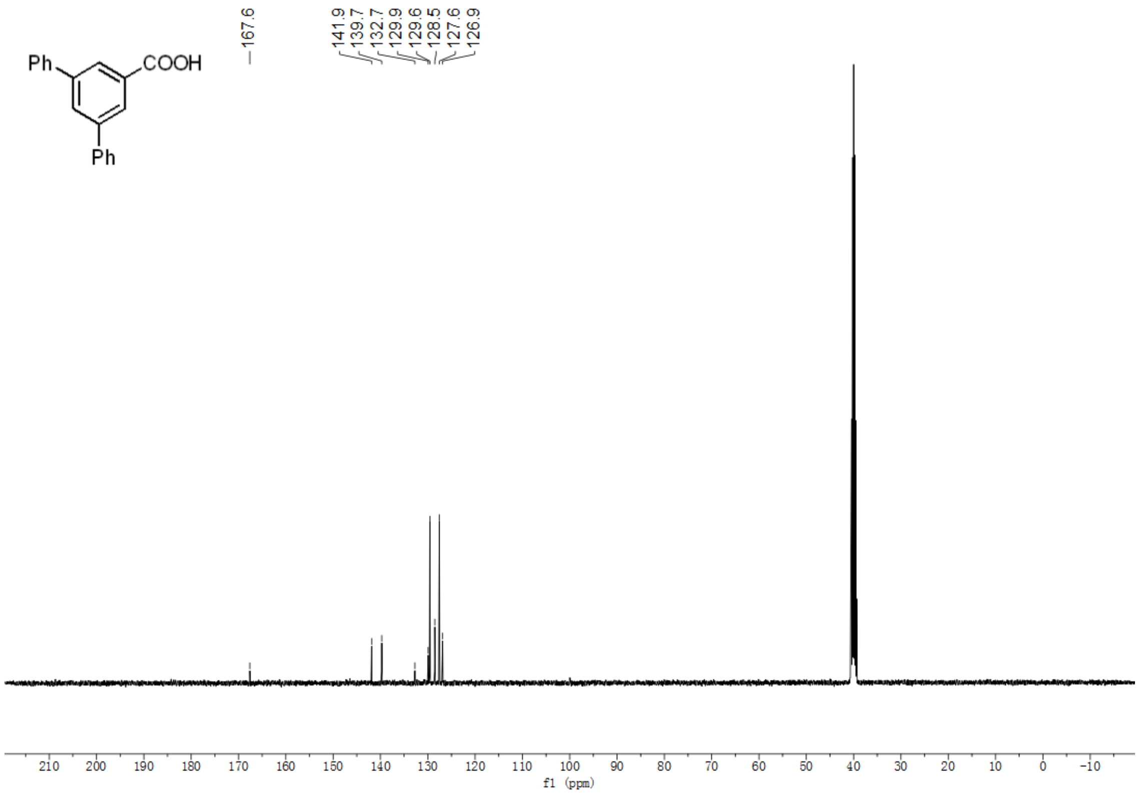

Supplementary Figure 50. ^{13}C NMR spectra of compound **4o**

benzo[*d*][1,3]dioxole-5-carboxylic acid (4p, *DMSO-d_6* as solvent)

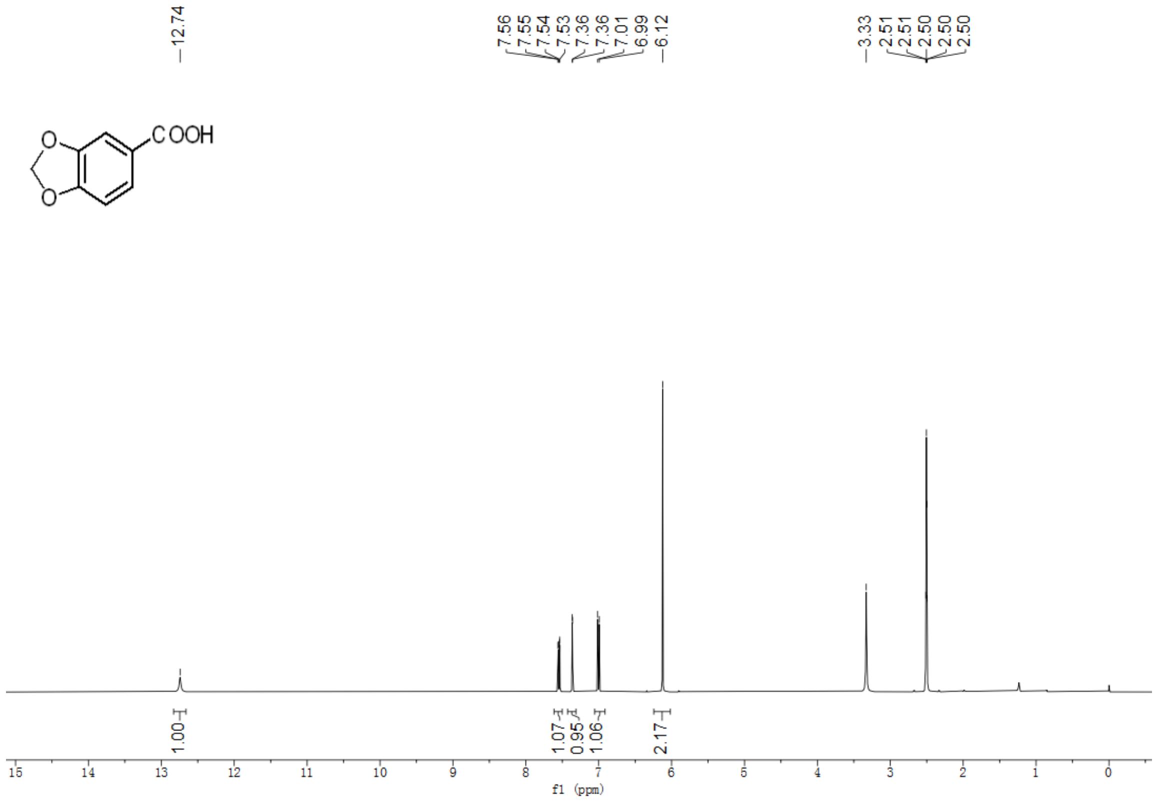

Supplementary Figure 51. ^{1}H NMR spectra of compound **4p**

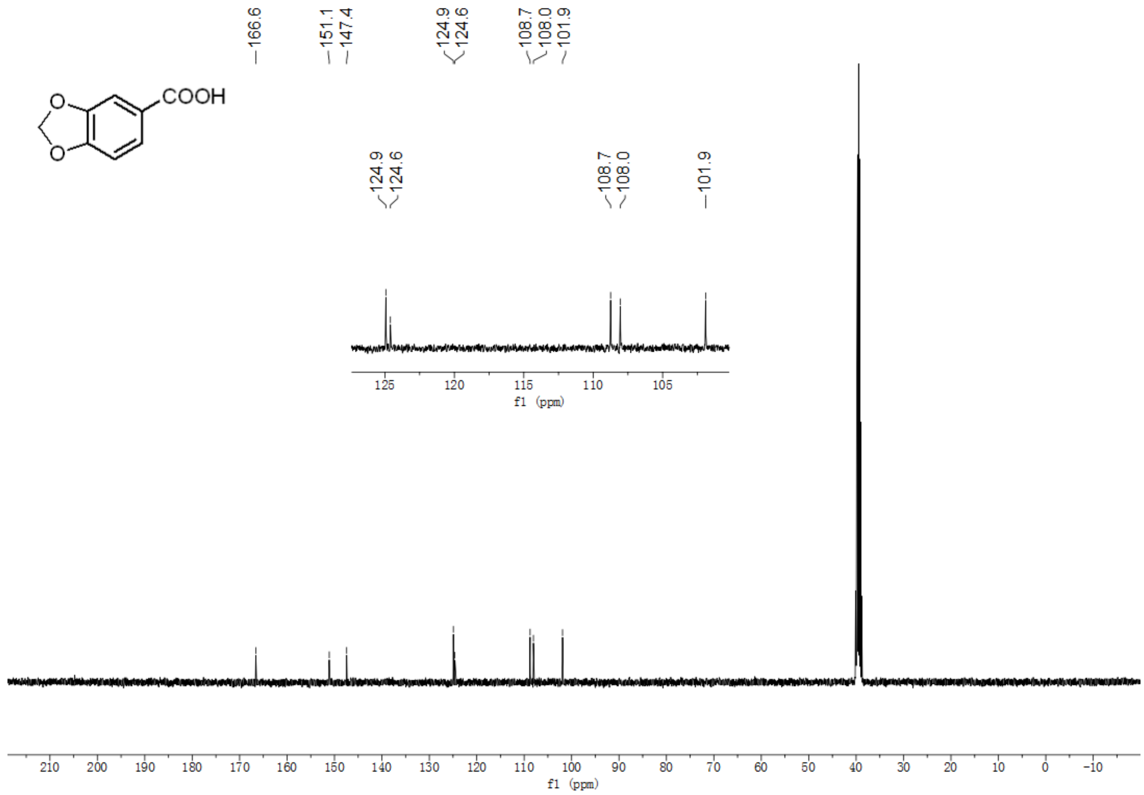

Supplementary Figure 52. ^{13}C NMR spectra of compound **4p**

6-methoxy-2-naphthoic acid (4q, *DMSO-d$_6$* as solvent)

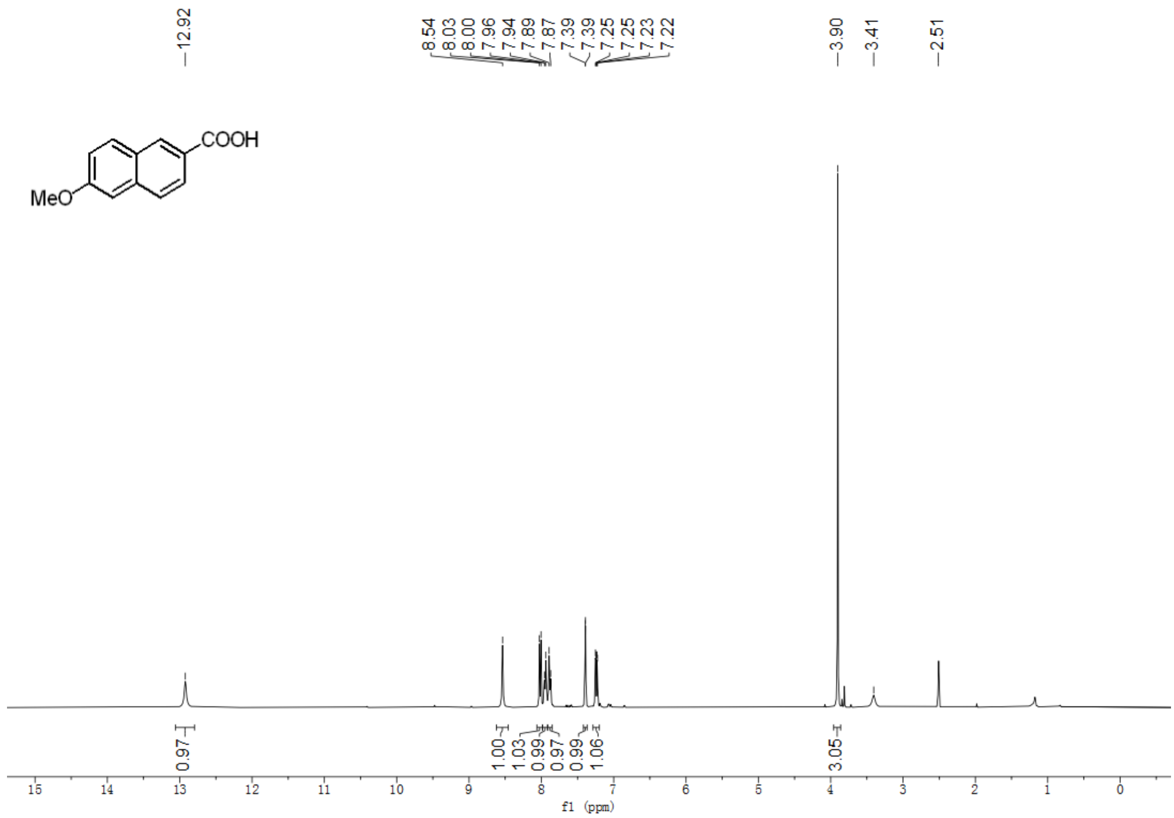

Supplementary Figure 53. ^{1}H NMR spectra of compound **4q**

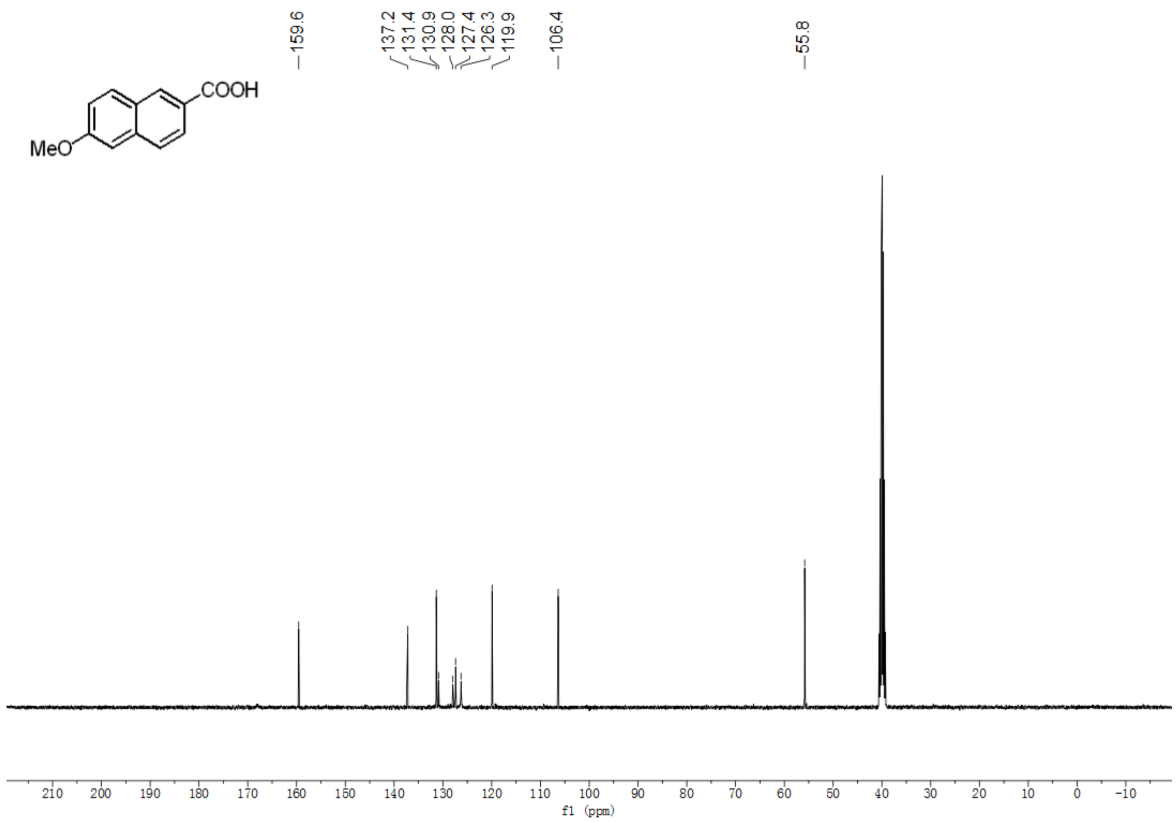

Supplementary Figure 54. ^{13}C NMR spectra of compound **4q**

dibenzo[*b,d*]thiophene-2-carboxylic acid (4r, *DMSO-d_6* as solvent)

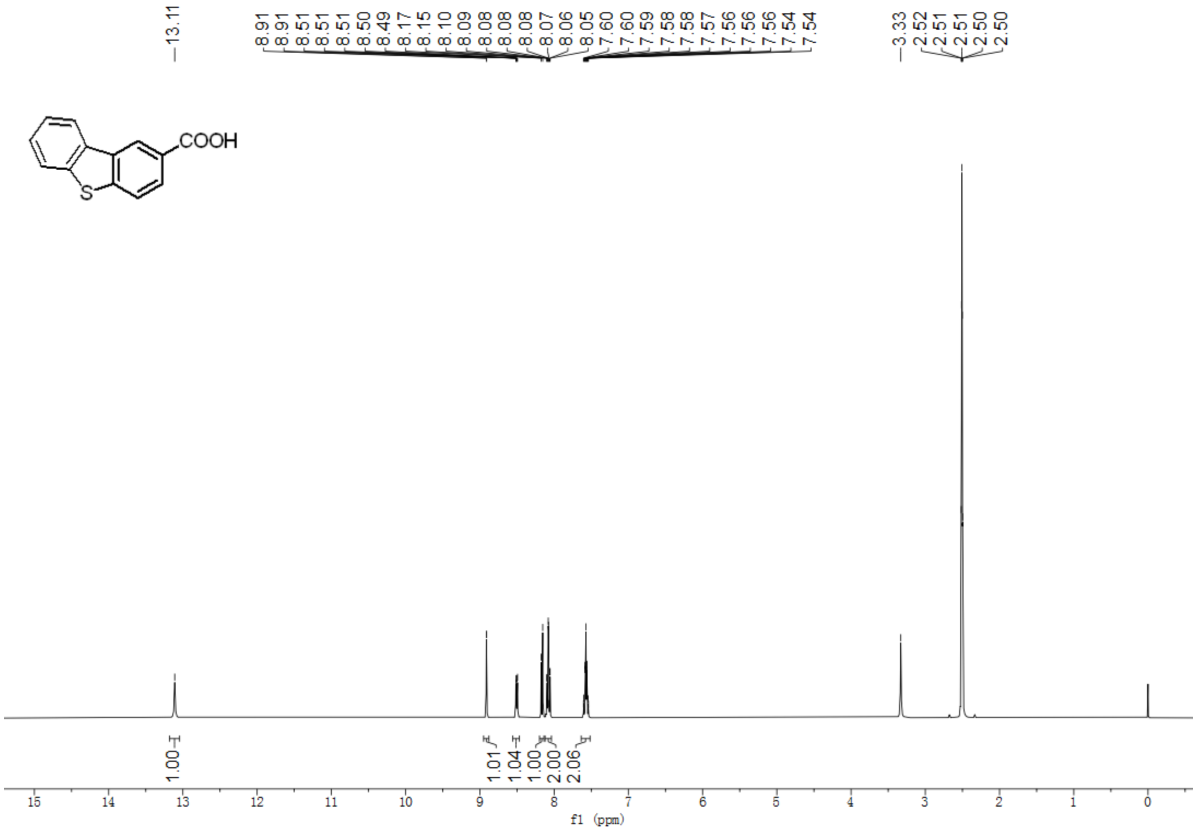

Supplementary Figure 55. ^{1}H NMR spectra of compound **4r**

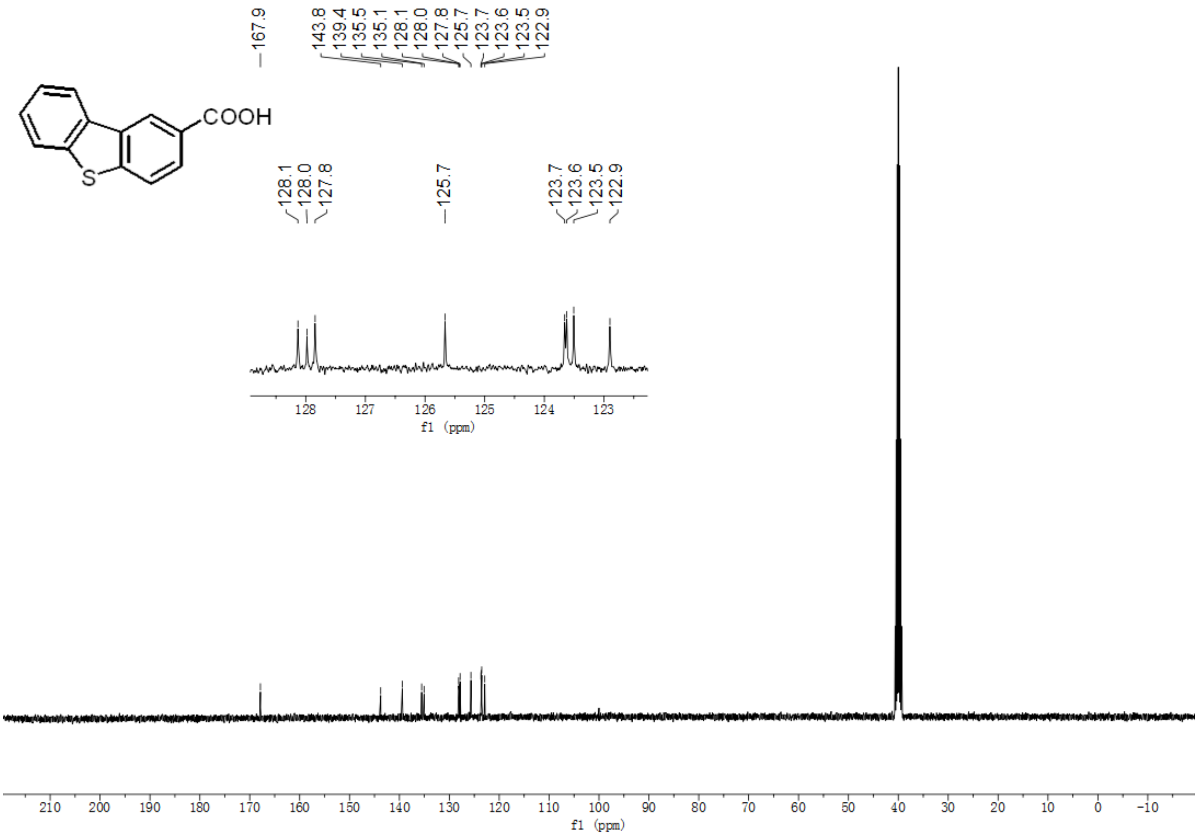

Supplementary Figure 56. ^{13}C NMR spectra of compound **4r**

dibenzo[*b,d*]furan-2-carboxylic acid (4s, *DMSO-d$_6$* as solvent)

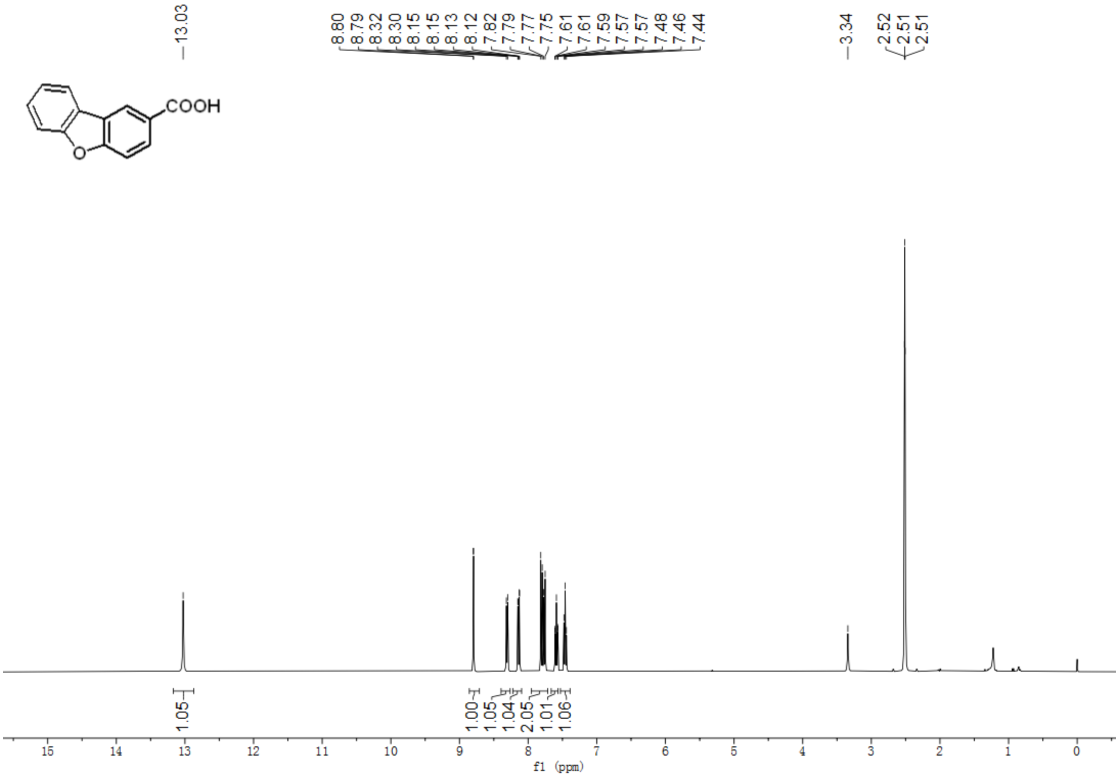

Supplementary Figure 57. ^{1}H NMR spectra of compound **4s**

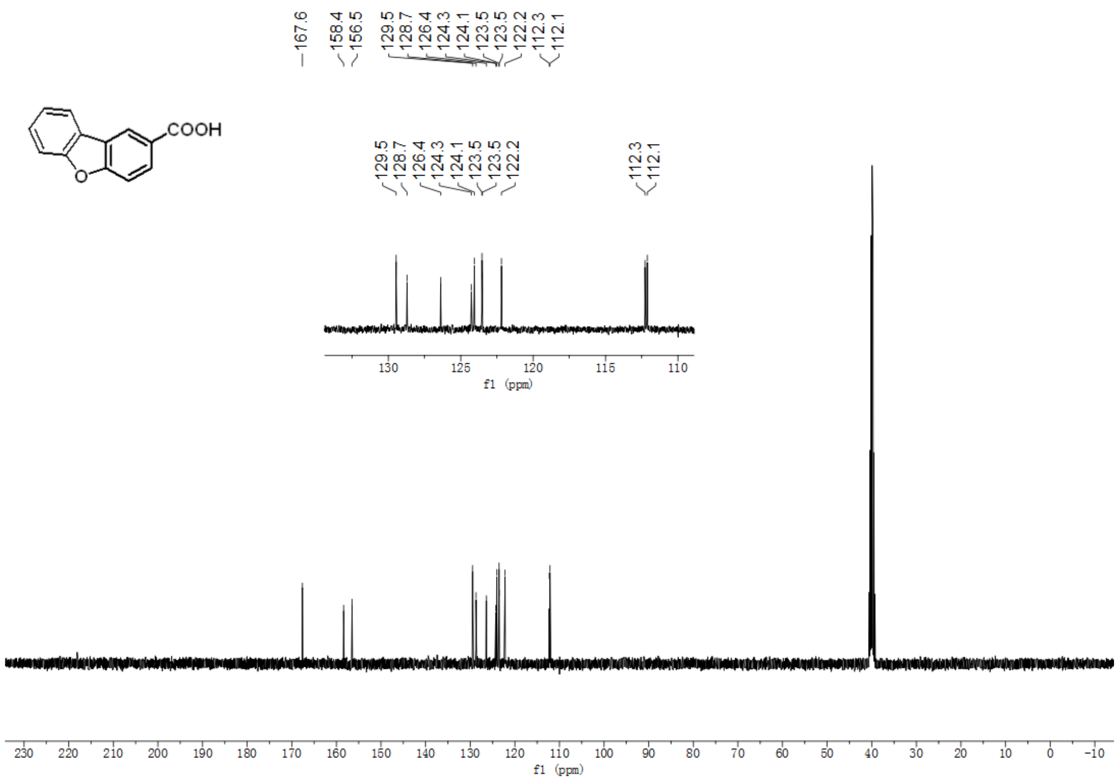

Supplementary Figure 58. ^{13}C NMR spectra of compound **4s**

9-(*tert*-butoxycarbonyl)-9*H*-carbazole-3-carboxylic acid (4t, *DMSO-d_6* as solvent)

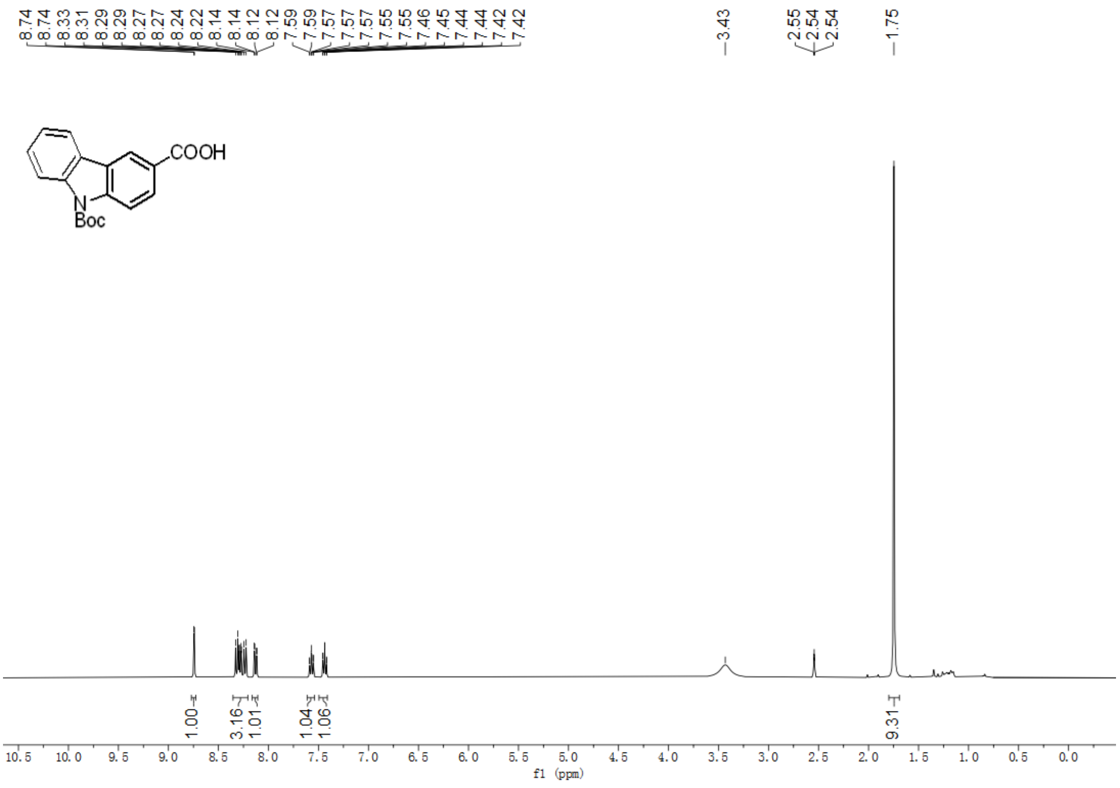

Supplementary Figure 59. ^{1}H NMR spectra of compound **4t**

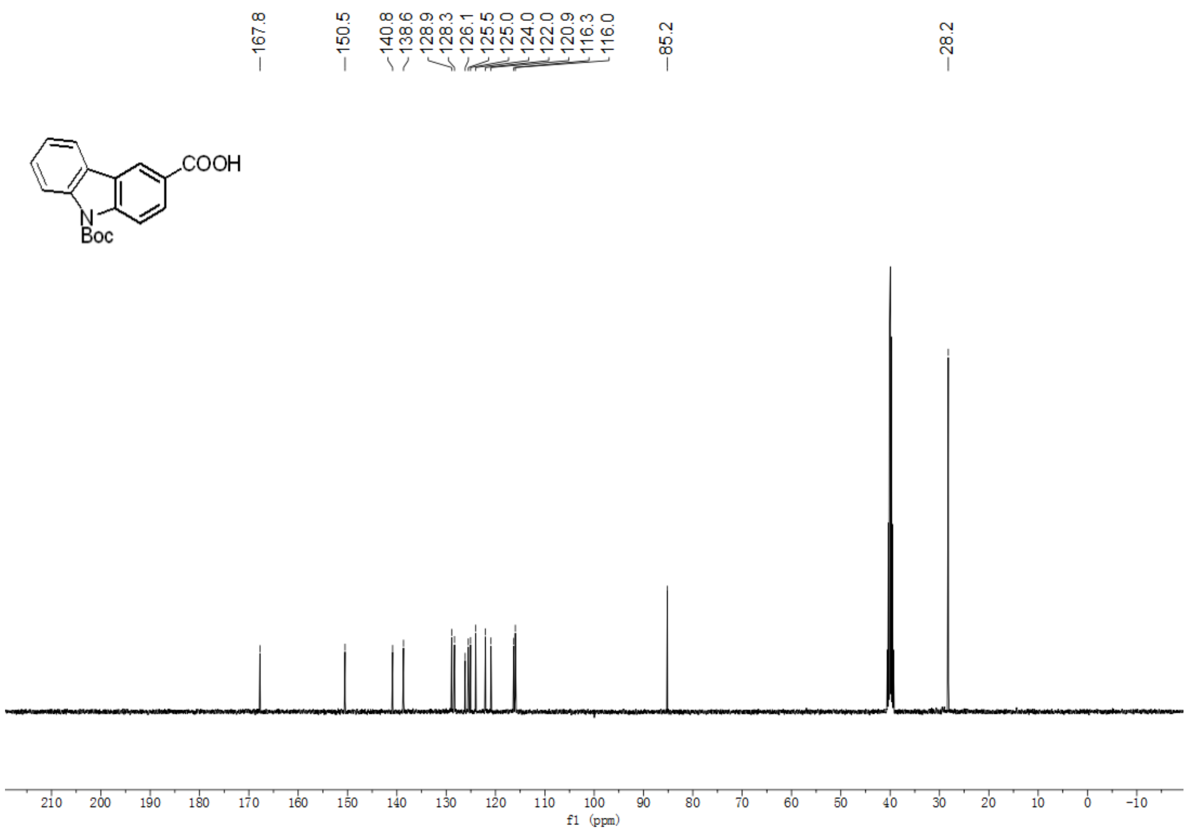

Supplementary Figure 60. ^{13}C NMR spectra of compound **4t**

(8*R*,9*S*,13*S*,14*S*)-13-methyl-17-oxo-7,8,9,11,12,13,14,15,16,17-decahydro-6*H* cyclopenta[*a*]phenanthrene-3-carboxylic acid (4ab, *DMSO-d₆* as solvent)

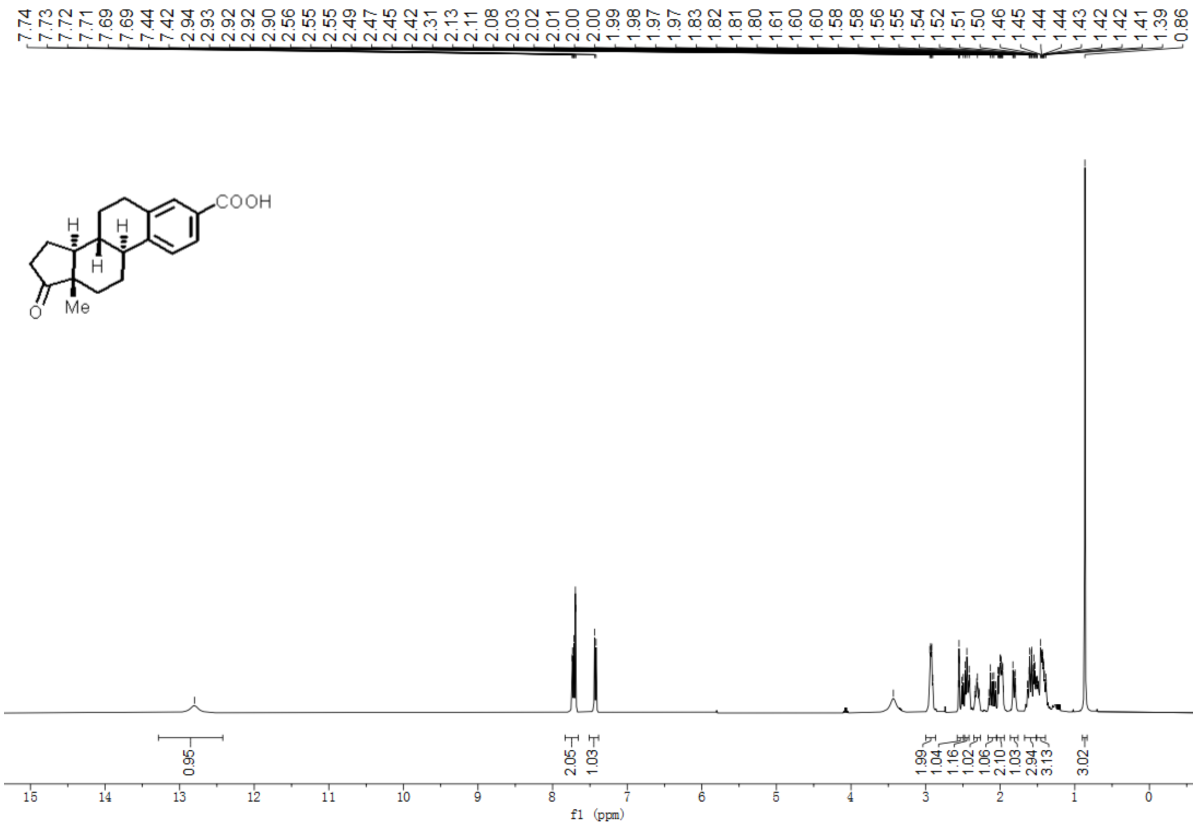

Supplementary Figure 61. ^{1}H NMR spectra of compound **4ab**

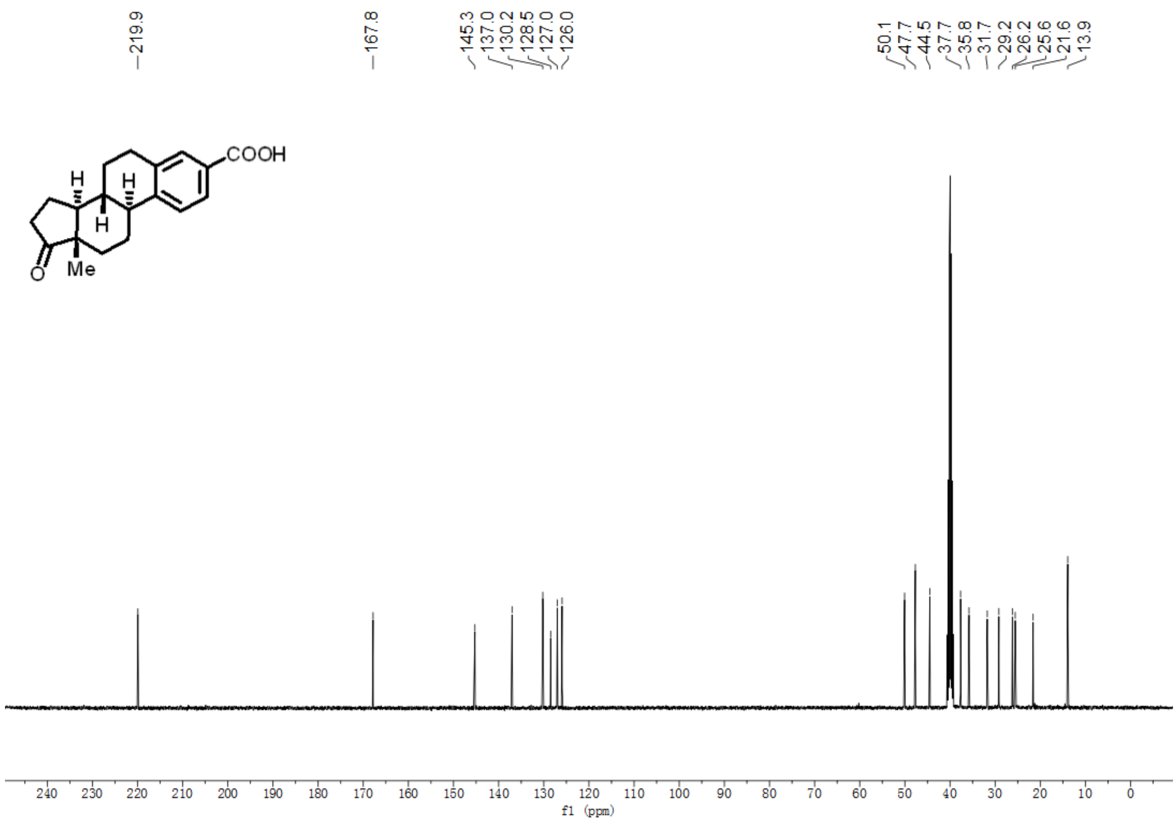

Supplementary Figure 62. ^{13}C NMR spectra of compound **4ab**

4-(*tert*-butyl) benzoic acid (4ac, *CDCl$_3$* as solvent)

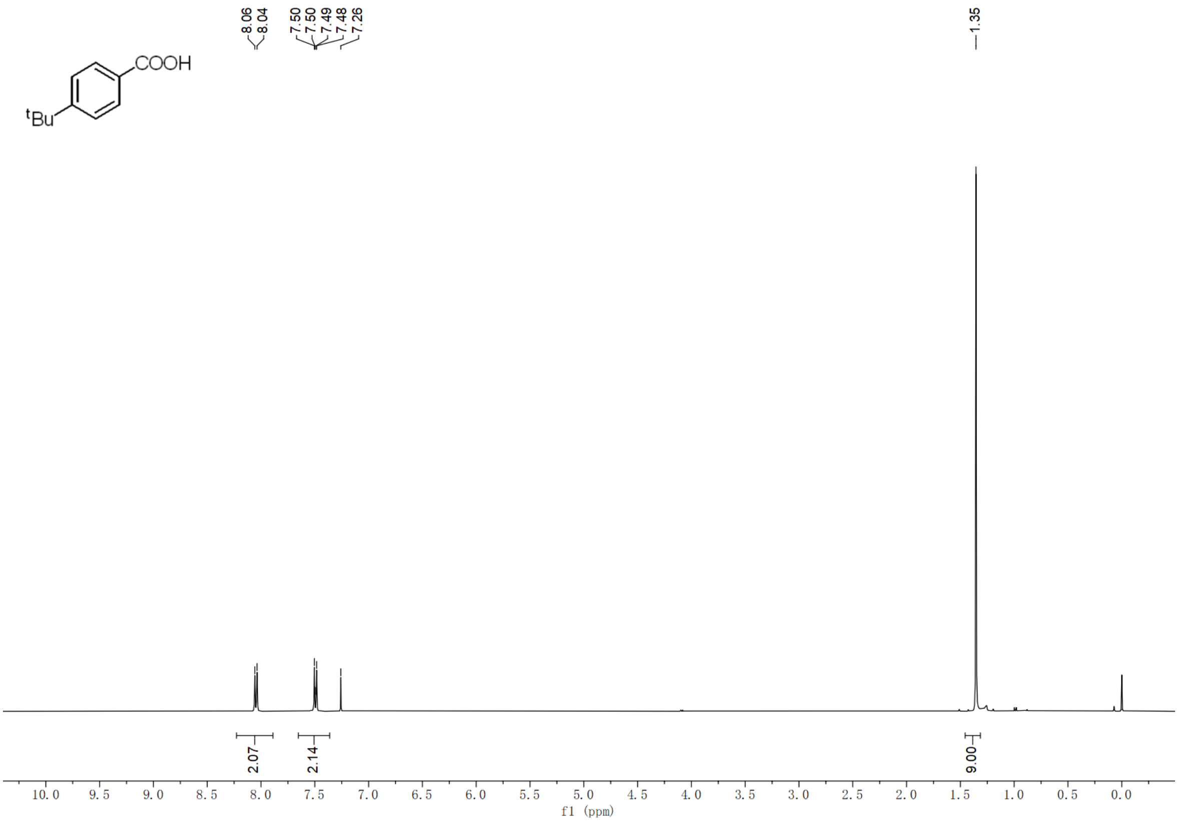

Supplementary Figure 63. ^{1}H NMR spectra of compound **4ac**

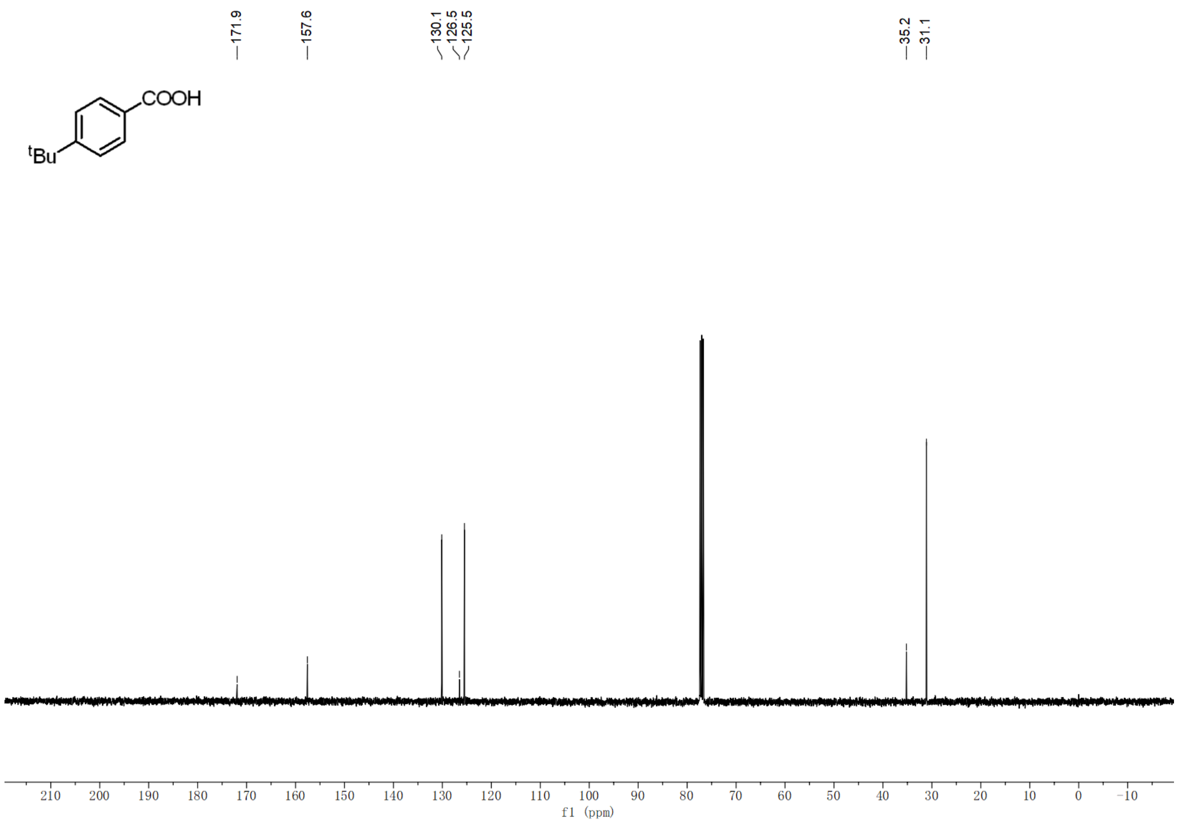

Supplementary Figure 64. ^{13}C NMR spectra of compound **4ac**

4-(trifluoromethoxy)benzoic acid (4ad, *$CDCl_3$* as solvent)

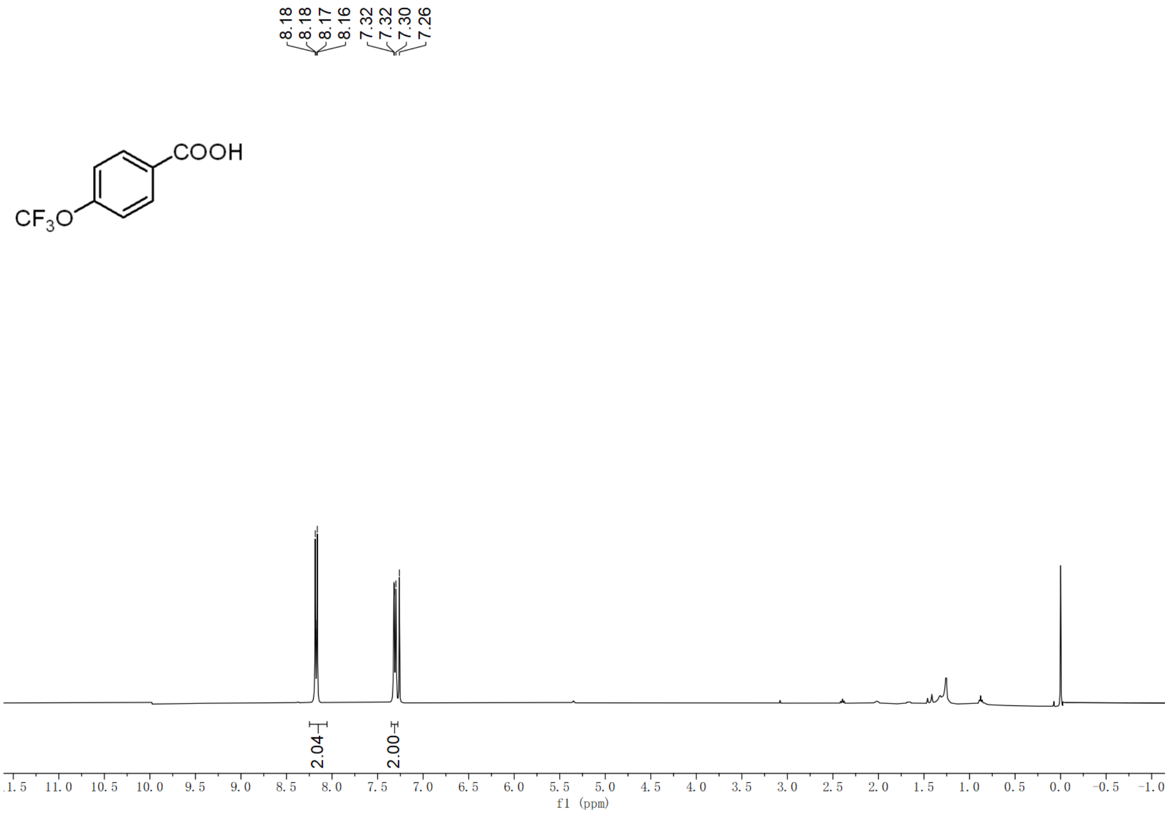

Supplementary Figure 65. ^{1}H NMR spectra of compound **4ad**

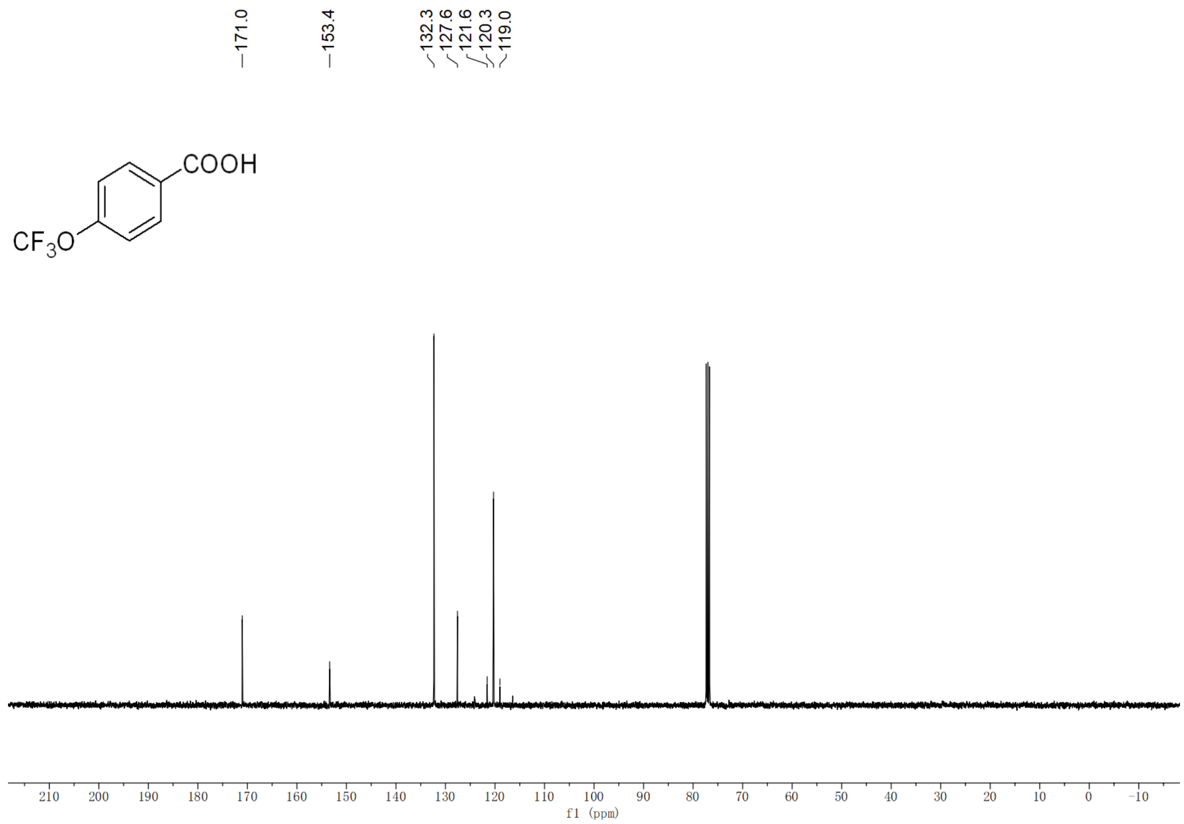

Supplementary Figure 66. ^{13}C NMR spectra of compound **4ad**

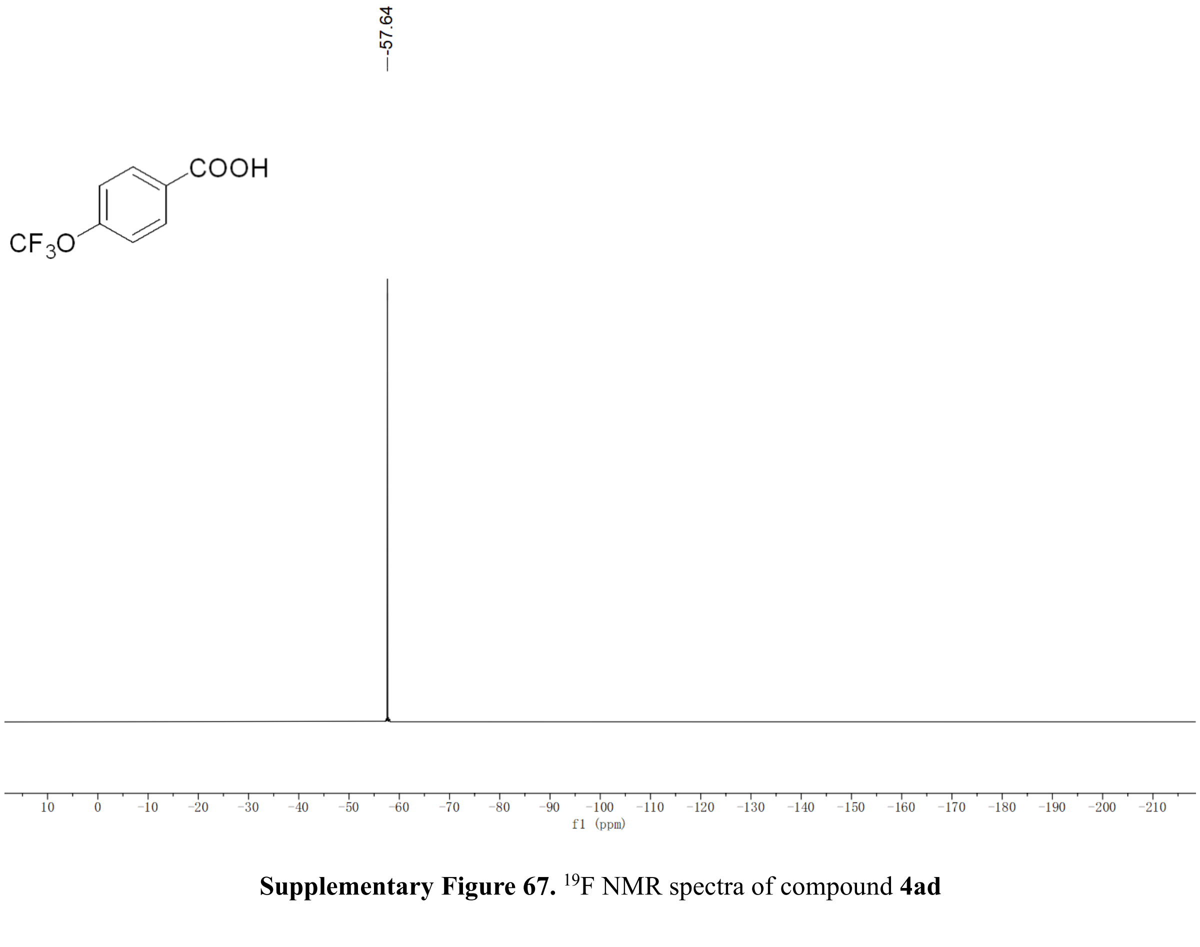

Supplementary Figure 67. ^{19}F NMR spectra of compound **4ad**

3,5-di-*tert*-butylbenzoic acid (4ae, $CDCl_3$ as solvent)

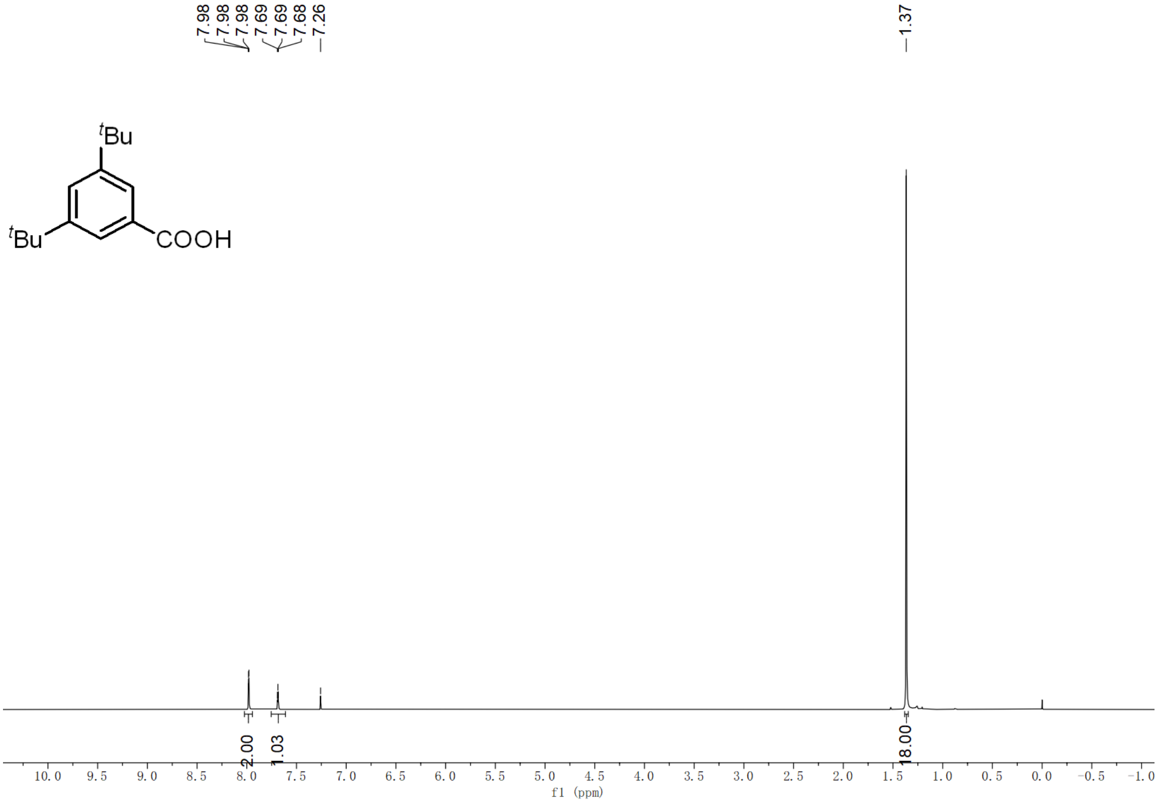

Supplementary Figure 68. ^{1}H NMR spectra of compound **4ae**

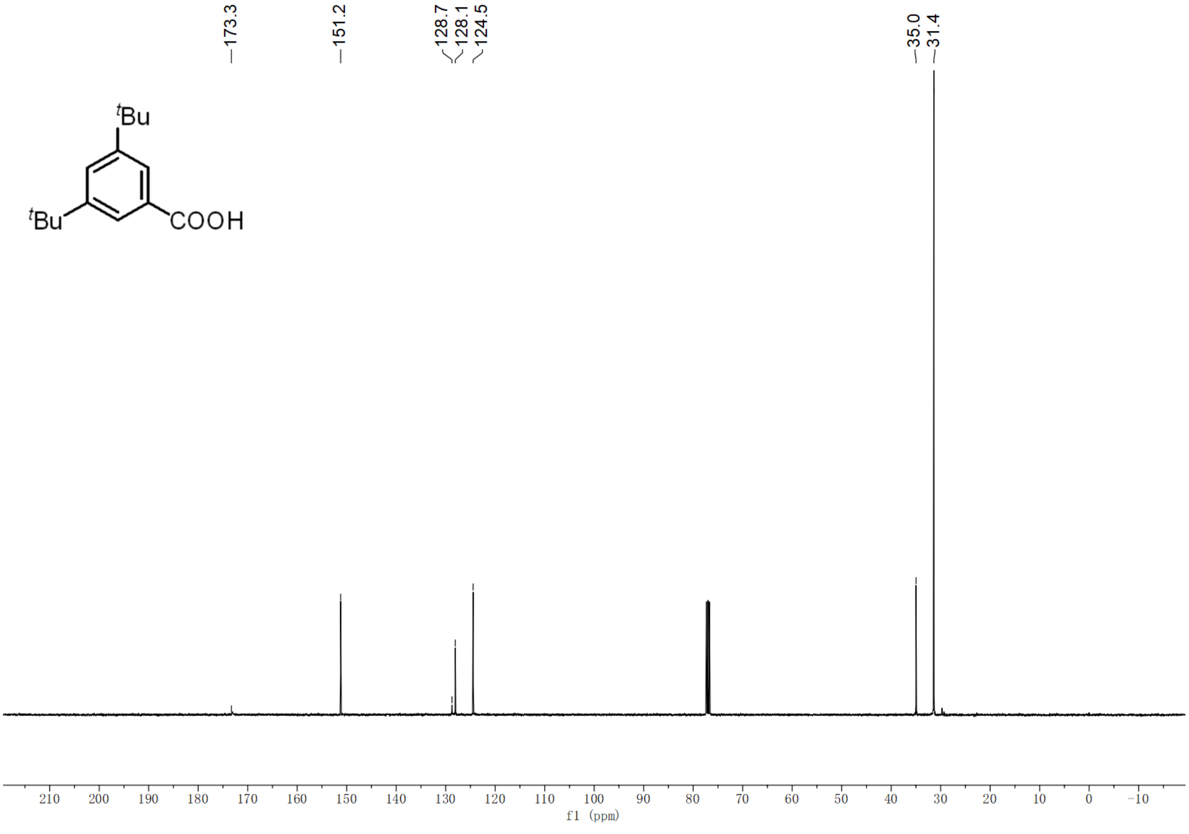

Supplementary Figure 69. ^{13}C NMR spectra of compound **4ae**

4-(trifluoromethyl)benzoic acid (2m, *DMSO-d_6* as solvent)

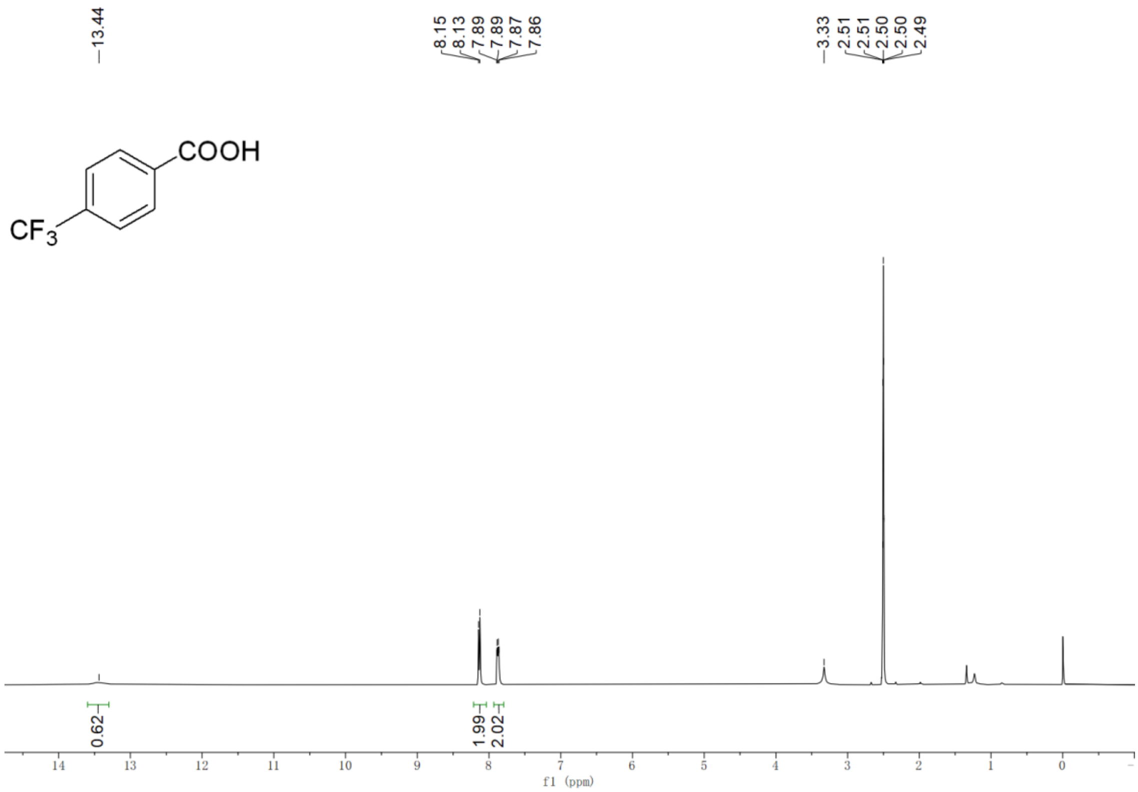

Supplementary Figure 70. ^{1}H NMR spectra of compound **2m**

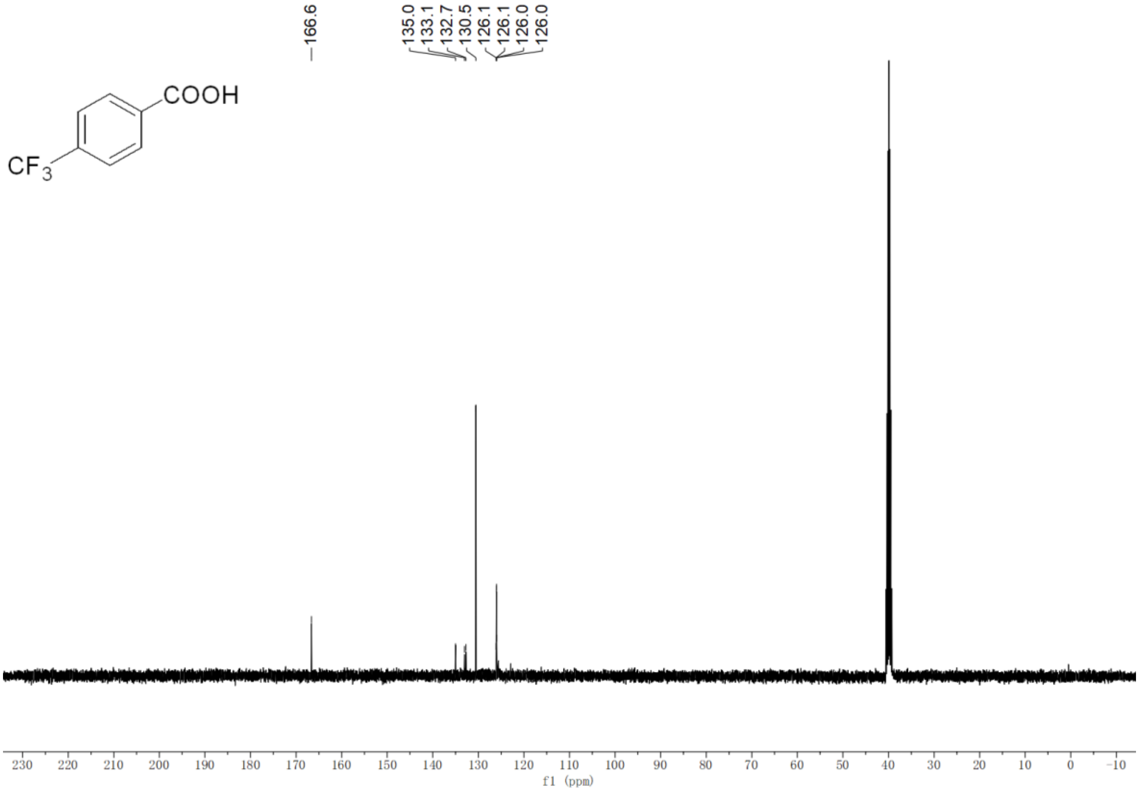

Supplementary Figure 71. ^{13}C NMR spectra of compound **2m**

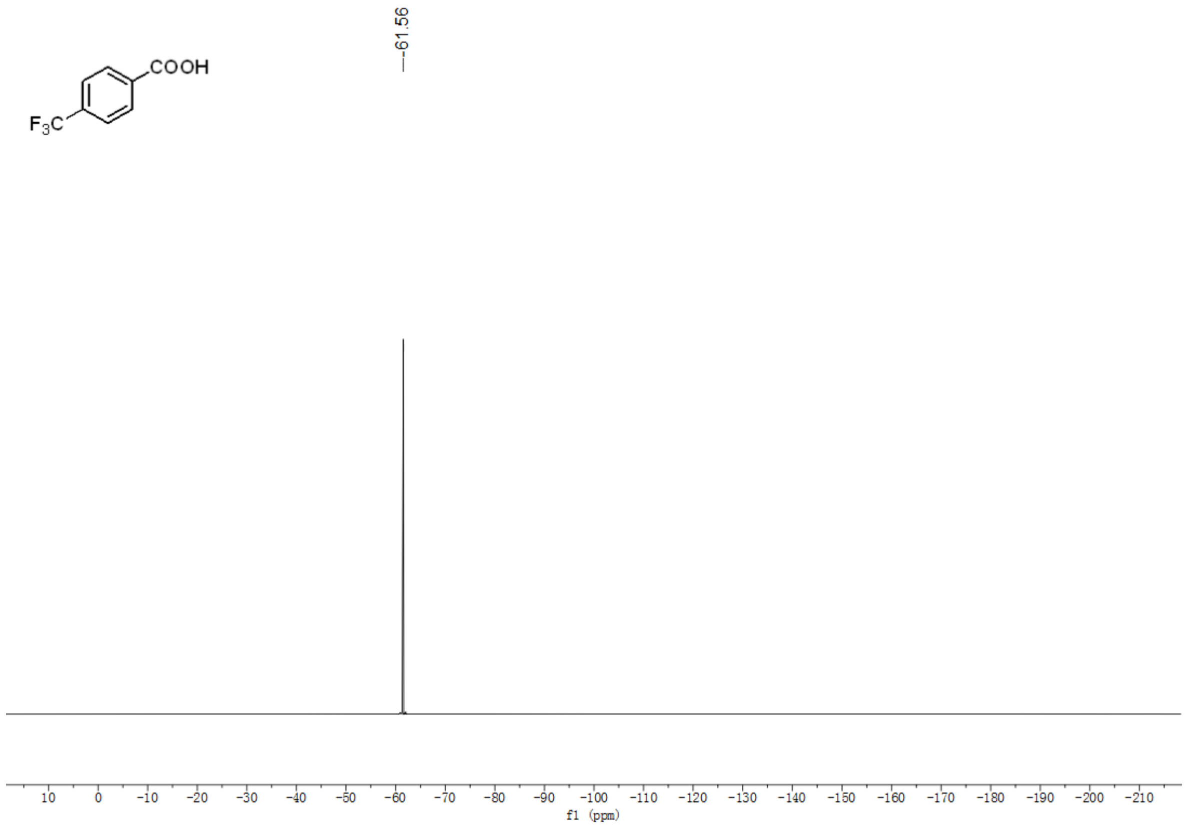

Supplementary Figure 72. ^{19}F NMR spectra of compound **2m**

7-acetoxyheptanoic acid (6a, *$CDCl_3$* as solvent)

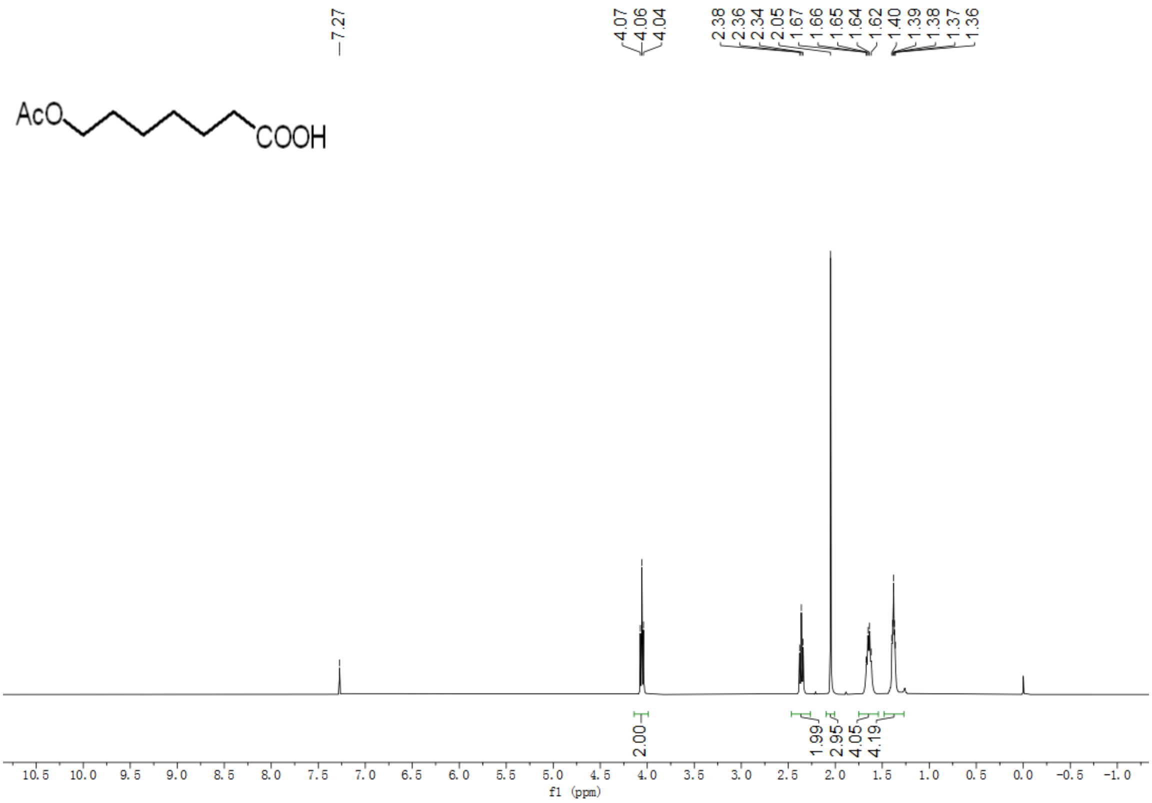

Supplementary Figure 73. ^{1}H NMR spectra of compound **6a**

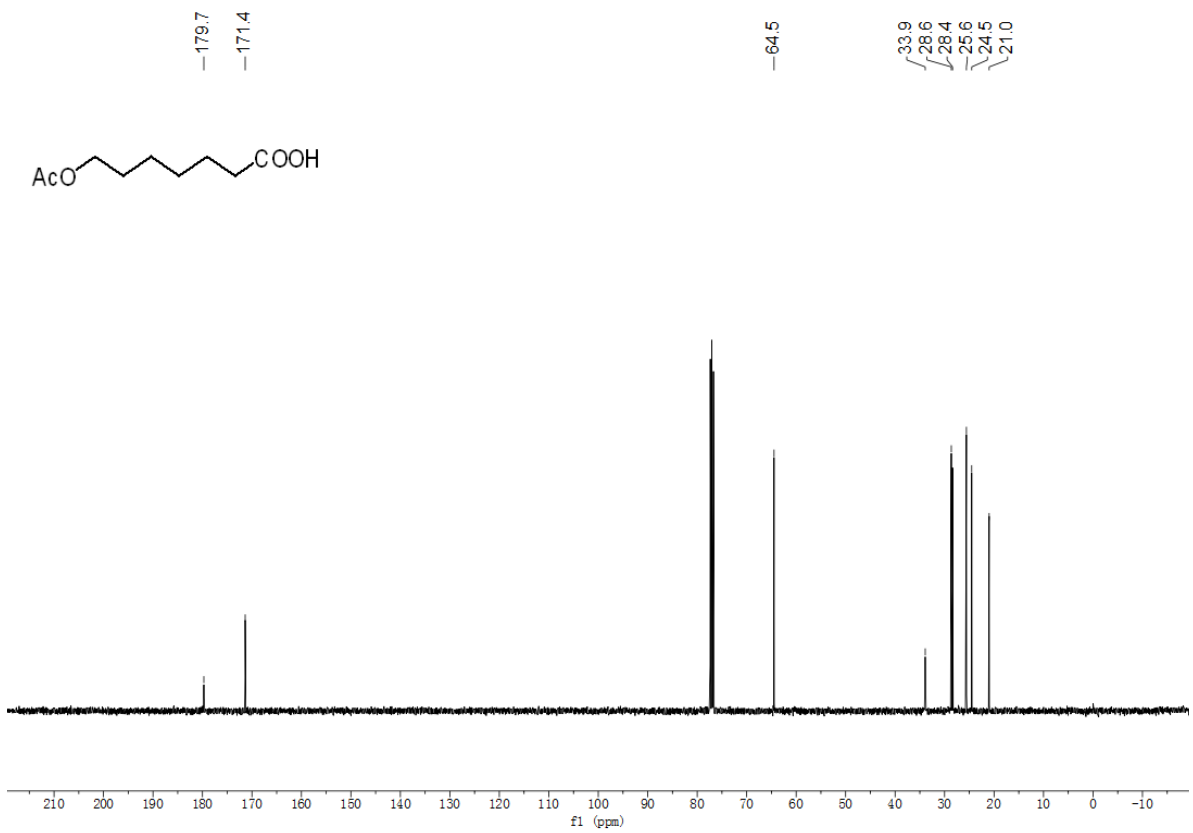

Supplementary Figure 74. ^{13}C NMR spectra of compound **6a**

7-(pivaloyloxy)heptanoic acid (6b, *CDCl₃* as solvent)

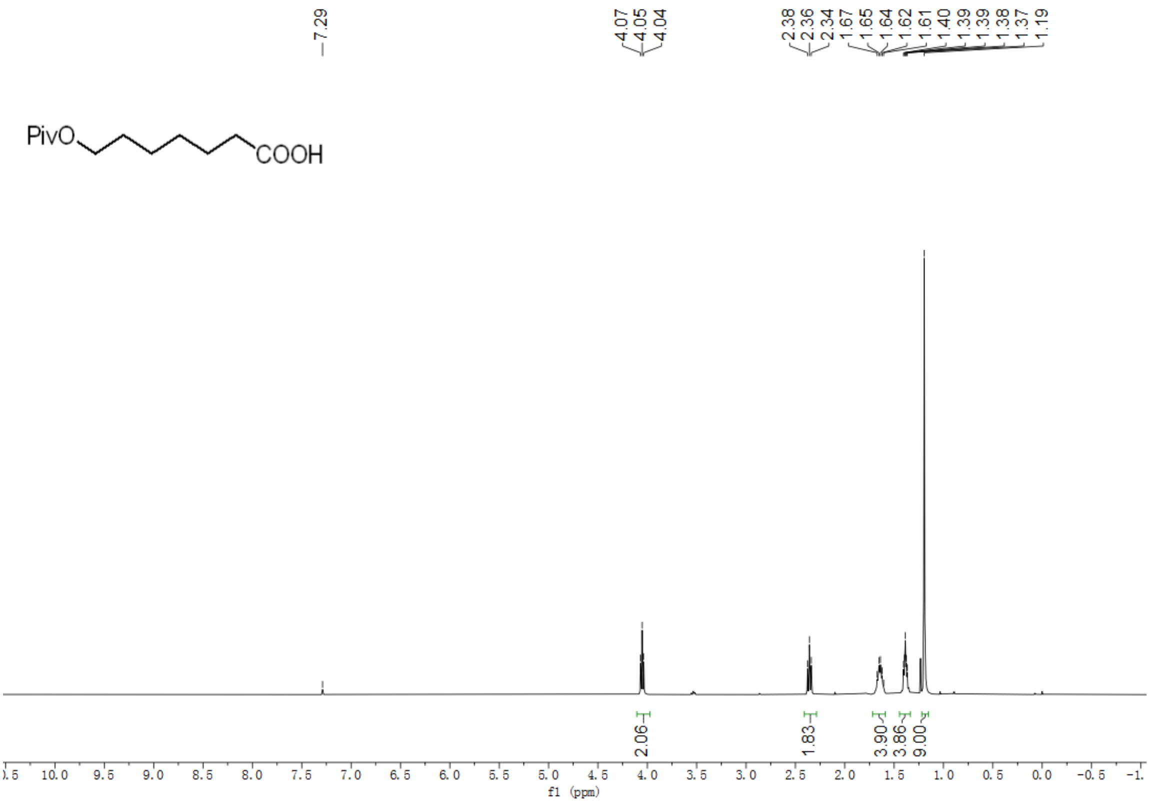

Supplementary Figure 75. ^{1}H NMR spectra of compound **6b**

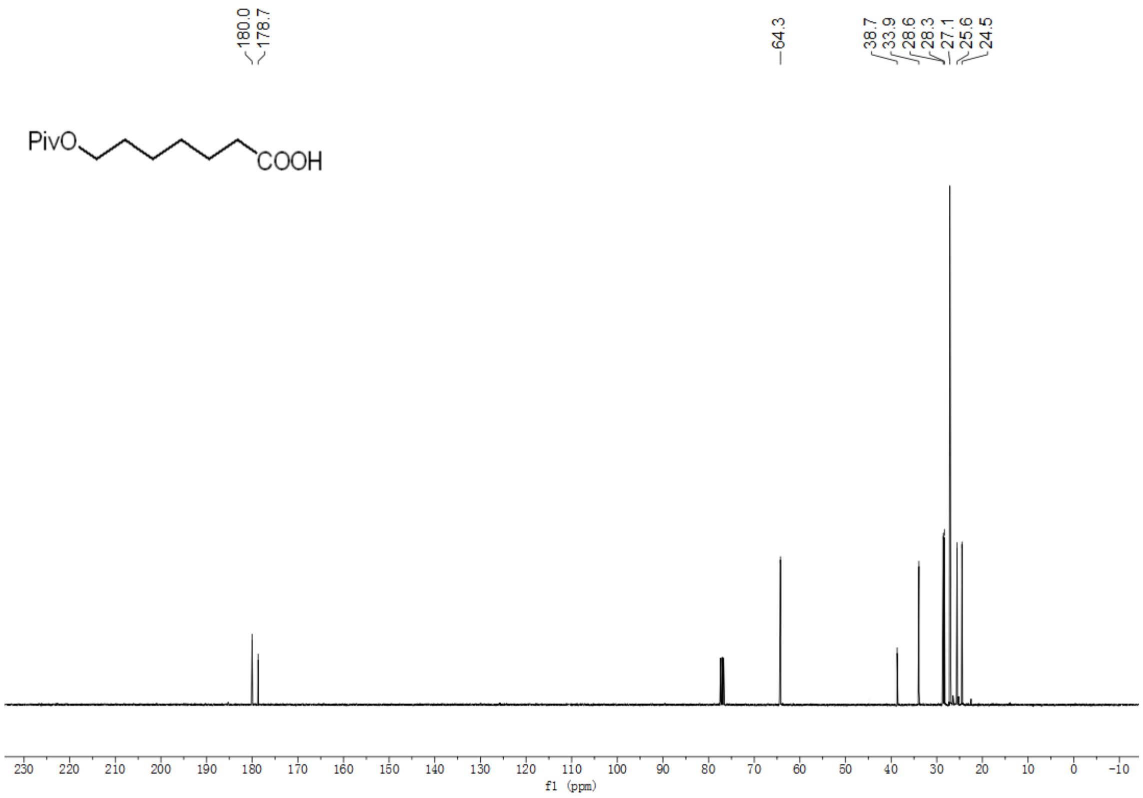

Supplementary Figure 76. ^{13}C NMR spectra of compound **6b**

7-(benzyloxy)heptanoic acid (6c, *CDCl₃* as solvent)

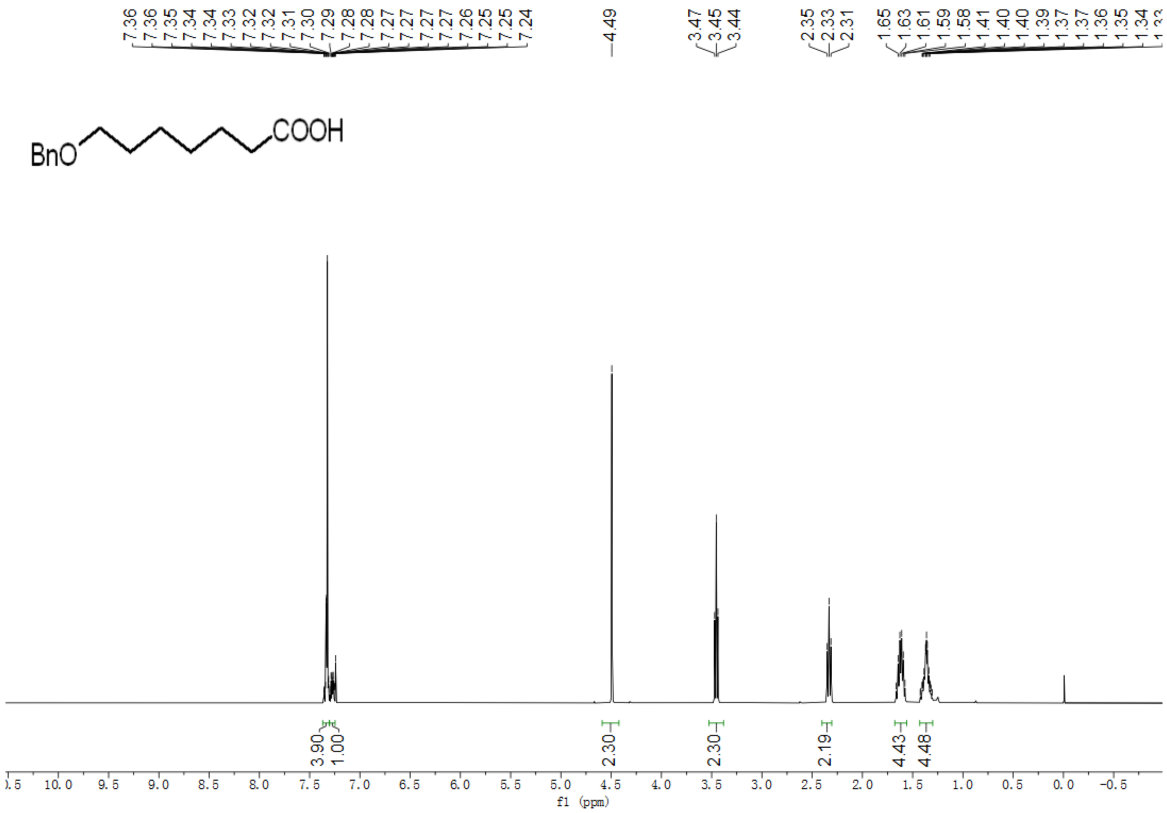

Supplementary Figure 77. ^{1}H NMR spectra of compound **6c**

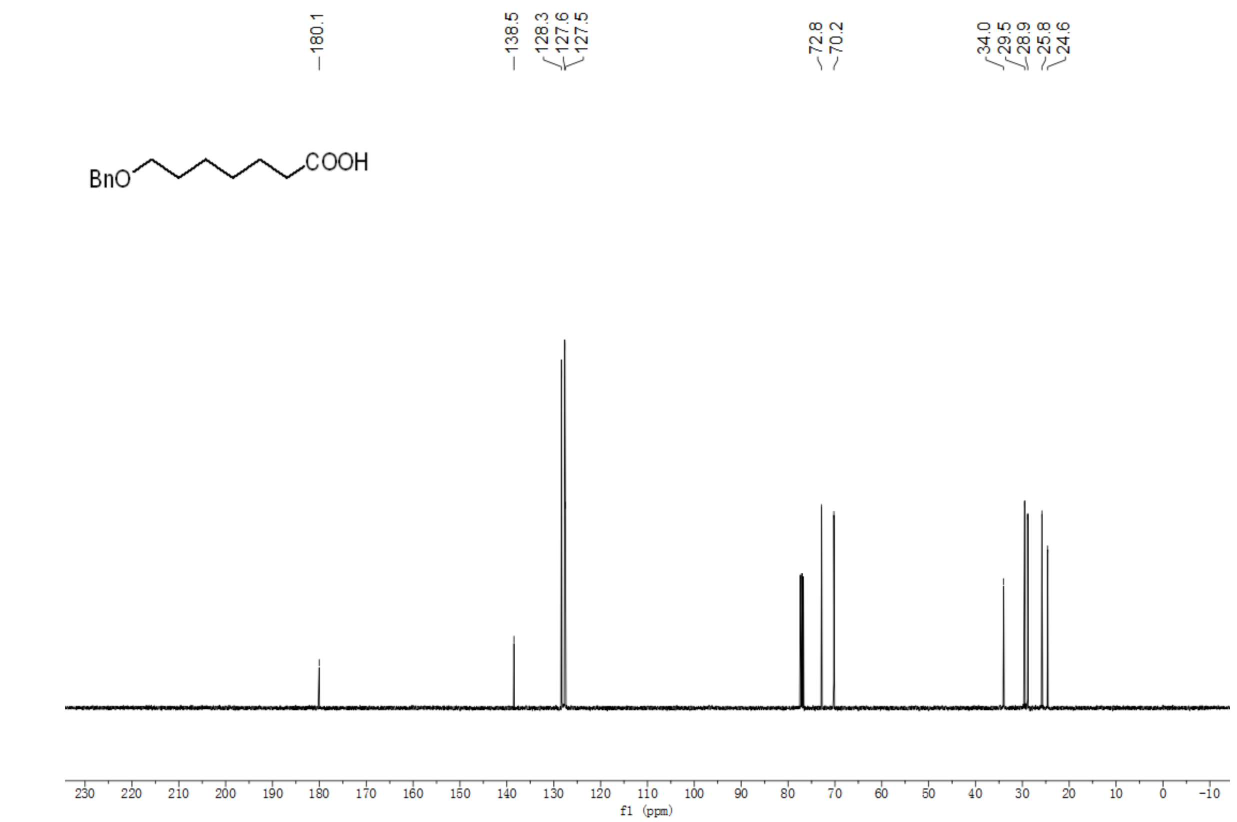

Supplementary Figure 78. ^{13}C NMR spectra of compound **6c**

4-phenylbutanoic acid (6d, *$CDCl_3$* as solvent)

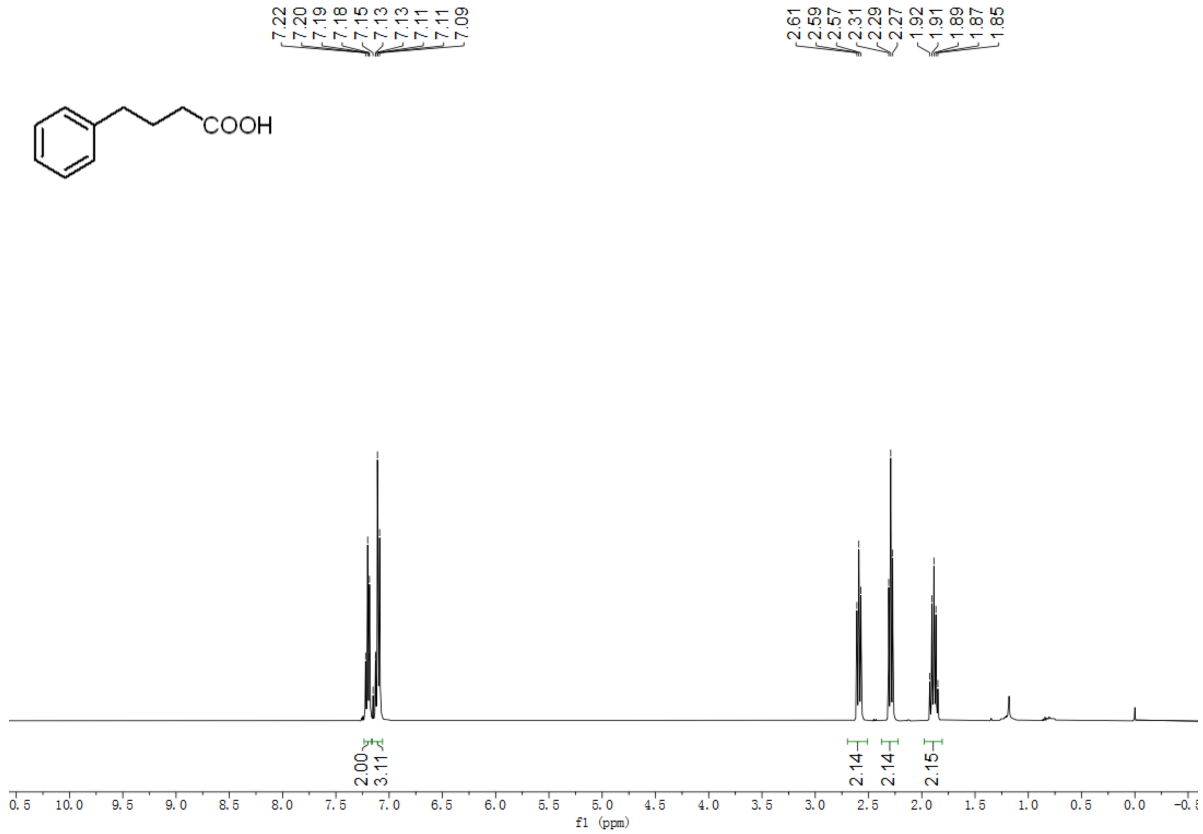

Supplementary Figure 79. ^{1}H NMR spectra of compound **6d**

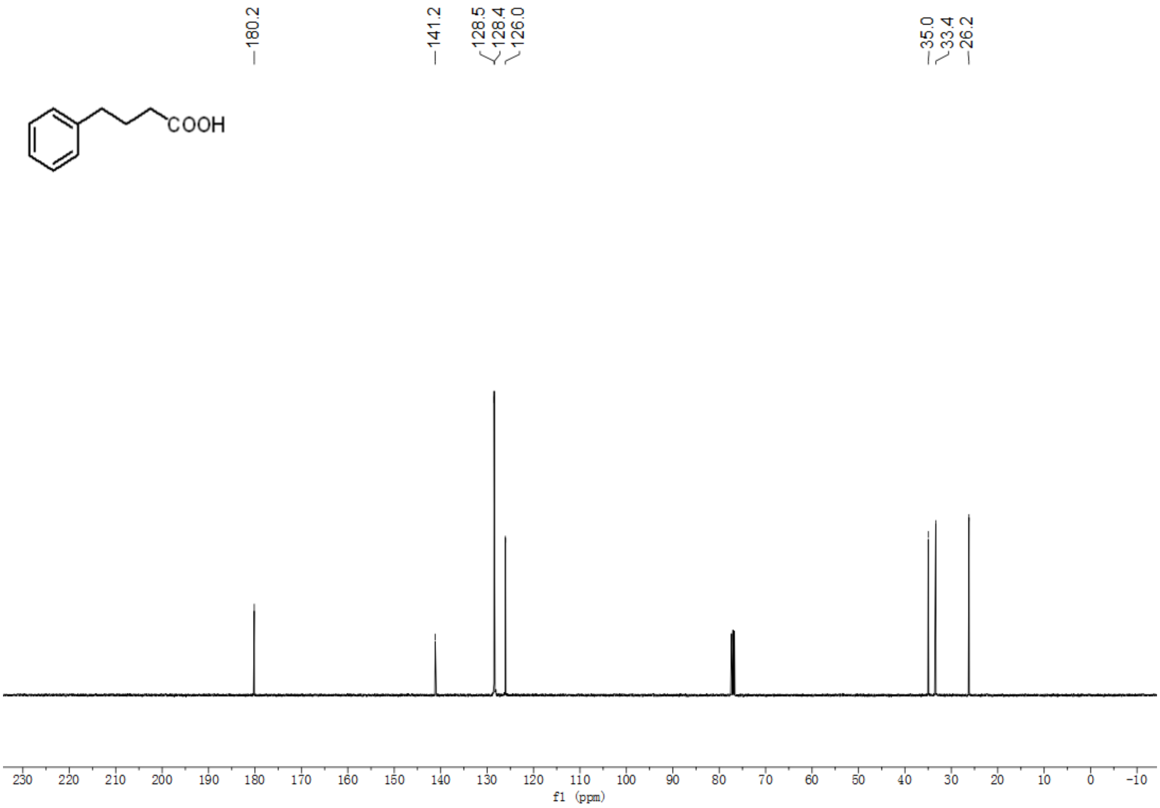

Supplementary Figure 80. ^{13}C NMR spectra of compound **6d**

Octanoic acid (6e, *$CDCl_3$* as solvent)

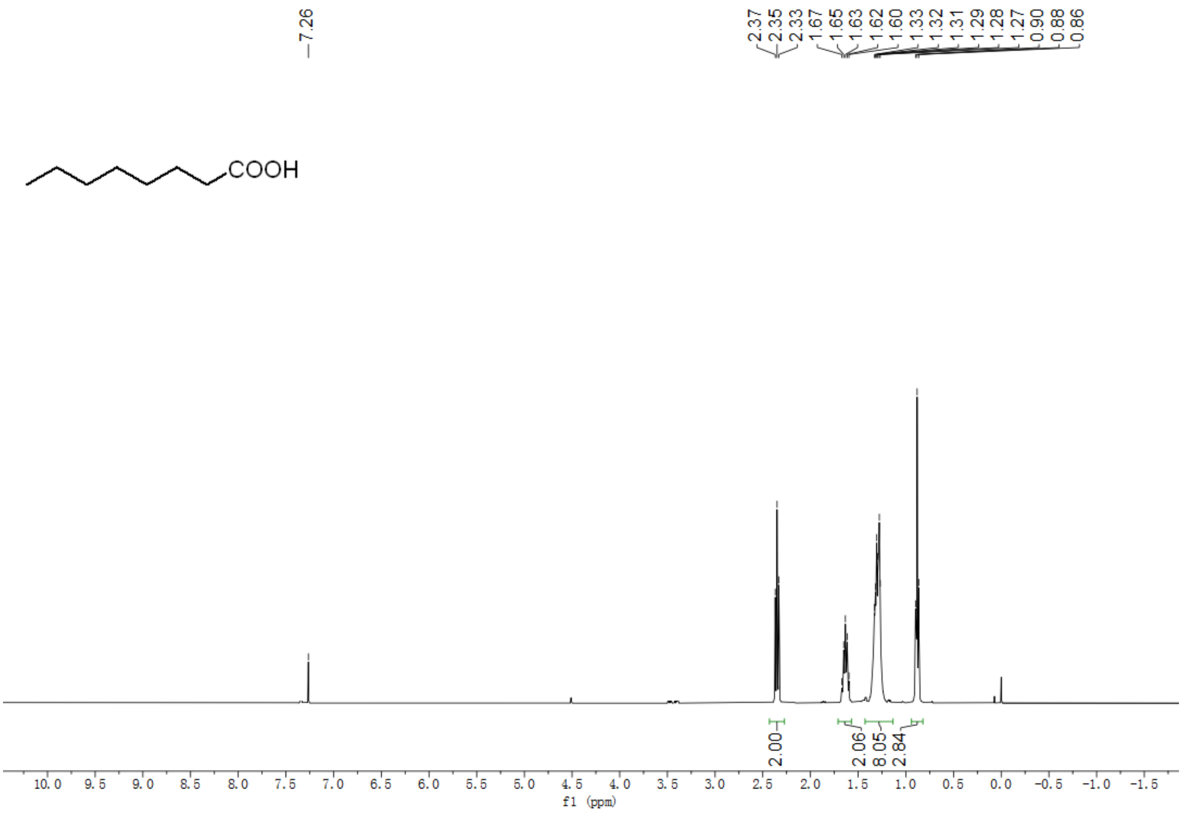

Supplementary Figure 81. ^{1}H NMR spectra of compound **6e**

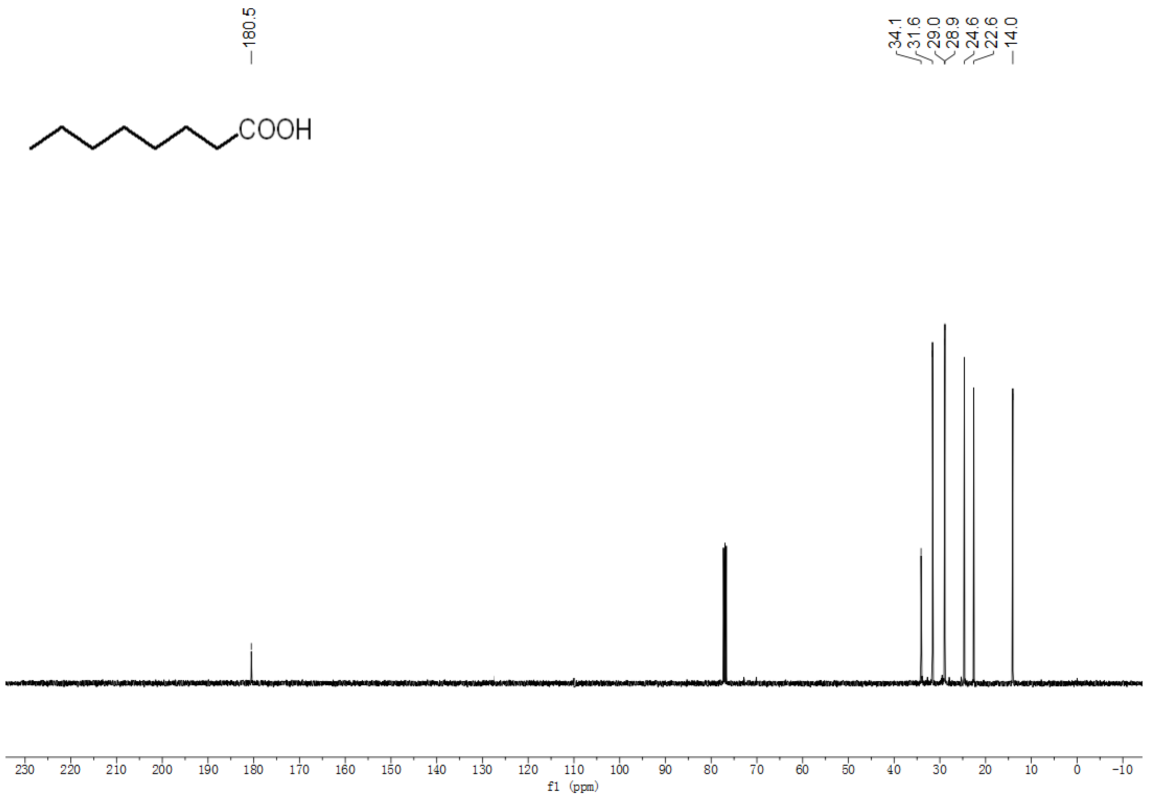

Supplementary Figure 82. ^{13}C NMR spectra of compound **6e**

3-ethylheptanoic acid (6f, *CDCl$_3$* as solvent)

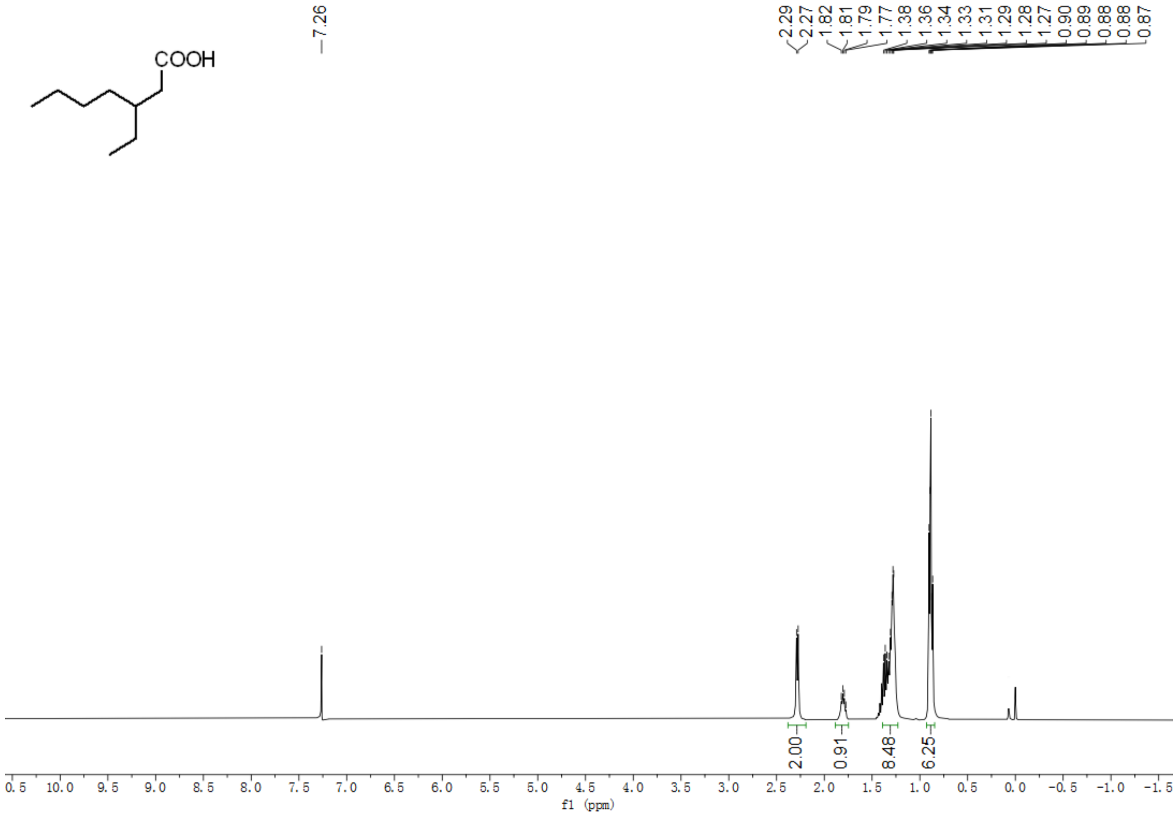

Supplementary Figure 83. ^{1}H NMR spectra of compound **6f**

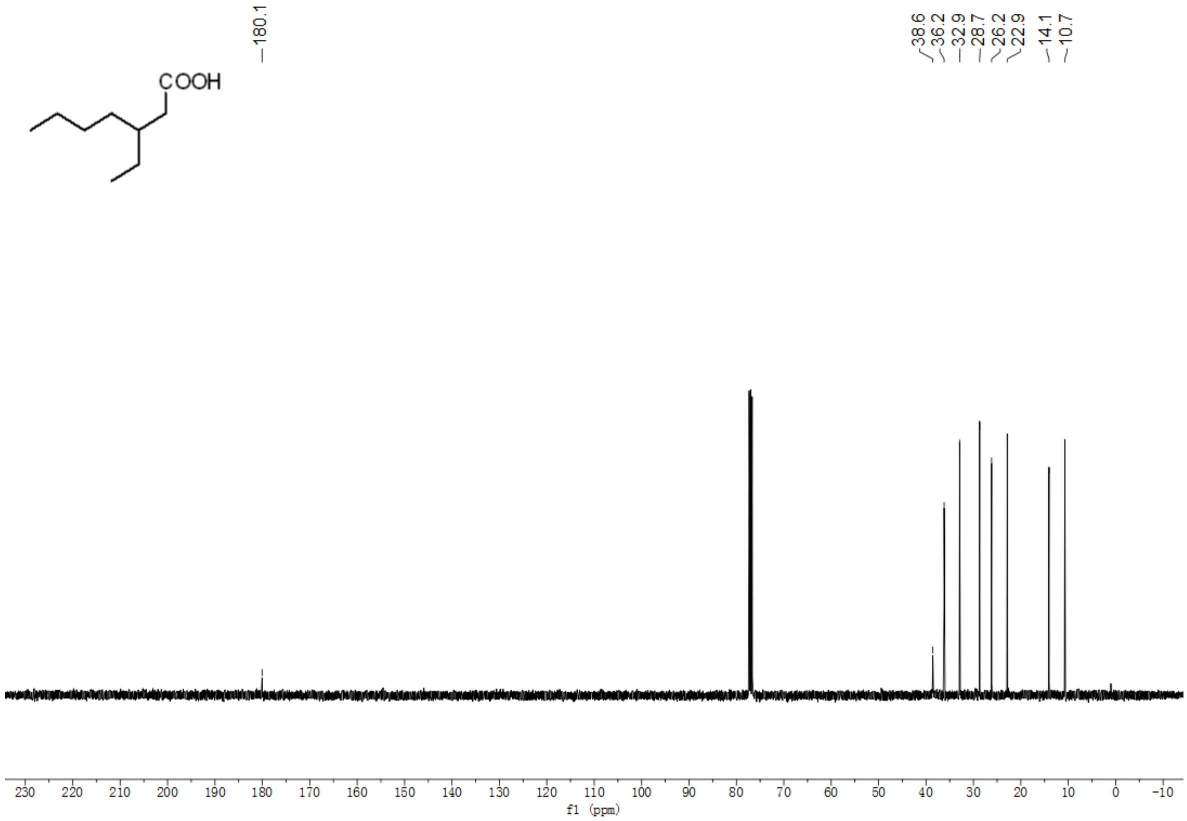

Supplementary Figure 84. ^{13}C NMR spectra of compound **6f**

6-methoxy-6-oxohexanoic acid (6g, *CDCl$_3$* as solvent)

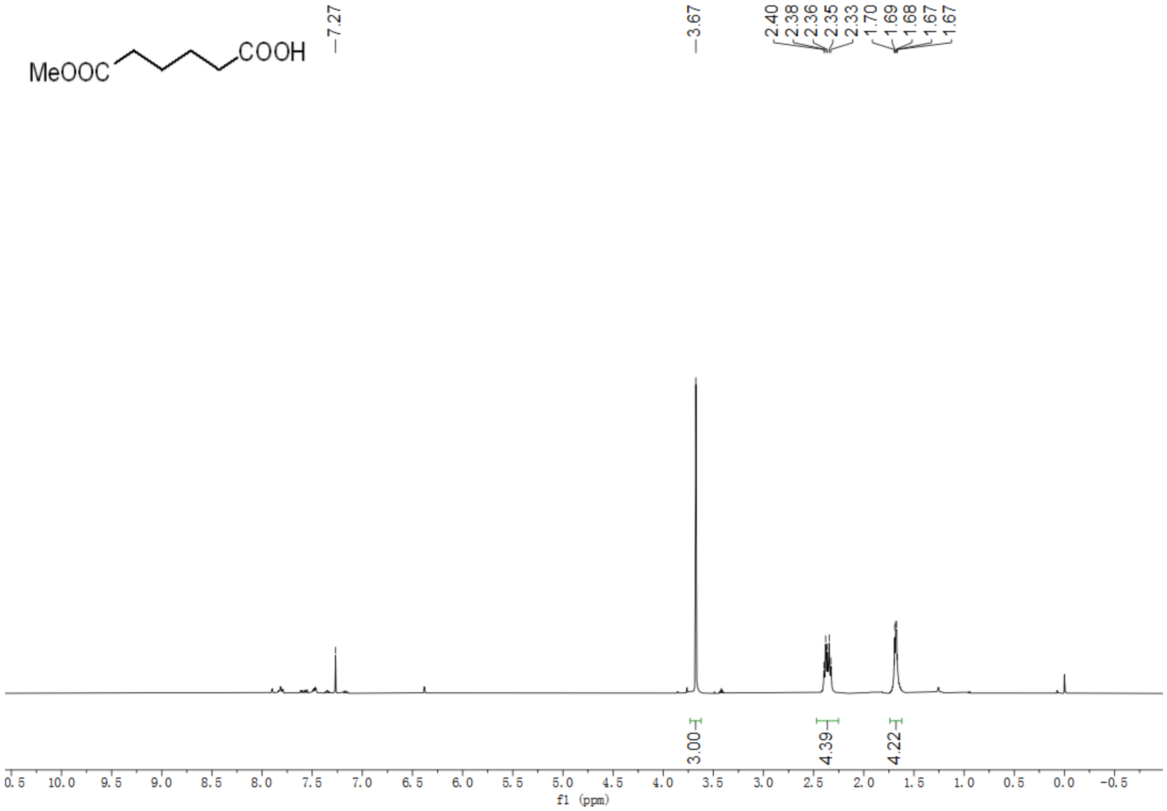

Supplementary Figure 85. ^{1}H NMR spectra of compound **6g**

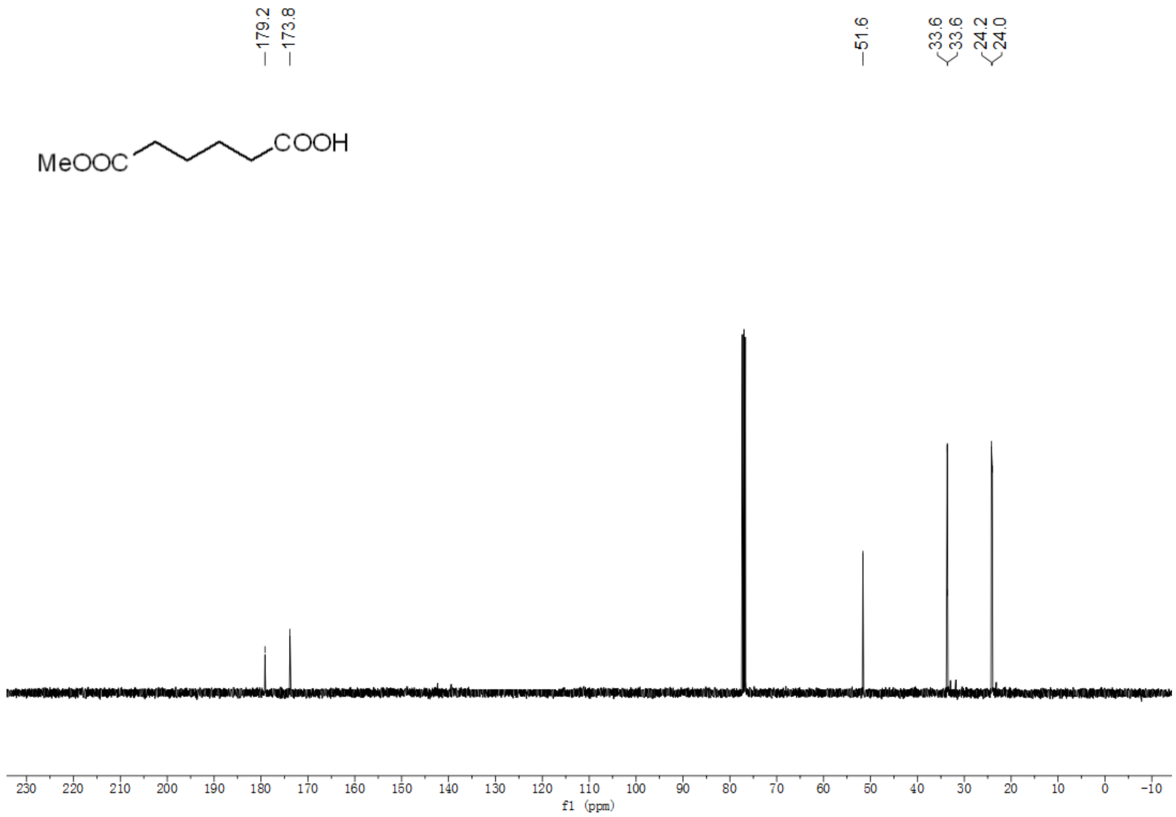

Supplementary Figure 86. ^{13}C NMR spectra of compound **6g**

Cyclohexanecarboxylic acid (6h, *$CDCl_3$* as solvent)

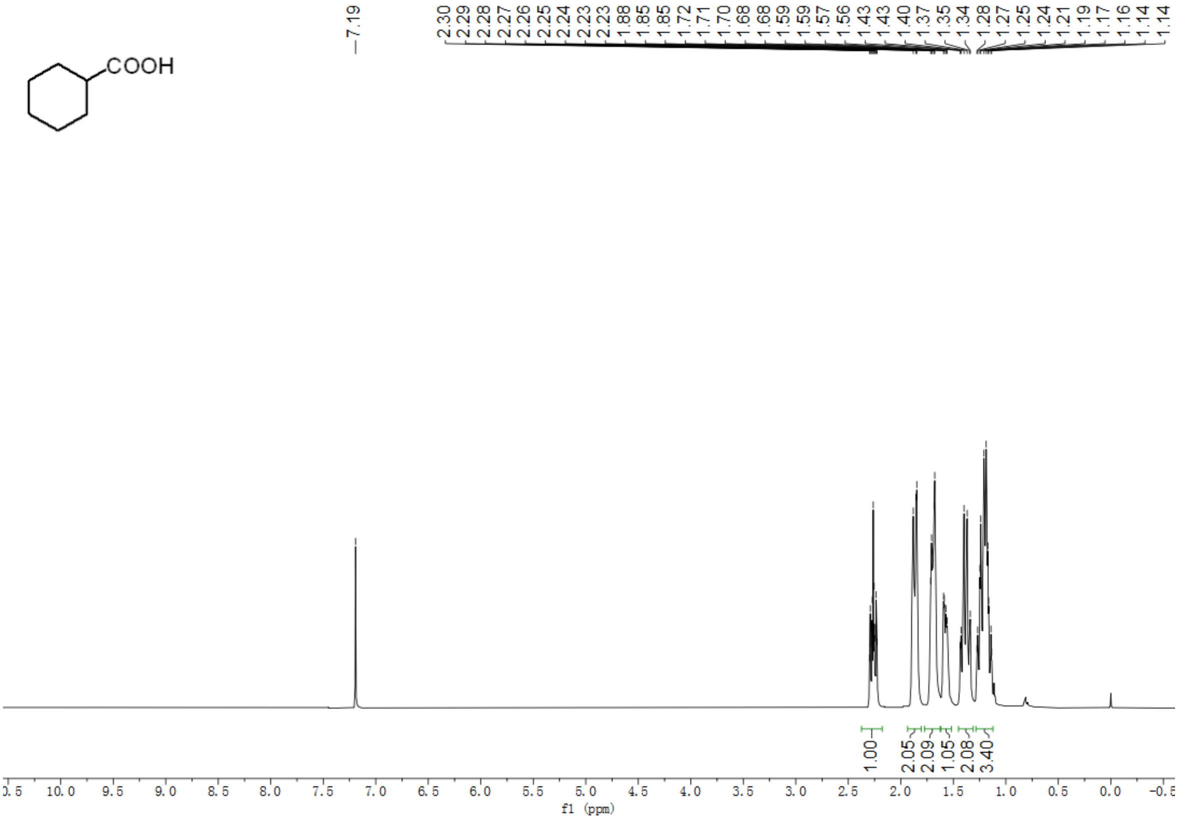

Supplementary Figure 87. ^{1}H NMR spectra of compound **6h**

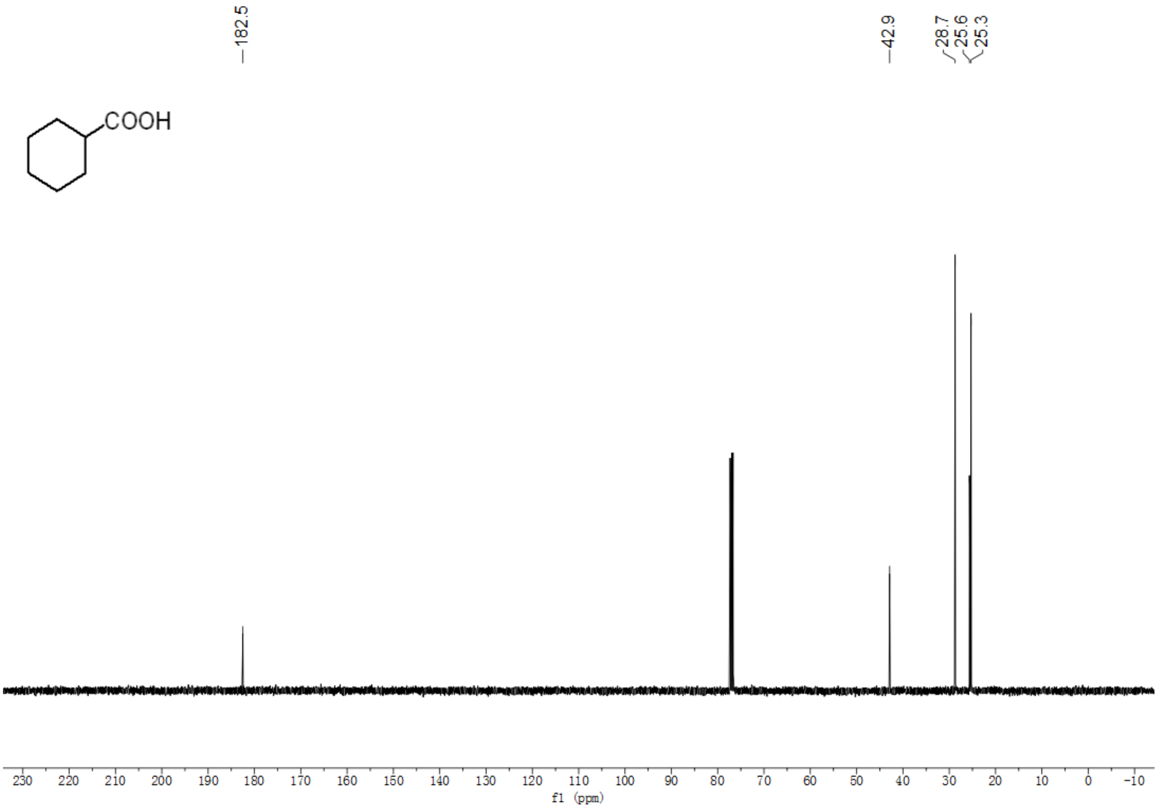

Supplementary Figure 88. ^{13}C NMR spectra of compound **6h**

Cycloheptanecarboxylic acid (6i, *CDCl₃* as solvent)

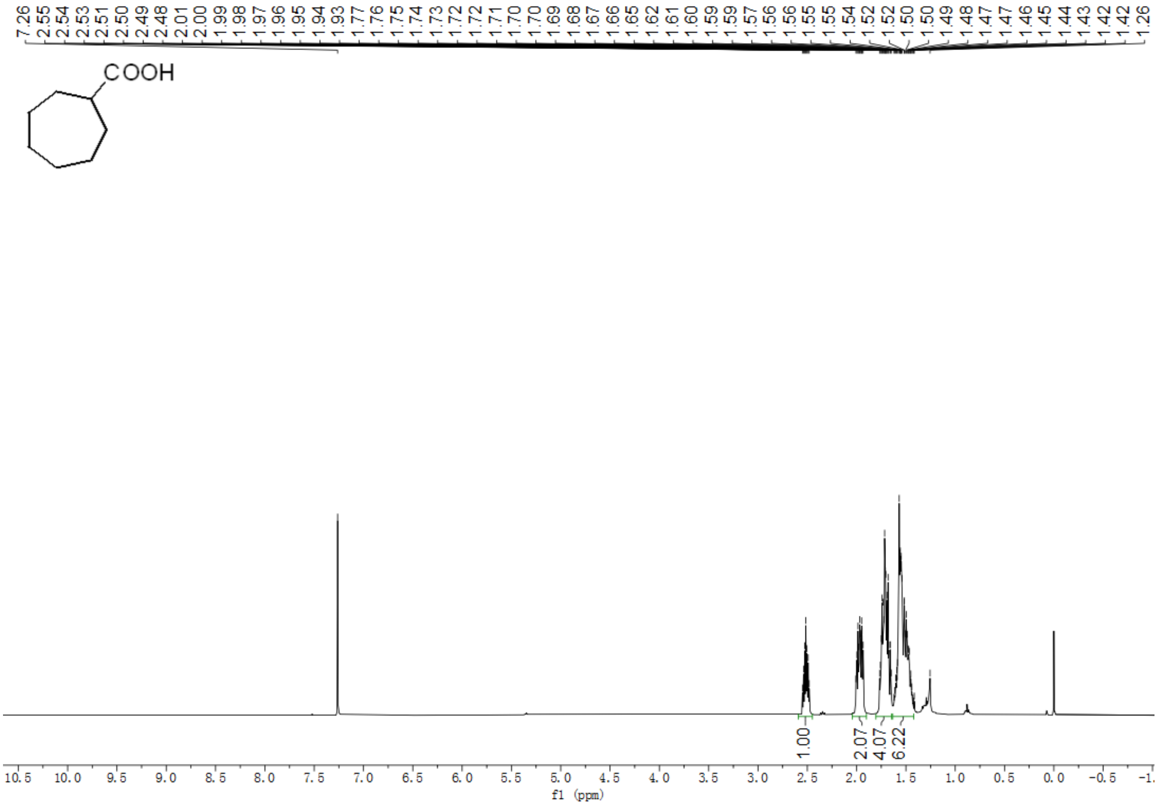

Supplementary Figure 89. ^{1}H NMR spectra of compound **6i**

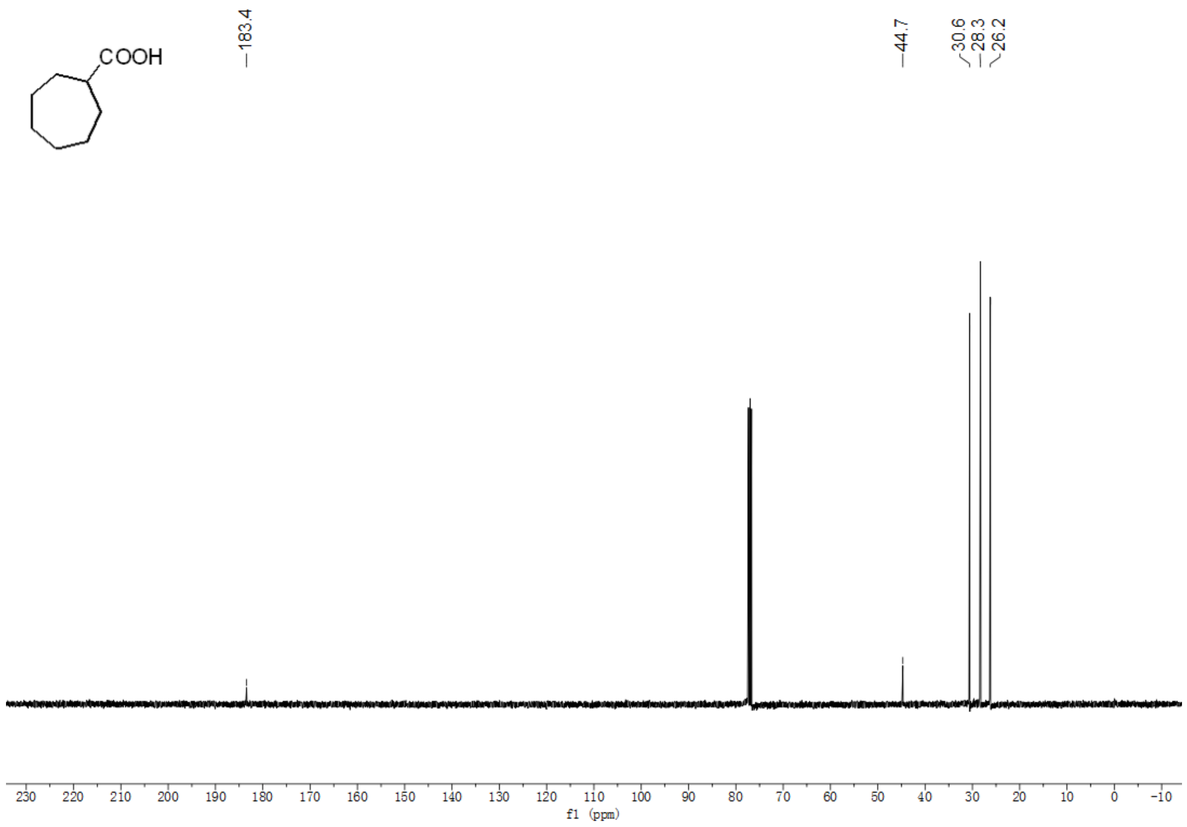

Supplementary Figure 90. ^{13}C NMR spectra of compound **6i**

Supplementary Reference

1. Gui, Y.-Y. *et al.* Coupling of C(sp^3)–H bonds with C(sp^2)–O electrophiles: mild, general and selective. *Chem. Commun.* **53**, 1192-1195 (2017).

2. Meng, Q.-Y., Wang, S. & König, B. Carboxylation of Aromatic and Aliphatic Bromides and Triflates with CO_2 by Dual Visible-Light–Nickel Catalysis. *Angew. Chem. Int. Ed.* **56**, 13426-13430 (2017).

3. Liu, J., Ren, Q., Zhang, X. & Gong, H. Preparation of Vinyl Arenes by Nickel-Catalyzed Reductive Coupling of Aryl Halides with Vinyl Bromides. *Angew. Chem. Int. Ed.* **55**, 15544-15548 (2016).

4. Chen, Z.-B., Liu, K., Zhang, F.-L., Yuan, Q. & Zhu, Y.-M. Palladium-catalyzed oxidative coupling of arylboronic acid with isocyanide to form aromatic carboxylic acids. *Org. Biomol. Chem.* **15**, 8078-8083 (2017).

5. Kopp, F., Wunderlich, S. & Knochel, P. Halogen–magnesium exchange on unprotected aromatic and heteroaromatic carboxylic acids. *Chem. Commun.*, 2075-2077 (2007).

6. Sierra, M. L. *et al.* Substituted 2-[(4-Aminomethyl)phenoxy]-2-methylpropionic Acid PPARα Agonists. 1. Discovery of a Novel Series of Potent HDLc Raising Agents. *J. Med. Chem.* **50**, 685-695 (2007).

7. Yang, Z. *et al.* Iridium-catalyzed highly efficient chemoselective reduction of aldehydes in water using formic acid as the hydrogen source. *Green Chem.* **19**, 3296-3301 (2017).

8. Schoeder, C. T. *et al.* Development of Chromen-4-one Derivatives as (Ant)agonists for the Lipid-Activated G Protein-Coupled Receptor GPR55 with Tunable Efficacy. *ACS Omega* **4**, 4276-4295 (2019).

9. Sun, G., Lv, X., Zhang, Y., Lei, M. & Hu, L. Palladium-Catalyzed Formylation of Aryl Iodides with HCOOH as CO Source. *Org. Lett.* **19**, 4235-4238 (2017).

10. Pi, J.-J. *et al.* Exploration of Biaryl Carboxylic Acids as Proton Shuttles for the Selective Functionalization of Indole C–H Bonds. *J. Org. Chem.* **83**, 5791-5800 (2018).

11. Barker, C. A. *et al.* Synthesis of new axially-disubstituted silicon-phthalocyanine derivatives: optical and structural characterisation. *Tetrahedron* **62**, 9433-9439 (2006).

12. Gao, Y. *et al.* Utilization of a Peptide Lead for the Discovery of a Novel PTP1B-Binding Motif. *J. Med. Chem.* **44**, 2869-2878 (2001).

13. Bazzi, S., Le Duc, G., Schulz, E., Gosmini, C. & Mellah, M. CO_2 activation by electrogenerated divalent samarium for aryl halide carboxylation. *Org. Biomol. Chem.* **17**, 8546-8550 (2019).

14. Liu, J., Fitzgerald, A. E. & Mani, N. S. Facile Assembly of Fused Benzo[4,5]furo Heterocycles. *J. Org. Chem.* **73**, 2951-2954 (2008).

15. Fujihara, T., Nogi, K., Xu, T., Terao, J. & Tsuji, Y. Nickel-Catalyzed Carboxylation of Aryl and Vinyl Chlorides Employing Carbon Dioxide. *J. Am. Chem. Soc.* **134**, 9106-9109 (2012).

16. Börjesson, M., Moragas, T. & Martin, R. Ni-Catalyzed Carboxylation of Unactivated Alkyl Chlorides with CO_2. *J. Am. Chem. Soc.* **138**, 7504-7507 (2016).

17. Jung, H.-Y., Chang, S. & Hong, S. Strategic Approach to the Metamorphosis of γ-Lactones to NH γ-Lactams via Reductive Cleavage and C–H Amidation. *Org. Lett.* **21**, 7099-7103 (2019).

18. Liu, K.-J. *et al.* Green and Efficient: Oxidation of Aldehydes to Carboxylic Acids and Acid Anhydrides with Air. *ACS Sustainable Chem. Eng.* **6**, 4916-4921 (2018).

19. Ren, W., Chu, J., Sun, F. & Shi, Y. Pd-Catalyzed Highly Chemo- and Regioselective Hydrocarboxylation of Terminal Alkyl Olefins with Formic Acid. *Org. Lett.* **21**, 5967-5970 (2019).

20. Zheng, W.; Morales-Rivera, C. A.; Lee, J. W.; Liu, P.; Ngai, M.-Y., Catalytic C−H Trifluoromethoxylation of Arenes and Heteroarenes. *Angew. Chem. Int. Ed.* **57**, 9645-9649 (2018).

21. Katsumi, I. *et al.* Studies on Styrene Derivatives. I.: Synthesis and Antiinflammatory Activities of α-Benzylidene-γ-butyrolactone Derivatives. *Chem. Pharm. Bull.* **34**, 121-129 (1986).

22. Gu, L. & Zhang, Y. Unexpected CO_2 Splitting Reactions To Form CO with N-Heterocyclic Carbenes as Organocatalysts and Aromatic Aldehydes as Oxygen Acceptors. *J. Am. Chem. Soc.* **132**, 914-915 (2010).

23. Nakamura, A. *et al.* A mild method for synthesizing carboxylic acids by oxidation of aldoximes using hypervalent iodine reagents. *Org. Biomol. Chem.* **16**, 541-544 (2018).
